# Tumor-localized CD40 agonism with MP0317, a FAP x CD40 DARPin, reprograms the tumor microenvironment in patients with advanced solid tumors: an open-label, nonrandomized, dose-escalation phase 1 study

**DOI:** 10.1038/s43018-026-01150-1

**Published:** 2026-05-01

**Authors:** Neeltje Steeghs, Carlos Gomez-Roca, Iphigénie Korakis, Eelke Gort, Hilde De Winter, Nina Stojcheva, Vaia Stavropoulou, Jennifer Krieg, Paul Baverel, Elena Fernandez, Ana Maria Florescu, Lea Hoenig, Michael Peter Sanderson, Vladimir Kirkin, Philippe Legenne, Philippe Alexandre Cassier

**Affiliations:** 1 Department of Medical Oncology, The Netherlands Cancer Institute, Amsterdam, the Netherlands; 2 Department of Medical Oncology, University Medical Center Utrecht, Utrecht, the Netherlands; 3 IUCT-Oncopole, Toulouse, France; 4 Molecular Partners AG, Schlieren-Zurich, Zurich, Switzerland; 5 Department of Medical Oncology, Centre Léon Bérard, Lyon, France

**Keywords:** Medical research, Cancer microenvironment, Tumour immunology, Cancer

## Abstract

This phase 1, open-label, nonrandomized, dose-escalation study evaluated MP0317 (FAP x CD40 DARPin) in adults with advanced solid tumors. Forty-six patients across nine cohorts received MP0317 at doses ranging from 0.03 to 10 mg kg^−1^ intravenously weekly or every 3 weeks. The primary outcome measure was safety; secondary and exploratory outcome measures included antitumor activity, pharmacokinetics and pharmacodynamics. Most treatment-related adverse events were of grades 1 and 2 (95%); a maximum tolerated dose was not reached. One patient achieved an unconfirmed partial response and 14 patients had stable disease. MP0317 serum pharmacokinetics confirmed extended half-life properties; terminal half-life estimates increased with dose and ranged from 21.8 to 120 h. Paired tumor biopsies confirmed colocalization of MP0317 with fibroblast activation protein and CD40. Activation of the CD40 pathway in the tumor microenvironment was shown by increased infiltration of antigen-presenting, plasma and follicular helper T cells, dendritic cell maturation, interferon-γ signaling and circulating immune markers. Collectively, these data confirm a favorable safety profile for MP0317 and support further clinical evaluation in combination with complementary immunotherapies. ClinicalTrials.gov registration: NCT05098405.

## Abstract (web summary)

In this paper, Steeghs and colleagues report the safety, activity, pharmacokinetic and pharmacodynamic results of a phase 1 dose-escalation trial with MP0317 (FAP x CD40 DARPin) monotherapy in patients with advanced solid tumors.

## Main

CD40 is a member of the tumor necrosis factor (TNF) receptor superfamily that bridges the innate and adaptive immune systems upon ligation on antigen-presenting cells (APCs), such as B cells, dendritic cells (DCs), monocytes and macrophages. Binding and activation of CD40 by its cognate ligand CD40L leads to upregulation of costimulatory molecules essential for T cell activation and proliferation. In concert with B cell receptor signaling, CD40 directly stimulates generation of antibody-producing cells, the humoral effector arm of adaptive immunity^1–3^. In addition, CD40 activation converts tumor-associated macrophages to activated macrophages with antitumor properties independent of T cells and alters the tumor stroma^4,5^.

Because of its strong immune-stimulatory activity, CD40 is an attractive target to realize the therapeutic potential of an antitumor immune response, but it also bears the risk of systemic toxicity. Indeed, systemically administered agonistic CD40 antibodies showed signs of activity in cancer patients, but their dosing was impaired by dose-limiting toxicity (DLT) with clinical manifestations of cytokine release syndrome, liver enzyme elevations or a range of other immune-related adverse reactions^6–8^. The observed liver toxicity may potentially arise from the activation of FcγR^+^ Kupffer cells, resident liver macrophages and sinusoidal endothelial cells^9^ by the Fc fragment of systemic anti-CD40 antibodies to mediate an inflammatory response^10–12^. In addition, such therapeutic antibodies can stimulate antibody-dependent cellular cytotoxicity toward APCs, as part of the Fc effector functions, potentially blunting the immune response^13^.

Another obstacle in the development of systemic CD40 agonists is target-mediated drug disposition (TMDD), which leads to rapid drug clearance from the bloodstream (that is, short half-life) and limits the drug’s distribution into lymph nodes and other tissues including the tumor microenvironment (TME)^14,15^. Higher doses may be required to overcome this CD40 sink effect and achieve relevant saturation of the CD40 receptor within the TME^7^. In turn, such doses of systemic CD40 agonists cannot be reached due to on-target toxicities.

Conversely, several preclinical studies using intratumoral or peritumoral injections of CD40-activating agents have shown that targeting CD40 specifically in the tumor site is well tolerated and effectively triggers antitumor responses^16,17^. Even though CD40 agonism was confined to the primary tumor, it still generated systemic tumor-specific T cell responses, which led to the elimination of secondary distant tumors and provided protection against tumor recurrence. These findings strongly support the strategy of directing anti-CD40 agonism specifically to the tumor.

**Extended Data Fig. 1:**
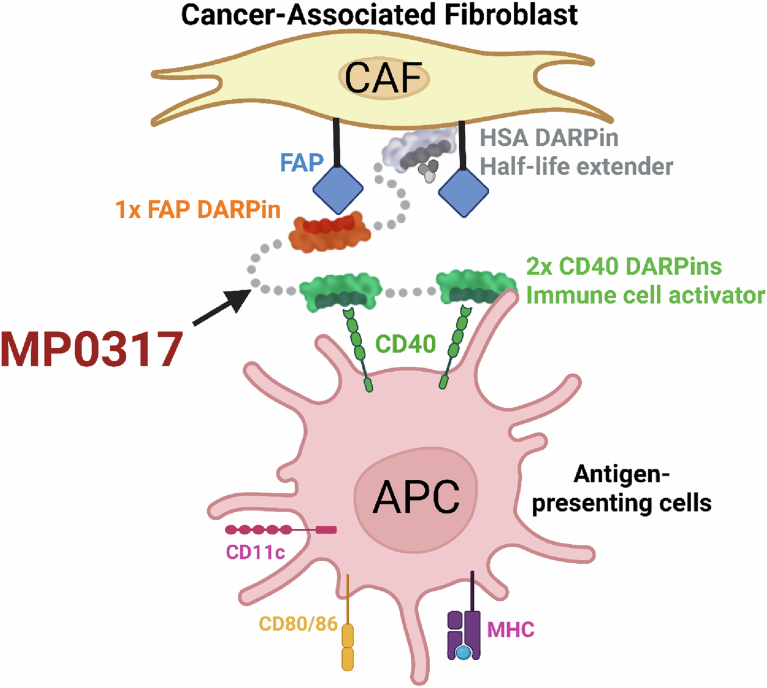
Schematic representation of the proposed MP0317 mode of action. APC, antigen-presenting cell; CAF, cancer-associated fibroblast; DARPin, Designed Ankyrin Repeat Protein; FAP, fibroblast activation protein; HSA, human serum albumin; MHC, major histocompatibility complex. Figure created in BioRender; Ribeiro, A. https://biorender.com/qko48xg (2025).

MP0317 (FAP x CD40) is a bispecific designed ankyrin repeat protein (DARPin) drug candidate^18^. MP0317 includes four covalently linked DARPin domains binding human fibroblast activation protein (FAP), human CD40 (two identical DARPin domains) and human serum albumin (for half-life extension) (Extended Data Fig. 1). It is a non-antibody-based CD40 agonist, hence without Fc fragment and Fc effector function, designed to activate immune cells specifically within the TME, thereby potentially reducing toxicity compared to systemic CD40 agonistic antibody approaches and consequently allowing a broader therapeutic window to achieve effective dosing. MP0317 activates CD40 selectively on APCs within the TME, independent of FcR-mediated cross-linking, by engaging with FAP expressed on cancer-associated fibroblasts in the tumor stroma. Thus, MP0317 is intended to induce immune activation only when clustered in the TME^19^ or possibly lymph nodes, which may express FAP on their reticular network^20,21^. FAP is involved in several hallmarks of cancer, including proliferation and invasiveness, extracellular matrix remodeling, tumor vascularization and escape from immunosurveillance^19^. It is highly present in neoplastic lesions but has limited expression in healthy tissues^22–25^, making it an ideal candidate for targeted cancer therapy. The FAP x CD40 DARPin design was confirmed in vitro to conditionally activate the CD40 pathway on B cells, DCs and macrophages only in the presence of FAP-expressing cells. In vivo, a surrogate DARPin binding murine FAP and CD40 delivered potent antitumor activity in a FAP-expressing MC38 colorectal tumor model in immune-competent mice. In contrast to an anti-CD40 antibody, this surrogate FAP x CD40 DARPin did not induce elevation of liver enzymes or systemic cytokine release in mice^18^.

In this article, we report the results of the first-in-human phase 1 dose-escalation study (NCT05098405) evaluating MP0317 monotherapy in patients with advanced solid tumors.

## Results

### Patient population and treatment

**Fig. 1:**
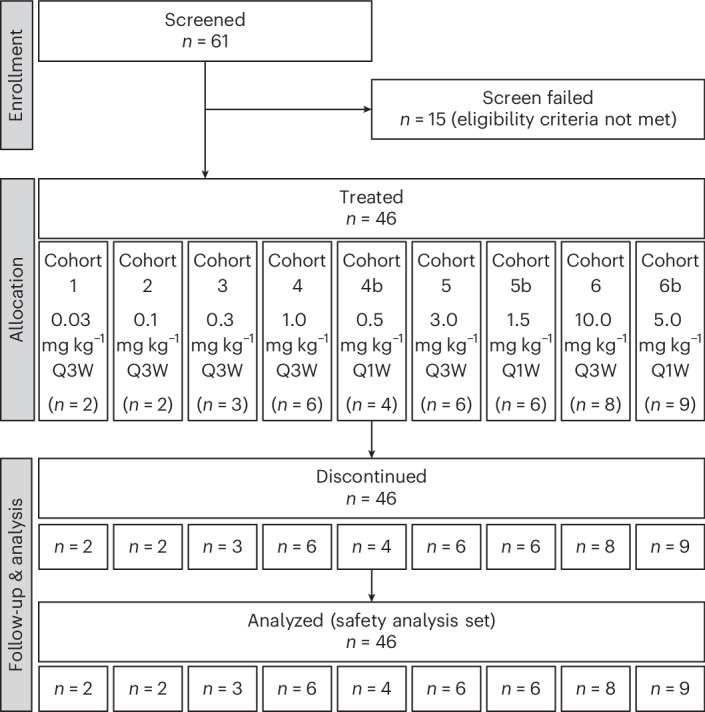
Patient disposition. Q1W, weekly dosing; Q3W, dosing every 3 weeks.

Sixty-one patients with relapsed/refractory advanced solid tumors were screened (Fig. 1; further details on eligibility criteria are shown in Methods). Of these, 15 patients did not meet the eligibility criteria; the remaining 46 were enrolled and treated until disease progression (*n* = 42), withdrawal by patient (*n* = 2), unacceptable toxicity (*n* = 1) or investigator decision (*n* = 1).

All 46 patients (*n* = 24 female, *n* = 22 male; median age: 63 years (range 35–79)) had an Eastern Cooperative Oncology Group performance status (ECOG PS) of 0 (*n* = 22) to 1 (*n* = 24) and were heavily pretreated with a median of four (range: 1–13) prior lines of treatment (including 15 patients with prior immune checkpoint inhibitor (CPI) treatment). The most common tumor types were colorectal cancer (*n* = 12, including *n* = 11 with microsatellite stable and *n* = 1 with microsatellite instable-high status), pancreatic cancer (*n* = 9), mesothelioma (*n* = 6) and non-small-cell lung cancer (NSCLC) (*n* = 4) (Table 1).

**Table 1:** Patient baseline characteristics

Baseline characteristic	Patients (*n* = 46)
Age (years), median (range)	63 (35–79)
Sex, *n* (%)	
Female	24 (52)
Male	22 (48)
ECOG PS, *n* (%)	
0	22 (48)
1	24 (52)
Number of prior regimens, median (range)	4 (1–13)
Prior CPI, *n* (%)	15 (33)
Cancer types, *n* (%)	
Colorectal	12 (27)
Pancreatic	9 (20)
Mesothelioma	6 (13)
NSCLC	4 (9)
Breast	3 (7)
Endometrial	3 (7)
GIST	2 (4)
Ovarian	2 (4)
Other^a^	5 (11)
Time since cancer diagnosis (months), mean (s.d.)	50.1 (41.3)
Time since last progression (months), mean (s.d.)	2.8 (3.7)

^a^Other cancer types affecting one patient each: cervical cancer, cholangiocarcinoma, squamous cell carcinoma (SCC) of the esophagus, bladder cancer and SCC of the anus.

MP0317 was given intravenously every 3 weeks (Q3W); six dose levels were explored: 0.03, 0.1, 0.3, 1.0, 3.0 and 10 mg kg^−1^. In addition, based on emerging pharmacokinetic (PK) data, three dose levels were explored in a weekly regimen (Q1W): 0.5, 1.5 and 5 mg kg^−1^. Patients stayed on treatment for a median time of 5 weeks (range: 1–21).

### Safety

All 46 patients received at least one dose of MP0317 (safety analysis set) and a total of 228 infusions. Overall, 415 treatment-emergent adverse events were reported in all 46 patients, with most not being treatment-related. In total, 118 treatment-related adverse events (TRAEs) were reported in 37 patients (Table 2). Of these, six (5%) were of National Cancer Institute Common Terminology Criteria for Adverse Events grade 3, occurring in four patients (9%). There were no TRAEs greater than grade 3. The most frequently observed TRAEs were fatigue (reported in 15 patients (33%), grades 1 and 2), infusion-related reactions (IRRs) (reported in eight patients (17%), grade 2), nausea (reported in seven patients (15%), grades 1 and 2), transaminase elevation (reported in five patients (11%), grades 1–3), anorexia (reported in five patients (11%), grades 1 and 2) and vomiting (reported in four patients (9%), grades 1 and 2). One patient discontinued study treatment because of a TRAE: a recurring grade 2 IRR.

**Table 2:** Overview of the MP0317 safety profile

Number of TRAEs (number of patients)										
Cohort no.	1	2	3	4	4b	5	5b	6	6b	Total
MP0317 dose level	0.03 mg kg^−1^ Q3W	0.1 mg kg^−1^ Q3W	0.3 mg kg^−1^ Q3W	1 mg kg^−1^ Q3W	0.5 mg kg^−1^ Q1W	3 mg kg^−1^ Q3W	1.5 mg kg^−1^ Q1W	10 mg kg^−1^ Q3W	5 mg kg^−1^ Q1W	
Number of patients per cohort	2	2	3	6	4	6	6	8	9	46
TRAEs	1 (1)	10 (2)	4 (3)	20 (5)	13 (3)	5 (4)	29 (6)	26 (6)	10 (7)	118 (37)
Grade 3 TRAEs	0 (0)	0 (0)	0 (0)	0 (0)	2 (2)	0 (0)	1 (1)	3 (1)	0 (0)	6 (4)
Most frequent TRAEs										
Fatigue	0 (0)	1 (1)	0 (0)	2 (2)	1 (1)	1 (1)	5 (5)	4 (2)	3 (3)	17 (15)
IRR	1 (1)	1 (1)	0 (0)	3 (1)	2 (1)	1 (1)	1 (1)	2 (1)	1 (1)	12 (8)
Nausea	0 (0)	0 (0)	0 (0)	2 (2)	1 (1)	0 (0)	1 (1)	3 (3)	0 (0)	7 (7)
Transaminases increased	0 (0)	0 (0)	0 (0)	2 (2)	1 (1)	0 (0)	0 (0)	6 (1)	1 (1)	10 (5)
Anorexia	0 (0)	1 (1)	0 (0)	2 (2)	0 (0)	0 (0)	1 (1)	0 (0)	1 (1)	5 (5)
Vomiting	0 (0)	0 (0)	0 (0)	1 (1)	0 (0)	0 (0)	3 (2)	1 (1)	0 (0)	5 (4)
Serious TRAEs	0 (0)	0 (0)	0 (0)	1^a^ (1)	1^b^ (1)	0 (0)	0 (0)	2^c^ (1)	1^a^ (1)	5 (4)

^a^Grade 2 IRR with hospitalization for patient monitoring.

^b^Grade 1 heart failure.

^c^Isolated asymptomatic grade 3 alanine aminotransferase and aspartate aminotransferase increased, DLT, upgraded to SAE by sponsor. The TRAE row ‘Transaminases increased’ includes four preferred terms as per the Medical Dictionary for Regulatory Activities (MedDRA): ‘alanine aminotransferase increased’, ‘aspartate aminotransferase increased’, ‘transaminases increased’ and ‘hepatic cytolysis’.

Five serious AEs (SAEs) related to the study drug were reported in four (9%) patients. Two of these SAEs were grade 2 IRRs reported in two patients, which led to the patients’ hospitalization for monitoring purposes. One SAE was a grade 1 asymptomatic heart failure, identified during routine protocol assessments as a reduction of left ventricular ejection fraction from 59% at baseline to 52%, in a patient with breast carcinoma, which had been treated before with a range of systemic treatments, including anthracyclines, as well as with radiotherapy. The patient had entered the study with asymptomatic mild cardiac insufficiency, New York Heart Association stage II. The two remaining SAEs were asymptomatic increases of grade 3 aspartate aminotransferase and alanine aminotransferase, both reported simultaneously in a single patient treated at the highest MP0317 dose of 10 mg kg^−1^ Q3W. These grade 3 liver enzyme increases were reported as nonserious by the treating investigator but were upgraded to serious by the sponsor and qualified as DLT. No further DLTs were observed in the study. The maximum tolerated dose (MTD) of MP0317 was not reached in the study.

### Antitumor activity

**Fig. 2:**
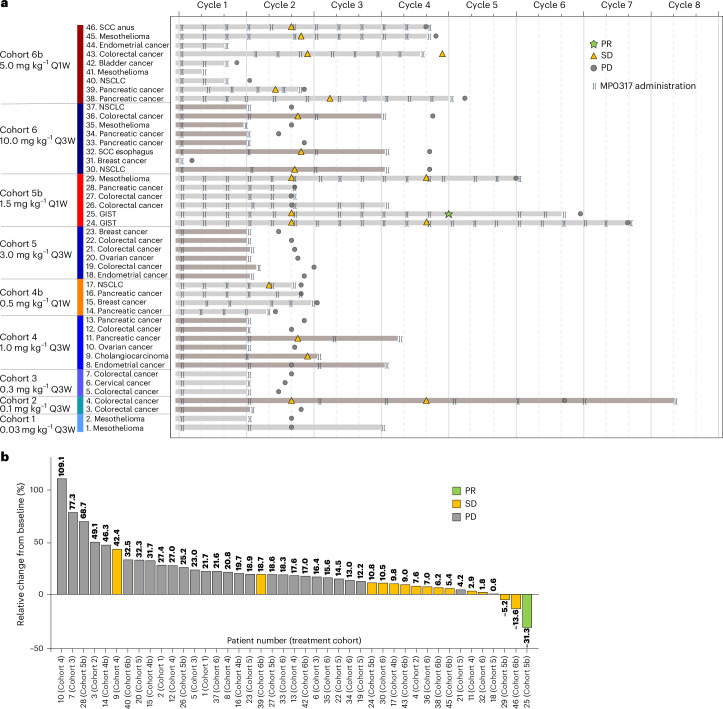
MP0317 treatment and clinical outcomes. **a**, Treatment duration and Response Evaluation Criteria in Solid Tumors (RECIST) v.1.1 (overall response)/iRECIST assessments per patient over time. **b**, Maximal change in the sum of longest diameter of the target lesions and best overall response according to RECIST v.1.1 (overall response)/iRECIST (safety analysis set). Patient 9 was assessed by the investigator as having achieved SD during first on-treatment TA despite the high increase in the sum of diameters of the target lesions (possible pseudo-progression). Treatment continued after the first TA until progression was confirmed during a second TA 27 days later. Patient 31 is not depicted because the target lesions were not measured during on-treatment TA (not evaluable); however, a new non-target lesion was documented in the central nervous system. Not depicted are also patient 41 (no on-treatment TA performed but clinical pharmacodynamics (PD) confirmed) and patient 44 (withdrawal by patient before scheduled on-treatment TA) iRECIST, modified RECIST in cancer immunotherapy studies. Source data

All 46 treated patients from the safety analysis set were included in the antitumor activity analysis. One patient with gastrointestinal stromal tumor (GIST) in cohort 5b (MP0317 1.5 mg kg^−1^ Q1W) achieved an unconfirmed partial response (PR), which resulted in an unconfirmed overall response rate (ORR) of 2% (90% confidence interval (CI) 0.1 to 11.5). Fourteen patients (30%) achieved stable disease (SD), of which two also showed quantifiable target lesion shrinkage, resulting in a disease control rate (DCR) of 33% at first tumor assessment (TA) during treatment (at approximately 4–6 weeks; Fig. 2a). Five patients continued to show disease control at day 90 (4 SD, 1 PR achieved; CDR 11%). By day 180, all patients had progressed or discontinued the study. Relative change of target lesion size from baseline per patient is shown in Fig. 2b. Twenty-nine patients (63%) had progressive disease, while two (4%) patients were not evaluable. The median progression-free survival (PFS) was similar across the two dosing schedules, with 38.5 days (95% CI 35.0 to 80.0 days) in Q1W dosed patients, and 36.0 days (95% CI 35.0 to 39.0) in Q3W dosed patients.

### PK and immunogenicity

**Fig. 3:**
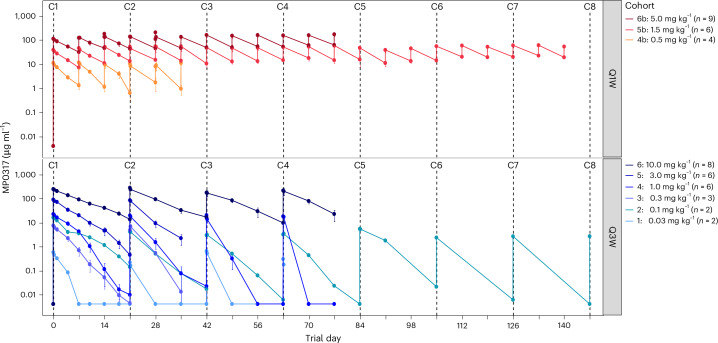
MP0317 serum PK profile. MP0317 serum concentrations during treatment. Bottom: cohorts treated with MP0317 infusion every 3 weeks (Q3W: 0.03–10 mg kg^−1^). Top: cohorts treated with weekly infusion of MP0317 (Q1W: 0.5–5 mg kg^−1^). All values represent the arithmetic mean and s.e.m. The s.e.m. was calculated based on the number of patients per cohort. Source data

After repeated intravenous infusions, MP0317 serum concentrations decreased in a mono-exponential fashion in most cohorts and remained detectable until the end of the dosing interval with moderate inter-individual variability (Fig. 3). PK parameters (Supplementary Table 1) obtained using noncompartmental analysis confirmed that the serum PK of MP0317 was linear for the maximum serum concentration (*C*_max_) across the tested dose ranges of 0.03 to 10 mg kg^−1^ Q3W and 0.5 to 5 mg kg^−1^ Q1W, while the area under the curve (AUC) showed a greater than dose-proportional increase, which was consistent with the impact of a CD40-driven antigen sink on exposure levels at lower doses. The mean volume of distribution at steady state (*V*_ss_) remained consistent across doses with values approximating the serum volume, suggesting that MP0317 biodistribution in healthy tissue was primarily limited to the circulatory system. Mean terminal half-life (*t*_1/2_) estimates of MP0317 increased with dose, with values ranging from 21.8 h (0.03 mg kg^−1^) to 120 h (10 mg kg^−1^) in the Q3W cohorts, and from 60.2 h (0.5 mg kg^−1^) to 103 h (5 mg kg^−1^) in the Q1W cohorts, confirming the extended half-life properties of MP0317 (ref. ^18^), which confer a PK profile suitable for Q1W or Q3W dosing.

In 85% of the study population (93% in the Q3W cohorts and 75% in the Q1W cohorts), antidrug antibodies (ADAs) against MP0317 were detected at least once during study treatment (Supplementary Table 2). There was no correlation between ADA titer and MP0317 dose. Moreover, at the two highest Q3W regimen doses (3 mg kg^−1^ and 10 mg kg^−1^) and in all three Q1W dosing cohorts (0.5 mg kg^−1^, 1.5 mg kg^−1^ and 5 mg kg^−1^), a sustained MP0317 exposure was observed, overcoming TMDD and the impact of ADAs. ADAs, if occurring, were detected until discontinuation from the study.

### Pharmacodynamics

**Fig. 4:**
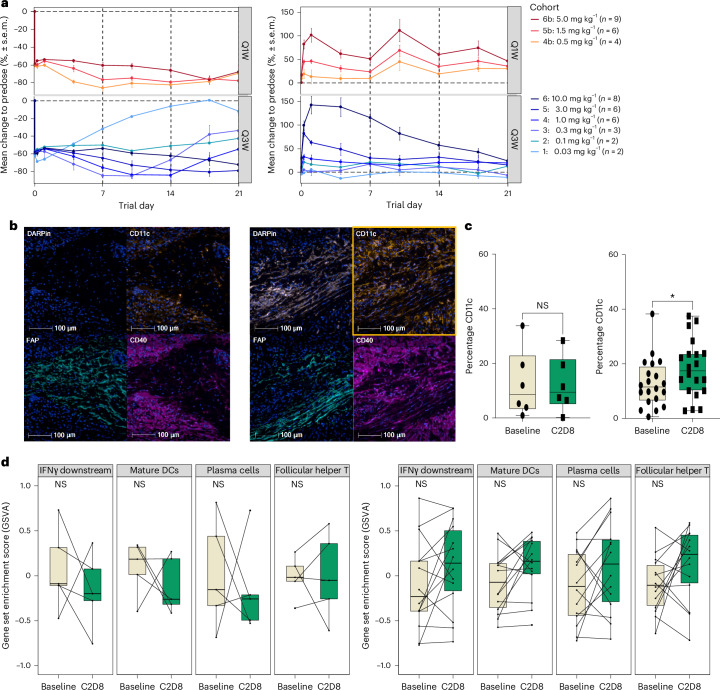
Pharmacodynamic effects of MP0317 in blood and on tumor tissue. **a**, Mean percentage change from baseline in free FAP (left) and CD40 (right) serum concentrations indicated peripheral target engagement. Bottom: MP0317 Q3W (0.03–10 mg kg^−1^). Top: MP0317 Q1W (0.5–5 mg kg^−1^). Values show the arithmetic mean ± s.e.m., calculated per cohort. **b**, mIF staining for MP0317 (DARPin), CD40, FAP and CD11c in a representative patient with GIST receiving 1.5 mg kg^−1^ MP0317 Q1W at screening (left) and on C2D8 (right). The tumor area was verified on hematoxylin-eosin and pan-cytokeratin images in the biopsied peritoneal metastasis lesion. DC (CD11c^+^) abundance increased at C2D8 (orange box). MP0317 was detected in 22 of 26 patients with evaluable paired tumor biopsies, with MP0317-CD40-FAP colocalization in 21 of 26. Increased DC abundance occurred in 20 of 26 patients. **c**, Tumor DC abundance according to MP0317 treatment group. Percentage of CD11c^+^ cells among nucleated cells scored using mIF in evaluable paired biopsies (*n* = 26). Left: MP0317-low dose group (*n* = 6). Right: MP0317-higher dose group (*n* = 20). Each dot (baseline) and square (after treatment, C2D8) represents a single biopsy. Upper (75%), median and lower (25%) percentiles are indicated. *P* values (*P* = 1 and *P = 0.0266, respectively) were derived from paired, two-sided Wilcoxon rank-sum tests. **d**, Gene signatures indicating CD40 pathway activation according to MP0317 treatment group. Gene set enrichment scores at baseline and C2D8 for patients with paired biopsies available for transcriptomic analysis (*n* = 19). Left: MP0317-low dose group (*n* = 5). Right: MP0317-higher dose group (*n* = 14). Each dot indicates a single biopsy. Box plots are shown with the upper (75%), median and lower (25%) percentiles. The upper whiskers extend to the largest value no further than 1.5 times the interquartile range from the box plot hinge. The lower whiskers extend to the smallest value at most 1.5 times the interquartile range of the box plot hinge; isolated points show outliers. *P* values were derived from paired, two-sided Wilcoxon rank-sum tests; no adjustment was made for multiple comparisons. NS, not significant. Source data

The effect of MP0317 on soluble biomarkers was assessed with immunoassays. Free soluble FAP (sFAP) and CD40 (sCD40) levels in patient serum after treatment were modulated in a manner indicative of MP0317 target engagement in the periphery (Fig. 4a). sFAP decreased soon after MP0317 administration in a dose-dependent manner. At MP0317 doses of 1.5 mg kg^−1^ and greater, sFAP levels were durably suppressed, which was indicative of target saturation in the periphery. Levels of sCD40 were stable at doses below 5 mg kg^−1^, while doses of 5 mg kg^−1^ and greater induced a pronounced transient increase in sCD40, potentially reflecting the induction of CD40 shedding^26^.

Multiplex immunofluorescence (mIF) analysis demonstrated that MP0317 was present in the tumor biopsies of 85% of patients (22 of 26) with evaluable paired tumor biopsies. Colocalization of MP0317 with FAP and CD40 was observed in 81% of these patients (21 of 26) (Fig. 4b).

**Extended Data Fig. 2:**
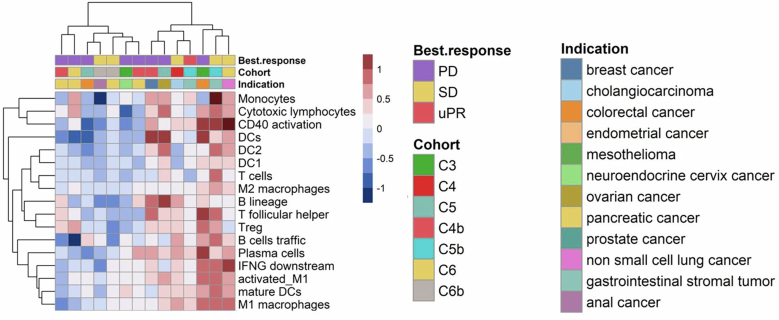
MP0317-induced changes in gene signatures in the tumor microenvironment. Changes in gene set enrichment scores for signatures associated with the tumor microenvironment for patients in the MP0317-higher dose group. Scores were computed using the GSVA package in R, and the changes are defined as the difference between post- and pre-treatment. Source data

To explore the dynamic effects of MP0317 on the tumor-associated immune cell landscape, patients were separated into two groups according to an a priori exploratory analysis plan. The first group (hereafter referred to as ‘MP0317-low dose’) consisted of six patients treated with non-pharmacologically active doses according to PK/PD modeling (≤0.1 mg kg^−1^, cohorts 1 and 2, *n* = 4) or those who lacked detectable MP0317 in the tumor biopsy after dosing (cohort 4, 1 mg kg^−1^ Q3W, *n* = 2), as assessed using mIF. The second group (hereafter called ‘MP0317-higher dose’) consisted of patients treated with the ≥0.3 mg kg^−1^ dose (cohorts 3 and higher) and who had confirmed detection of MP0317 in the tumor biopsy after dosing (*n* = 20). Patients in the MP0317-higher dose group demonstrated a significant increase in tumor abundance of DCs (*P* = 0.0266), as assessed using mIF analysis of CD11c^+^ cells (Fig. 4c). Consistent with this, bulk transcriptomic analyses from evaluable paired biopsies (*n* = 19) indicated a nonstatistically significant trend of an increase in mature DC gene set enrichment scores in biopsies after treatment from the MP0317-higher dose group (*n* = 14; Fig. 4d). Additional trends of increased enrichment score for gene signatures associated with plasma and follicular helper T (T_fh_) cells, as well as interferon-γ (IFNγ) pathway activation, were observed in the MP0317-higher dose group. The lack of statistical significance of these gene expression readouts could potentially be explained by the relatively small sample size (30% of samples were unevaluable) and the heterogeneity of the patient population. Changes in gene signatures related to all major immune cell populations in the TME for the MP0317-higher dose group are shown in Extended Data Fig. 2.

**Fig. 5:**
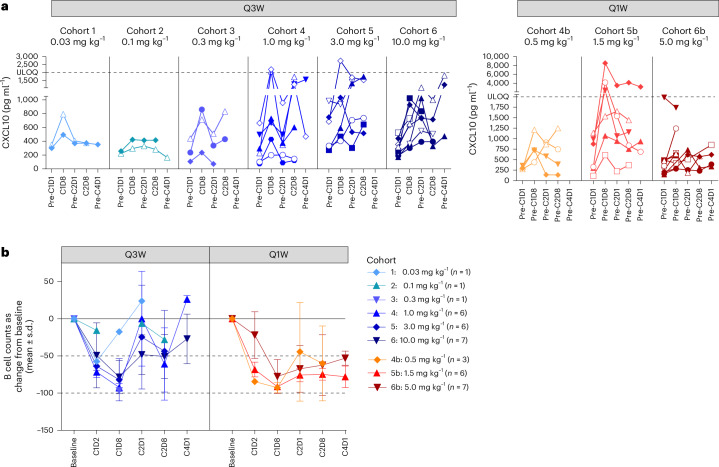
Pharmacodynamic effects of MP0317 in the periphery (blood). **a**, CXCL10 serum levels (pg ml^−1^) after MP0317 treatment are shown per cohort (left: Q3W cohorts 1–6; right: Q1W cohorts 4b–6b). All patients showed transient CXCL10 increases, with larger responses at pharmacologically active doses (≥0.3 mg kg^−1^). The dashed line indicates the assay’s upper limit of quantification (ULOQ) (2,000 pg ml^−1^). **b**, Peripheral B cell abundance after MP0317 treatment shown as absolute B cell counts (cells per µl), measured using flow cytometry (gated on CD3^−^CD19^+^ cells) and presented as the mean percentage change from baseline per cohort (Q3W, cohorts 1–6; Q1W, cohorts 4b–6b) ± s.d. The baseline was the C1D1 pre-dose. The analysis included longitudinal evaluable samples from 38 patients. Source data

**Extended Data Fig. 3:**
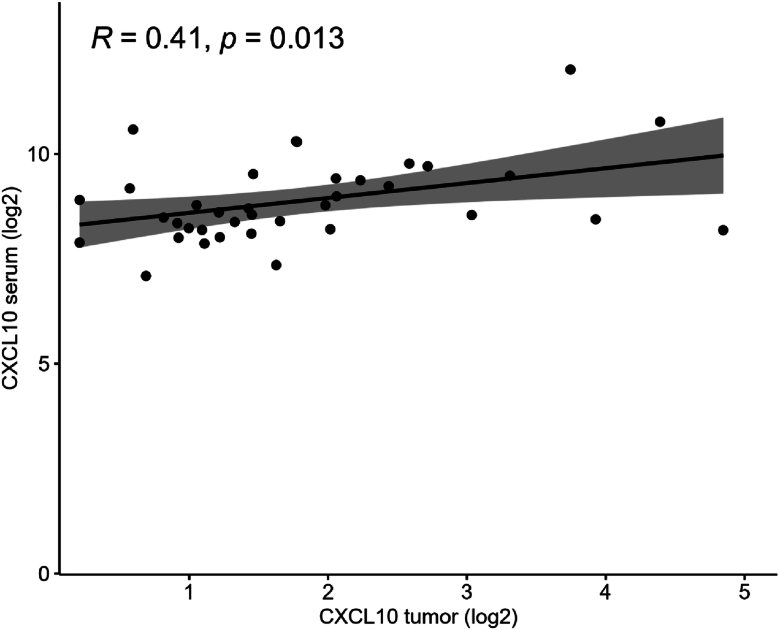
Correlation between serum CXCL10 and CXCL10 gene expression in tumor. Correlation analysis between CXCL10 in the serum and CXCL10 mRNA expression levels in the tumor at baseline and Cycle 2 Day 8. Spearman’s rank correlation analysis (using a two-sided test) of n = 19 patient samples (18 paired baseline and Cycle 2 Day 8, 1 unpaired at baseline). The black line shows a linear regression fitted to the data, and the gray bands show the 95% confidence interval for this regression. RNA was measured in transcript per million (TPM) and serum cytokines in pg/mL. Source data

Elevated serum levels of the chemoattractant marker CXCL10 after MP0317 treatment were consistently observed in all patients (*n* = 46; Fig. 5a). The observed increases were more pronounced in patients from the MP0317-higher dose group. Serum CXCL10 concentrations were significantly correlated (*P* = 0.045) with tumor *CXCL10* gene expression, suggesting that cells within the TME that have been modulated by MP0317 may be the source of this circulating factor (Extended Data Fig. 3).

**Extended Data Fig. 4:**
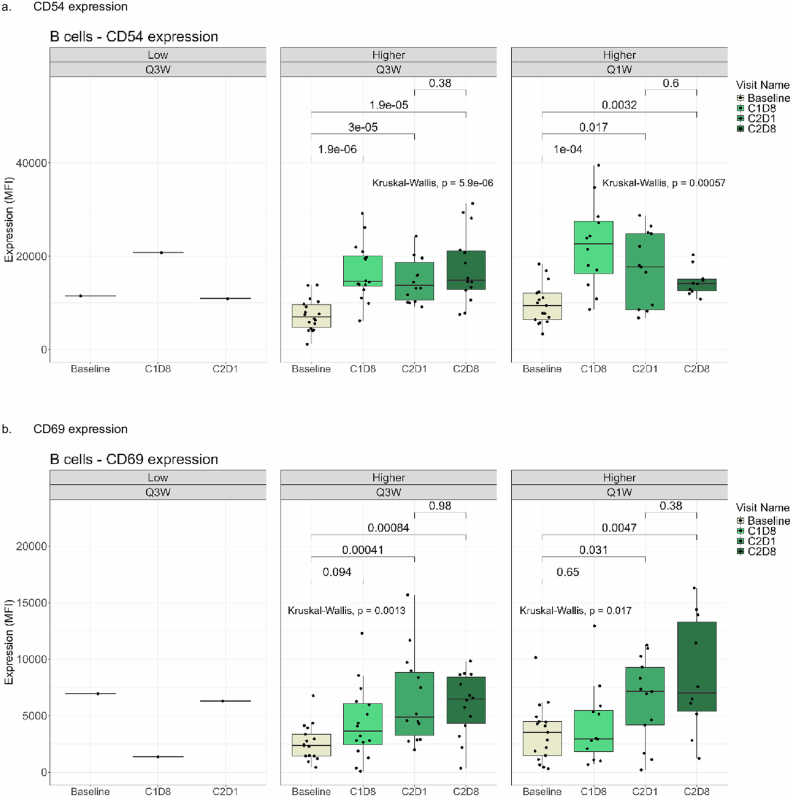
CD54 and CD69 expression on B cells after MP0317 treatment. Median Fluorescence Intensity (MFI) expression levels of a. CD54 and b. CD69 on B cells in fresh whole blood by flow cytometry at the indicated timepoints (Q3W: Cycle 1 Day 1 and Cycle 2 Day 1 sample collection was done pre dose; Q1W: sample collection at all timepoints was done pre dose). B cells were gated as CD45 + CD3–CD19+ from live cells. Left panel (low doses) includes patients treated with MP0317 doses ≤0.1 mg/kg administered Q3W. Middle and right panels (higher doses) include patients treated with MP0317 doses ≥0.3 mg/kg administered Q3W or Q1W, respectively. Each dot indicates a value. Analysis was done on longitudinal evaluable samples of n = 43 patients (low doses Q3W n = 2; higher doses Q3W n = 22; higher doses Q1W n = 19). The upper (75%), median, and lower (25%) percentiles are indicated in the boxplots. The upper whisker extends to the largest value no further than 1.5 * IQR (inter-quartile range) from the boxplot hinge. The lower whisker extends to the smallest value at most 1.5 * IQR of the boxplot hinge. A two-sided Kruskal-Wallis test by ranks (non-parametric) was used to calculate differences in distribution among the groups. Further two-sided Wilcoxon tests were used to compare the difference in the sum of ranks between timepoints. Source data

**Extended Data Fig. 5:**
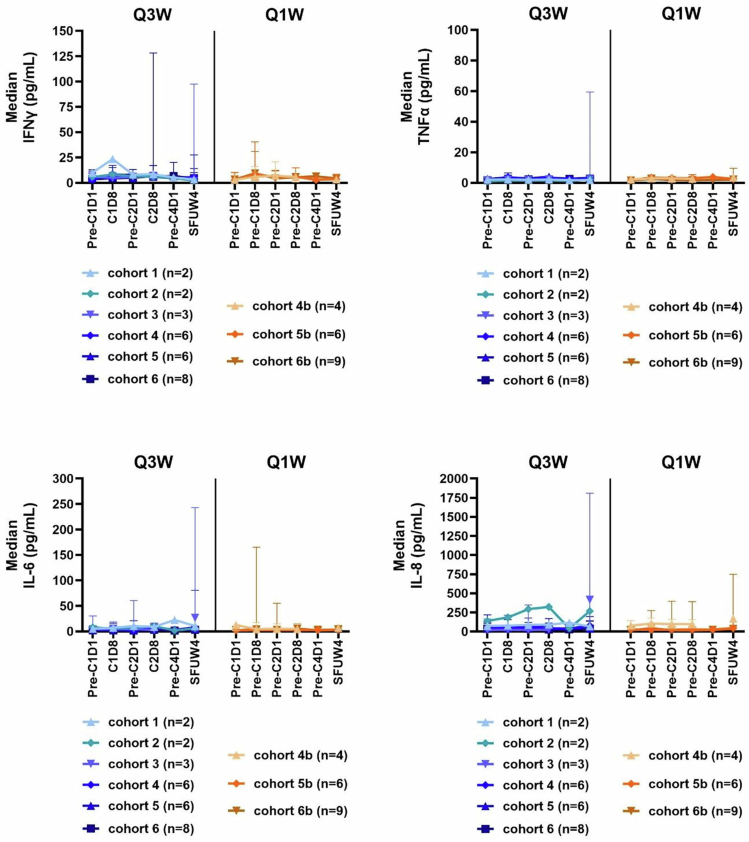
Proinflammatory cytokine induction following MP0317 treatment. Median serum levels (pg/ml) +/- SD of IFNγ, TNFα, IL-6 and IL-8 cytokines per cohort (left panel Q3W, Cohorts 1–6 and right panel Q1W, Cohorts 4b–6b) at the indicated timepoints. Source data

Blood immunophenotyping indicated a transient reduction in peripheral B cell abundance 1 week after dosing in the MP0317-higher dose group (Fig. 5b). This was accompanied by a significant (*P* < 0.05) and sustained upregulation of the activation markers CD54 and CD69 on peripheral B cells (*n* = 43; Extended Data Fig. 4). The increased plasma cell gene signature enrichment score in tumors after MP0317 treatment, described above, suggests that tumor homing may potentially explain this reduction in peripheral B cell abundance. The abundance of peripheral T cells, natural killer (NK) cells and monocytes was not significantly altered by MP0317 treatment. In keeping with the acceptable safety profile of MP0317 described above, no pronounced induction of proinflammatory cytokines (for example, TNF, interleukin-6) was observed in the serum (Extended Data Fig. 5).

### Recommended phase 2 dose

Based on the totality of the data, including tolerability, clinical efficacy, PK and PD response, the recommended dose for further exploration of MP0317 is at doses higher or equal to 1.5 mg kg^−1^ Q1W and 3 mg kg^−1^ Q3W, with adjustable dosing frequency to match a combination dosing scheme.

## Discussion

Several anti-CD40 agonistic antibodies have been evaluated as monotherapy in clinical trials in oncology. However, most of these did not progress beyond the early clinical trial phases, particularly because of toxicity, including liver toxicity, cytokine release syndrome, IRRs and thrombocytopenia, and limited antitumor activity in monotherapy^4,27–29^. To circumvent the systemic toxicities, intratumoral injection was implemented^30,31^ and further efforts were made to enable CD40 agonism locally at the tumor site, either in a tumor-specific approach, for example, by using bispecific antibodies against a tumor-associated antigen^32^, such as mesothelin^33^ or carcinoembryonic antigen^34^, or tumor-agnostic targeting of the TME^35^. MP0317, a FAP x CD40 DARPin, uses this approach by activation of CD40 dependent on cross-linking of FAP. FAP is a cell-surface serine protease overexpressed on stromal fibroblasts within various solid tumors^23,24^, as evidenced by the broad and robust uptake of FAP inhibitor tracers in positron emission tomography imaging across a wide range of tumor types, highlighting its potential as a universal target in oncology^23,36^. Its expression in healthy tissues is limited, mainly to tissues undergoing physiological remodeling, such as during wound healing.

In this first-in-human phase 1 study, we investigated the safety, PK and PD, and preliminary clinical activity of MP0317 administered intravenously Q3W or Q1W in doses of 0.03–10 mg kg^−1^ in 46 adults with advanced solid tumors. Our findings showed a favorable safety profile for MP0317 across all nine dose-escalation cohorts. Most TRAEs were mild (grades 1 and 2), and the MTD was not reached. This compares favorably with the safety profile of several systemic anti-CD40 agonistic antibodies, which were confronted with DLTs in their early development^8^. For example, for mitazalimab and sotigalimab, dose-limiting hepatotoxicity was reported in clinical trials (drug-induced liver injury^37^, liver enzyme elevations^38^). Cardiac toxicity did not stand out in clinical trials with systemic CD40 agonists^8^. However, preclinical cardiac injury models showed that anti-CD40 agonist antibody exposure could indeed elicit cardiac inflammation by triggering a feed-forward inflammatory loop between cardiac CCR2^+^ macrophages and CD8^+^ T cells, which sensitizes the heart to subsequent injury resulting in accelerated left ventricular remodeling, identifying a possible cardiovascular risk of CD40 agonists^39^. Targeting the CD40 agonism to the tumor site using FAP, which is not expressed in healthy tissues, should circumvent these systemic toxicities. Still, grade 1 asymptomatic heart failure was observed in one patient in the trial. It cannot be excluded that because of previous cardiotoxic chemotherapy and radiotherapy, there was pre-existing cardiac damage and fibrosis in this patient (New York Heart Association stage II at baseline), with cardiac upregulation of FAP^40,41^. On-target, off-tumor activation of CD40 with resulting cardiac inflammation could have contributed to the cardiac toxicity observed.

Overall, the results reported confirm that tumor-localized CD40 activation in the TME is a safe approach and can abrogate the systemic toxicity observed with non-targeted CD40 agonists^35^.

The ORR and DCR were 2% and 33%, respectively, with one patient with GIST achieving an unconfirmed PR and SD observed in 14 additional patients. Whereas in preclinical models, FAP-targeted CD40 agonism induced robust antitumor efficacy in specific models^18^, the clinical efficacy with MP0317 as a single therapy was limited, which we speculate might be attributed to the advanced disease stage of the patient population and the compromised state of their immune system, suggesting that earlier lines of treatment or a neoadjuvant setting could be more advantageous. Additionally, the lack of response might reflect the complexity of tumor antigen presentation and variability in intrinsic antitumor responses among the trial population. Although tumors arise from pre-existing tissues, generally malignancies can be recognized as ‘non-self’ by the body’s immune system. Diverse parameters can influence the adaptive antitumor response, including tumor mutational burden, host HLA repertoire, tumor heterogeneity, PD-L1 expression and microbiome composition^42^.

Consistent with our earlier studies in animal models^18^, the biomarker analyses of paired tumor biopsies confirmed the desired tumor localization and activation of the CD40 pathway by MP0317. The TME was remodeled upon MP0317 treatment, with increases in DCs, plasma cells and T_fh_ cells. There was evidence of IFNγ pathway activation and an increased DC maturation gene signature score. DCs and T_fh_ cells have been described to produce IFNγ^43,44^ and may potentially explain the induction of the IFNγ downstream signature by MP0317. Alternatively, as this signature includes several genes related to antigen presentation (for example, *HLA-DRA*)^45^, its induction after MP0317 treatment may also reflect the activation of APCs. Peripheral PD effects, like increases in CXCL10 chemoattractant and transient activation, and reduced abundance of peripheral B cells, were also in line with the desired mode of action of MP0317. We did not observe overt changes in cytotoxic T lymphocyte infiltration and activation, which suggests that CD40 activation and TME modulation alone is not sufficient to induce T-cell-mediated antitumor immunity, and needs to be combined with complementary approaches (for example, T cell activation)^46^.

It is tempting to speculate that the increased intratumoral DC and plasma cell infiltration and activation is indicative of tertiary lymphoid structure (TLS) formation. TLS are ectopic lymphoid organs composed of immune (T cells, DCs and B cells) and nonimmune cells (high endothelial venules and stromal cells) that have been recently proposed as privileged sites for immune cell infiltration, tumor antigen presentation and activation and proliferation of CD8^+^ T and B cells^42,47–49^. Several studies have associated the presence of TLS with better prognosis across various malignancies^50,51^, and with improved responses to immunotherapies^52,53^. However, there is limited data on the precise pathways that mediate de novo induction of TLS by immunotherapies^54,55^. It will be of considerable interest in future preclinical, mechanistic, and clinical studies, to examine whether tumor-targeted CD40 activation with MP0317 induces TLS formation, and whether such effects deliver increased response to this agent in combination with other immunotherapies or vaccines.

The main limitations of this study are its small sample size, nonrandomized design and focus on heavily pretreated patients with an advanced disease, which may underestimate potential efficacy in earlier settings. The trial was not powered to assess clinical benefit, so conclusions about antitumor activity remain preliminary. Additionally, biomarker findings, while consistent with CD40 pathway activation, were exploratory, and in some cases, not statistically powered because of limited evaluable samples. Therefore, this research cannot yet establish the definitive clinical efficacy of MP0317 or predict outcomes in combination regimens—those questions require larger, controlled studies.

In conclusion, the encouraging safety profile and profound TME remodeling observed in this study are consistent with the intended mechanism of tumor-localized CD40 agonism by MP0317. The consistent activation of the CD40 pathway in a heterogeneous patient population, coupled with the FAP expression in a broad range of tumor types, suggests that MP0317 could have application across diverse solid tumor settings. Combination with T-cell-targeted therapeutic modalities (for example CPIs, chimeric antigen receptor/CAR T cells, T cell engagers) could represent an attractive complementary approach to leverage the tumor remodeling mechanism of MP0317 to improve patient responses. Preclinically, the combination of CD40 activation with immune checkpoint blockade induced the generation of polyfunctional T cells and delivered robust antitumor efficacy in a therapy-refractory pancreatic cancer model^56^. To date, the small number of later-stage clinical studies examining this combination concept have been limited to nontumor-targeted CD40 agonists. Our study with MP0317 indicated that tumor-targeted CD40 activation is amenable to flexible dosing regimens and is associated with a relatively low propensity for systemic immune-related toxicities, which further support the investigation of MP0317 in combination with such complementary therapies in future studies.

## Methods

### Study design, patient eligibility and treatment

This was a phase 1, first-in-human, open-label, nonrandomized, dose-escalation study designed to assess the safety, tolerability, PK and PD profile of MP0317 (NCT05098405). The dose-escalation part was designed to determine the recommended dose for expansion (RDE) or the MTD for MP0317 monotherapy, while the safety expansion part was designed to confirm safety in a larger population. The dose-escalation scheme used an adaptive study design following a Bayesian logistic regression model (BLRM). The clinical study protocol was approved by the independent ethics committees Sud-Ouest et Outre-Mer II and The Medical Research Ethics Committee NedMec. The study was conducted in accordance with the ethical principles in the Declaration of Helsinki. All 61 participants provided written informed consent. A dose-escalation review committee monitored safety and governed all cohort dosing decisions. The safety expansion part was ultimately not conducted because the safety profile of MP0317 in monotherapy was considered adequately characterized. The study was conducted at two sites in France (Centre Léon Bérard in Lyon and IUCT-Oncopole in Toulouse) and the Netherlands (The Netherlands Cancer Institute in Amsterdam and the Department of Medical Oncology UMC Utrecht). Adult patients were eligible for inclusion if they had an ECOG PS of 0 or 1, and a life expectancy of 12 or more weeks per investigator judgment. Patients were not required to be FAP^+^ but needed to have measurable disease per RECIST v.1.1 from an advanced, histologically proven solid tumor of one of the protocol-specified types, based on reported FAP expression^19,23^, and for which approved therapies had been exhausted or for which the investigator considered the patient ineligible or unable to tolerate other treatments: colorectal cancer, ovarian cancer, endometrial cancer, gastric cancer, pancreatic cancer, anal cancer, cervical cancer, head and neck squamous cell carcinoma, mesothelioma, prostate cancer, NSCLC, melanoma, urothelial or bladder cancer, microsatellite-instability-high cancer of any type, cutaneous squamous cell cancer or breast cancer. Exceptionally, patients with other tumor types could be included after discussion with the sponsor based on reported FAP expression and potential benefit from immune therapy. A washout period of 21 days before first study drug administration was requested for prior anticancer treatment, including chemotherapy, hormonal therapy or radiotherapy of 28 days for prior investigational treatment. Mandatory paired tumor biopsies were collected at baseline and on treatment to assess local activation of CD40 within the TME as per the proposed mode of action.

MP0317 was administered intravenously in nine dose-ascending cohorts. Initially a Q3W regimen with up to six dose levels was planned. These dose levels were selected based on a translational PK/PD model linking MP0317 serum exposure levels and the predicted tumor CD40 target occupancy time course, based on potency estimates from an in vitro co-culture assay using human B cells and FAP-transfected CHO cells^18^. Three additional Q1W dose levels were introduced with a study amendment based on emerging clinical PK data from systemic CD40 agonists, which were not accounted for in the PK and PD model, but suggested a stronger CD40-mediated antigen sink and higher TMDD than predicted^37^. The Q1W regimen aimed to achieve both a higher extent and a longer duration of CD40 activation within the TME throughout the dosing interval. Patients were treated in 21-day cycles until disease progression, unacceptable toxicity, withdrawal of consent or other reasons to discontinue treatment, whichever occurred first.

The study protocol is included in Supplementary File 1.

### Endpoints and assessments

The primary endpoints of the study were safety and tolerability, and identification of the MTD and RDE. Data on AEs and SAEs were collected from signing the informed consent form until 28 days after the last study drug administration or patient end of study. Secondary endpoints were the PK profile and antitumor activity of MP0317. Assessments for the latter included ORR and DCR according to RECIST v.1.1 and iRECIST (modified RECIST in cancer immunotherapy studies), as well as PFS. A patient’s disease was considered controlled at predefined time points, if the response evaluation resulted in a CR/iCR, PR/iPR or SD/iSD. The DCR was evaluated at first TA during treatment (at approximately 4–6 weeks), and on study days 90, 180 and 270. Exploratory endpoints assessed the PK and immunogenicity effects of MP0317.

Safety parameters were reviewed by the dose-escalation review committee before each dose-escalation. The DLT evaluation period was 28 days. Tumor response was monitored using computed tomography scans every 6–8 weeks with evaluation per RECIST v.1.1 and iRECIST.

### Bioanalytical assays

MP0317 concentrations in serum were assessed using an electrochemiluminescence immunoassay developed and validated at Molecular Partners, which uses as capture reagent biotinylated human recombinant CD40 (Acrobiosystems) and as detection reagent a Sulfotag monoclonal antibody anti-MP0317 (CePower). The limit of quantification of the PK assay is 5 ng ml^−1^.

Detection of ADAs against MP0317 was performed with a validated method developed at Molecular Partners (electrochemiluminescence-based assay), using as a positive control the humanized anti-DARPin monoclonal antibody (Evitria).

### PK data analysis

The following PK parameters were calculated from the serum MP0317 PK raw data: *C*_max_, time to *C*_max_ (*t*_max_), minimum serum concentration (*C*_min_), AUC, total clearance, volume of distribution at steady state (*V*_ss_) and half-life (*t*_1/2_).

Specifically, for the Q3W dosing cohorts (cohorts 1–6 with intravenous administration at 0.03, 0.10, 0.30, 1.0, 3.0 and 10 mg kg^−1^) both cycles 1 and 3 PK data were used to perform noncompartmental analysis, while for the Q1W dosing cohorts (cohorts 4b–6b with intravenous administration at 0.5, 1.5 and 5 mg kg^−1^) cycle 1 day 1 and cycle 1 day 15 were used instead.

PK parameters were calculated using standard noncompartmental PK analysis using the software Phoenix WinNonlin (v.8.4 or higher). ADA integrated summary was also performed using Phoenix.

For the PK analysis, the PK analysis set was used.

For PK parameter determination using noncompartmental analysis, the following rules were used for the PK data below the limit of quantification (BLQ) of the PK assay:

- Pre-dose BLQ values (before the first study dose in cycle 1 day 1) were set to ‘0’.
- Pre-dose BLQ values in subsequent visits after cycle 1 day 1 were set to the lower limit of quantification (0.005 μg ml^−1^) (LLOQ)/2.
- For all visits, the first post-dose BLQ value was set to LLOQ/2, whereas subsequent BLQ values were treated as missing.
- Embedded BLQ values (between two measurable concentrations) were treated as missing.

### Immunogenicity analysis

Results of ADAs against MP0317 were integrated based on results of the ADA assay to determine the immunogenicity potential of MP0317. The immunogenicity analysis set was used to integrate the ADA summary. To integrate the immunogenicity results, the incidence rate of ADA was derived. The number of ADA^+^ patients (including treatment-induced and treatment-boosted as subcategories) and ADA^−^ patients (including ADA^−^ and treatment-unaffected as subcategories) throughout the study was reported for each cohort, for each dosing frequency (Q1W or Q3W) and overall according to the terminology by Shankar et al.^57^. The incidence rate of ADAs was reported as the number of patients in each category divided by the number of patients in the cohort.

The onset of ADA appearance was defined as the time point where the first ADA^+^ sample was observed. The onset of the ADA response was summarized based on basic statistics (median, range) for each cohort, according to dosing frequency and overall. Finally, the association of ADA and PK was evaluated to determine whether ADAs were clearing in nature.

### PD (biomarker analyses)

Evaluable paired (*n* = 26) pre-treatment and on-treatment tumor biopsies taken at cycle 2 day 8 (that is, 28 days after the first dose of MP0317) were analyzed with mIF and bulk RNA-seq at a central laboratory. Peripheral serum and fresh blood collected as per protocol schedule of the assessments were analyzed using enzyme-linked immunosorbent assay and Meso Scale Discovery immunoassay and flow cytometry, respectively, at a central laboratory.

### mIF analyses

A customized method for detecting eight biomarkers (FAP, CD40, a-DARPin, CD3, CD68, CD11c, CD163 and Pan cytokeratin) via mIF on formalin-fixed paraffin-embedded tumor tissue samples was qualified by Precision for Medicine. For each patient sample, hematoxylin and eosin stain was performed on one tissue section slide, then digitalized using the PhenoImager HT system (Akoya, formerly Vectra Polaris); tissue quality was evaluated and annotated by a pathologist. Tumor biopsy annotations included tumor, non-tumor and eventual areas of exclusions (such as necrosis, hemorrhage). Slides were loaded onto the Leica BOND Rx automatic staining instrument and stained with a 9-color panel with the primary antibodies for each biomarker; 4’,6-diamidino-2-phenylindole staining was used to determine the number of nuclei and morphology. Once stained and cover-slipped, slides were scanned using the PhenoImager HT system, unmixed using the inForm software and analyzed using the HALO image analysis software. The total percentage of nucleated cells positive for a specific biomarker out of the total nucleated cells, and cell density (count per μm^2^), was scored in two regions per sample. The antibodies used for mIF are provided in Supplementary Tables.

### RNA-seq analysis

RNA-seq of biopsies was performed using an Illumina Novaseq 6000 by Neogenomics. Sequencing results were converted using bcl2fastq then aligned using STAR^58^; normalized transcripts per million were computed using TPMCalculator^59^. For RNA-seq and alignment quality control, the following measures were evaluated: gene origin of reads; per sequence quality score; per base sequence content; per sequence GC content; sequence length distribution; and sequence duplication levels. Gene set enrichment scores were then computed using the GSVA R package. The gene signatures^45,60,61^ used are provided in Supplementary Tables. Analysis was done on prespecified groups as for the mIF data. Statistical analysis of changes in GSVA scores was performed using a paired Wilcoxon signed-rank test, using the ggpubr package.

### Flow cytometry

Whole-blood samples in 4 ml sodium heparin tubes were sent at ambient temperature for sample preparation and flow cytometry analysis according to validated assay protocols for research use. A multi-color validated custom flow cytometry panel, designed to identify and enumerate the T, B and NK cell subsets was used for monitoring blood cell numbers (Q2 Solutions). Samples were stained with primary antibodies for CD3, CD4, CD8, CD14, CD16, CD19, CD45 and CD56, and analyzed on BD FACS Canto II flow cytometers (BD Biosciences). Lymphocytes were gated on CD45^+^ side scatter (SSC), excluding debris and monocytes. T cells (CD3^+^), B cells (CD19+) and NK cells (CD3^−^CD16^+^CD56^+^) were identified within the lymphocyte gate, with CD4/CD8 defining T cell subsets. A multi-color flow cytometry panel was designed to identify and enumerate the immune cell subsets of interest (that is B, T and DC cells) described below. Samples were acquired using the Cytek Aurora (Cytek Biosciences). The combination of fixable viability stain and SSC-A was used to exclude dead cells from the analysis and CD45 expression to determine the CD45^+^ leukocyte population. Expression of CD19, HLA-DR and CD14 was used to define monocytes and B cells, with CD56, CD16 and CD3 used to define NK, NKT and T cells, respectively. CD8 and CD4 were then used to further define cytotoxic and helper T cells, respectively. DC subsets were defined by CD123 and CD11c expression and further classified by the expression of CD1c and CD141. CD86, CD69, CD54, CD40, CD25 and HLA-DR markers were assessed in the previously defined subsets. Data analysis was performed with the BD FACSDiva or SpectroFlo software and ggplot2 and ggpubr packages in R v.4.5.

The flow cytometry panels and antibodies used for immunophenotyping are provided in Supplementary Tables.

### Electrochemiluminescence assays

Serum aliquots of clinical samples (up to 0.5 ml per vial) were stored at −80 °C till the analyses below were performed by Precision for Medicine. The concentration (pg ml^−1^) of IFNγ, interleukin-10 (IL-10), IL-12p70, IL-13, IL-1β, IL-2, IL-4, IL-6, IL-8, TNF, IP-10 (CXCL10), MIG (CXCL9) in serum was measured using a validated electrochemiluminescence (ECL) method (MSD). The following ready-to-use kits were used: V-PLEX Plus Proinflammatory Panel 1 (human) Kit (cat. no. K150496-2), V-PLEX Plus Human IP10 Kit (cat. no. K151NVG), R-PLEX Human MIG Assay, SECTOR (cat. no. K1510IR) with Antibody Set (cat. no. B210I-3). The V-PLEX Plus kits are developed and validated by the manufacturer, which provides lot no. specifications, including LLOQ and ULOQ reference values. Analyses were performed according to the manufacturer’s instructions. The R-PLEX Human MIG Assay is an early developed assay within the MSD platform and was further qualified by Precision for Medicine.

sFAP and sCD40 concentrations were determined in human serum using ECL-based sandwich immunoassays, qualified at Molecular Partners. To analyze sFAP, an MSD GOLD 96-well Streptavidin SECTOR Plate (cat no. L15SA, Meso Scale Discovery) was coated with biotinylated human FAP antibody (cat. no. DY3715, R&D systems). The reference material used for calibration standards and the quality control preparation was recombinant human FAP (cat. no. 3715-SE, R&D Systems). SulfoTag-labeled D8 antibody (cat. no. MABS1001, Vitatex) was used for detection. To analyze sCD40, an MSD GOLD 96-well Small Spot Streptavidin SECTOR Plate (cat. no. L45SA, Meso Scale Discovery) was coated with biotinylated human CD40 antibody (cat. no. C217B-3, Meso Scale Discovery). The reference material used for the calibration standards and quality control preparation was recombinant human CD40 (cat. no. C017B-2, Meso Scale Discovery). SulfoTag-labeled human CD40 antibody (cat. no. D217B-3, Meso Scale Discovery) was used for detection.

### Statistics and reproducibility

Nine dose levels were tested with at least two to nine patients per dose level. The following analysis sets were defined in the statistical analysis plan:

- The safety analysis set (SAS) included all patients who received at least one administration of MP0317 and had at least one post-dose safety assessment. The SAS was the primary population for all demography, safety, immunogenicity, efficacy and PD related endpoints, except for determination of the dose–DLT relationship.
- The dose-determining set (DDS) included all patients in the SAS who met the DLT evaluability criteria. The DDS was used to estimate the dose–DLT relationship in the dose-escalation part of the study.
- The PK analysis set consisted of all patients who received at least one dose of MP0317 and had at least one post-dose PK measurement.
- The immunogenicity analysis set consisted of all patients in the SAS who had at least one post-baseline blood sample collected to assess immunogenicity.

For the dose-escalation part, a two-parameter adaptive BLRM was used to determine the RDE or MTD. The BLRM was guided by the escalation with overdose control principle which mandated that any dose of MP0317 that had more than a 25% chance of being in the excessive toxicity category was not considered for the next cohort.

AEs were coded using the MedDRA v.24.1 and updates. Safety laboratory assessments were performed in a local laboratory and analyzed according to patient and visit. Vital signs and pulse oximetry, electrocardiogram, physical examination and ECOG PS were analyzed according to treatment group and visit.

Efficacy analyses were done according to investigator‑assessed response criteria: ORR was summarized with two-sided 90% exact Clopper–Pearson CIs for binomial proportions. In addition, waterfall plots were created displaying the maximum percentage change in sum of the longest diameter of the target lesions compared to baseline and the best overall response according to RECIST v.1.1/iRECIST. The DCR was summarized with two-sided 90% exact Clopper–Pearson CIs for binomial proportions according to treatment group.

For all response assessments, swimmer plots were created displaying the RECIST v.1.1/iRECIST assessments per patient over time.

Patient demographics and other screening data were listed and summarized using descriptive statistics. Exposure to study treatment was summarized with descriptive statistics for the total number of infusions received.

Data distribution was assumed to be normal but this was not formally tested.

### Reporting summary

Further information on research design is available in the Nature Portfolio Reporting Summary linked to this article.

## Supplementary information

Reporting Summary

Supplementary File 1 Redacted study protocol.

Supplementary File 2 CONSORT checklist.

Supplementary Tables Supplementary Table 1: Summary statistics of serum MP0317 PK parameters following first MP0317 IV infusion. Statistics of serum MP0317 PK parameters following first MP0317 IV infusion (Cycle 1 Day 1) in cohorts 1–6 (a.) and cohorts 4b, 5b and 6b (b.). Data presented as arithmetic mean (CV%); n (number of patients) except for t_max_ and t_last_ which are presented as median (min-max); n (number of patients). Parameters are calculated relative to the start of infusion. NC= not calculated. Supplementary Table 2: Incidence and frequency of anti-drug antibodies (ADAs) by MP0317 dose cohort (Q3W and Q1W). ADA status was assessed based on the following categories according to ref. ^57^: ADA-negative samples: Patients who had no pre-existing ADAs or were missing ADA data before drug administration and who had all negative ADA results following drug administration. Treatment-unaffected ADA: Subset of ADA negative patients who had pre-existing ADAs but did not show a ≥4-fold increase in ADA titer following drug administration compared to baseline measurement. Treatment-induced: ADAs developed de novo (seroconversion) following drug administration. Patients who had no pre-existing ADAs or were missing ADA data before drug administration and who had at least one ADA^+^ sample following drug administration. Treatment-boosted: Patients who had pre-existing ADAs and had a ≥4-fold increase in ADA titer following drug administration compared to baseline measurement.

## Source data

Source Data Figs. 2–5 and Extended Data Figs. 2–5 Statistical source data.

## Data Availability

The RNA-seq data are available at GSE287512. Data that support the study findings are available to researchers upon reasonable request to the corresponding author, if in alignment with study consent and in non-identifiable format to protect patient privacy. Source data are provided with this paper.
