# Supplementary material for: Tumor-localized CD40 agonism with MP0317, a FAP x CD40 DARPin, reprograms the tumor microenvironment in patients with advanced solid tumors: an open-label, nonrandomized, dose-escalation phase 1 study
Source: Nat Cancer. 2026 May 1;7(5):810–22. doi: 10.1038/s43018-026-01150-1 (PMC13221297; doi:10.1038/s43018-026-01150-1)
Supplement: Supplementary file 1 — Reporting Summary [file 43018_2026_1150_MOESM1_ESM.pdf]

nature portfolio

Corresponding author(s): Hilde De Winter

Last updated by author(s): 02/12/2025

Reporting Summary

Nature Portfolio wishes to improve the reproducibility of the work that we publish. This form provides structure for consistency and transparency in reporting. For further information on Nature Portfolio policies, see our Editorial Policies and the Editorial Policy Checklist.

Statistics

For all statistical analyses, confirm that the following items are present in the figure legend, table legend, main text, or Methods section.

n/a	Confirmed	
☐	☒	The exact sample size (*n*) for each experimental group/condition, given as a discrete number and unit of measurement
☐	☒	A statement on whether measurements were taken from distinct samples or whether the same sample was measured repeatedly
☐	☒	The statistical test(s) used AND whether they are one- or two-sided<br>*Only common tests should be described solely by name; describe more complex techniques in the Methods section.*
☐	☒	A description of all covariates tested
☐	☒	A description of any assumptions or corrections, such as tests of normality and adjustment for multiple comparisons
☐	☒	A full description of the statistical parameters including central tendency (e.g. means) or other basic estimates (e.g. regression coefficient) AND variation (e.g. standard deviation) or associated estimates of uncertainty (e.g. confidence intervals)
☐	☒	For null hypothesis testing, the test statistic (e.g. *F*, *t*, *r*) with confidence intervals, effect sizes, degrees of freedom and *P* value noted<br>*Give P values as exact values whenever suitable.*
☐	☒	For Bayesian analysis, information on the choice of priors and Markov chain Monte Carlo settings
☒	☐	For hierarchical and complex designs, identification of the appropriate level for tests and full reporting of outcomes
☐	☒	Estimates of effect sizes (e.g. Cohen's *d*, Pearson's *r*), indicating how they were calculated

Our web collection on statistics for biologists contains articles on many of the points above.

Software and code

Policy information about availability of computer code

Data collection	Clinical data was collected using the EDC system Clincase. AEs were coded using MedDRA v24.1 and updates.
Data analysis	-PK parameters were calculated with standard non-compartmental PK analysis using the software Phoenix® WinNonlin® (Version 8.4 or higher).<br>-ADA integrated summary was also performed using Phoenix®.<br>-Tumor biopsy immunofluorescence slides were scanned using PhenoImager HT system (Akoya; formerly Vectra Polaris), unmixed using inForm software and analyzed using the HALO image analysis software.<br>-RNA sequencing of the biopsies was performed using an Illumina Novaseq 6000 by Neogenomics (CA, USA). Sequencing results were converted using bcl2fastq then aligned using STAR and normalized transcripts per million (TPM) were computed using TPMCalculator. Gene set enrichment scores were then computed using the gene set variation analysis (GSVA) R package.<br>-Flowcytometry analysis was performed with BD FACSDiva™ or SpectroFlo™ software tools and ggplot2 and ggpubr packages in R, version 4.5.

For manuscripts utilizing custom algorithms or software that are central to the research but not yet described in published literature, software must be made available to editors and reviewers. We strongly encourage code deposition in a community repository (e.g. GitHub). See the Nature Portfolio guidelines for submitting code & software for further information.

Data

Policy information about availability of data

All manuscripts must include a data availability statement. This statement should provide the following information, where applicable:

- Accession codes, unique identifiers, or web links for publicly available datasets
- A description of any restrictions on data availability
- For clinical datasets or third party data, please ensure that the statement adheres to our policy

RNA seq data is publicly available at GSE287512. Data that support the study findings are available to researchers upon reasonable request to the corresponding author, if in alignment with study consent and in not identifiable format to protect patient privacy.

Research involving human participants, their data, or biological material

Policy information about studies with human participants or human data. See also policy information about sex, gender (identity/presentation), and sexual orientation and race, ethnicity and racism.

Reporting on sex and gender	This clinical study was open to all eligible patients independent of their sex. Accordingly, 24 females and 22 males were treated in the study. The outcome analysis was carried out for the entire patient population and was not sex specific. The primary objective was to establish safety, and the sample size was inadequate for sex-specific subgroup analyses.
Reporting on race, ethnicity, or other socially relevant groupings	Data on race, ethnicity or other socially relevant characteristics was not collected within this study, and hence are not reported in the manuscript.
Population characteristics	This study was open to adult patients who met the predefined study eligibility criteria. The age range of trial participants was 35 to 79 years (median 63 years). All patients had a diagnosis of an advanced solid tumor (for more information on patient demographics please see Table 1 of the manuscript).
Recruitment	Potential study candidates were recruited from the oncology clinical practices at the 4 participating sites. Potentially eligible patients were informed about the study. Sixty-one patients interested in participating in the study provided written informed consent and proceeded to scrrening. Of these, 46 met all eligibility criteria and were treated in the study.
Ethics oversight	This phase 1 clinical study protocol and its amendment were approved by the independent ethics committees Sud-Ouest et Outre-Mer II and The Medical Research Ethics Committee NedMec. The study was conducted in accordance with the ethical principles in the Declaration of Helsinki. All 61 participants provided written informed consent.

Note that full information on the approval of the study protocol must also be provided in the manuscript.

Field-specific reporting

Please select the one below that is the best fit for your research. If you are not sure, read the appropriate sections before making your selection.

☒ Life sciences ☐ Behavioural & social sciences ☐ Ecological, evolutionary & environmental sciences

For a reference copy of the document with all sections, see nature.com/documents/nr-reporting-summary-flat.pdf

Life sciences study design

All studies must disclose on these points even when the disclosure is negative.

Sample size	The actual number of 46 treated patients was derived from the number of dose levels/cohorts that was evaluated (9 in total), the number of patients considered non-DLT-evaluable who were replaced, and he safety profile observed at each dose level. There was no sample size calculation or powering.
Data exclusions	No data were excluded from the analysis. All relevant clinical and laboratory data are reported.
Replication	Not applicable for this phase 1 clinical trial.
Randomization	Not applicable, as this was an open-label, non-randomized, dose-escalation phase 1 study.
Blinding	Not applicable, as this was an open-label study.

Reporting for specific materials, systems and methods

We require information from authors about some types of materials, experimental systems and methods used in many studies. Here, indicate whether each material, system or method listed is relevant to your study. If you are not sure if a list item applies to your research, read the appropriate section before selecting a response.

Materials & experimental systems

n/a	Involved in the study
☐	☒ Antibodies
☒	☐ Eukaryotic cell lines
☒	☐ Palaeontology and archaeology
☒	☐ Animals and other organisms
☐	☒ Clinical data
☒	☐ Dual use research of concern
☒	☐ Plants

Methods

n/a	Involved in the study
☒	☐ ChIP-seq
☐	☒ Flow cytometry
☒	☐ MRI-based neuroimaging

Antibodies

Antibodies used

For PK analysis:
- anti-MP0317 sulfotag labeled mAb (CePower GmbH)
- humanized anti-DARPin mAb (Evitria)

For multiplex immunofluorescence:
- anti-DARPin rabbit mAb (CePower GmbH)
- anti-FAP rabbit mAb (Clone EPR20021, Abcam, cat# ab207178)
- anti-CD68 XP® rabbit mAb (Clone D4B9C, Cell Signaling, cat# 76437S)
- anti-CD40 rabbit mAb (Clone D8W3N, Cell Signaling, cat# 40868)
- anti-CD163 rabbit mAb (Clone EPR19518, Abcam, cat# ab182422)
- anti-CD3ε XP® rabbit mAb (Clone D7A6E™, Cell Signaling, cat# 85061S)
- anti-CD11c (D3V1E) XP® rabbit mAb (Cell Signaling, cat# 45581S)
- anti-cytokeratin Pan Type I/II mouse Ab cocktail (Thermo Fisher, cat# MA5-13156)
- mouse mAb IgG1 isotype control (Clone G3A1, Cell Signaling, cat# 5415S)
- rabbit mAb IgG XP® isotype control (Clone DA1E, Cell Signaling, cat# 3900S)

For flowcytometry:

Panel 1:
- anti-CD19 mouse mAb BV421 (Clone HIB19, BD, cat# 562440)
- anti-CD4 mouse mAb BV510 (Clone SK3, BD, cat# 562971)
- anti-CD3 mouse mAb FITC (Clone SK7, BD, cat# 345764)
- anti-CD16 mouse mAb PE (Clone B73.1, BD, cat# 332779)
- anti-CD56 mouse mAb PE (Clone NCAM16.2, BD, cat# 345812)
- anti-CD45 mouse mAb PerCP-Cy5.5 (Clone 2D1, BD, cat# 332784)
- anti-CD8 mouse mAb APC (Clone SK1, BD, cat# 345775)
- anti-CD14 mouse mAb APC-H7 (Clone MϕP9, BD, cat# 641394)

Panel 2:
- anti-CD86 mouse mAb BB515 (Clone FUN-1, BD, cat# 564545)
- anti-CD40 mouse mAb PE (Clone HB14, BioLegend, cat# 313006)
- anti-CD54 mouse mAb PE/Dazzle594 (Clone HA58, BioLegend, cat# 353118)
- anti-CD16 mouse mAb cFluor BYG710 (Clone 3G8, Cytek, cat# RC-00005)
- anti-CD11c mouse mAb PC7 (Clone BU15, Beckman Coulter, cat# B96763)
- anti-CD141 mouse mAb APC (Clone 1A4, BD, cat# 564123)
- anti-CD20 mouse mAb SparkNIR685 (Clone 2H7, BioLegend, cat# 302366)
- anti-CD127 mouse mAb APC-A700 (Clone R34.34, Beckman Coulter, cat# A71116)
- anti-CD4 mouse mAb APC-H7 (Clone SK3, BD, cat# 641398)
- anti-CD45 mouse mAb APC/Fire810 (Clone HI30, BioLegend, cat# 304076)
- anti-CD1c mouse mAb BV421 (Clone L161, BioLegend, cat# 331526)
- anti-CD56 mouse mAb BV480 (Clone NCAM16.2, BD, cat# 566224)
- anti-CD14 mouse mAb BV510 (Clone M5E2, BioLegend, cat# 301842)
- anti-CD19 mouse mAb BV605 (Clone HIB19, BioLegend, cat# 302244)
- anti-CD3 mouse mAb BV650 (Clone SK7, BioLegend, cat# 563999)
- anti-CD27 mouse mAb BV711 (Clone O323, BioLegend, cat# 302834)
- anti-CD123 mouse mAb BV786 (Clone 7G3, BD, cat# 564196)
- anti-HLA-DR mouse mAb BUV395 (Clone G46-6, BD, cat# 564040)
- anti-CD8 mouse mAb BUV395 (Clone RPA-T8, BD, cat# 612942)
- anti-CD25 mouse mAb BUV563 (Clone 2A3, BD, cat# 612918)
- anti-CD69 mouse mAb BUV737 (Clone FN50, BD, cat# 612817)

For electrochemiluminescence:
- anti-FAP biotinylated polyclonal sheep Ab (R&D systems, cat# DY3715)
- anti-FAP sulfotag labeled rat mAb (Clone D8, Vitatex, cat# MABS1001)
- anti-CD40 biotinylated mouse mAb (MesoScale Discovery, cat# C217B-3)
- anti-CD40 sulfotag labeled mouse mAb (MesoScale Discovery, cat# D217B-3)

Validation

Validation data for commercially available antibodies are provided by the respective manufacturers and can be accessed through their official websites. MP0317 concentrations in serum were assessed by an electrochemiluminescence immunoassay developed and validated at Molecular Partners AG which uses as capture reagent biotinylated human recombinant CD40 (Acrobiosystems AG)

and as detection reagent a sulfotag monoclonal antibody anti-MP0317 (CePower GmbH).
Detection of anti-drug antibodies (ADAs) against MP0317 was performed with a validated method developed at Molecular Partners (electrochemiluminescence-based assay), using as a positive control the humanized anti-DARPin monoclonal antibody (Evitria).

Clinical data

Policy information about clinical studies
All manuscripts should comply with the ICMJE guidelines for publication of clinical research and a completed CONSORT checklist must be included with all submissions.

Clinical trial registration: NCT05098405

Study protocol: The redacted protocol of this study is accessible as supplementary information.

Data collection: Clinical and safety data were collected in the EDC system Clincase and the safety database Argus. Patients were recruited between 11-Oct-2021 and 19-Sep-2023.

Outcomes: Outcomes were defined in the protocol and measures in the statistical analysis plan.
Primary outcome measures and assessments:
Type, incidence and severity of AEs and serious AEs. Assessed according to the national cancer Institute Common Terminology Criteria for Adverse Events (NCI CTCAE) v5.0, from first study drug administration and until 28 days after the last study drug administration or end of study (EOS).
Incidence of dose-limiting toxicities (DLTs). DLTS were reviewed as a subset of AEs that occurred within 4 weeks after first study drug administration (DLT evaluation period).
Maximum Tolerated Dose (MTD). Based on occurrence of DLTs within an adaptive study design following a Bayesian Logistic Regression Model (BLRM). From first study drug administration and until 28 days after the last study drug administration or end of study (EOS).
Secondary outcome measures and assessments:
Serum concentration-time profiles. Including parameters like maximum serum concentration (Cmax), time at Cmax (Tmax), minimal serum concentration (Cmin). Time frame: 4.5 months.
Area under the serum curve (AUC). Pharmacokinetic (PK) analysis of MP0317. Time frame: 4.5 months.
Total clearance (CL). Pharmacokinetic (PK) analysis of MP0317. Time frame: 4.5 months.
Volume of distribution at steady state (Vss). Pharmacokinetic analysis of MP0317. Time frame: 4.5 months.
Half-life (t1/2). Pharmacokinetic (PK) analysis of MP0317. Time frame 4.5 months.
Overall response rate (ORR). Proportion of participants with complete response (CR) and partial response (PR) using Response Evaluation Criteria in Solid Tumors (RECIST) v1.1 and immunotherapy Response Evaluation Criteria in Solid Tumors (iRECIST). Time frame: 4.5 months.
Disease control rate (DCR). Best overall response (BOR) of CR, PR or stable disease (SD) lasting 4 or more weeks following first study drug administration. Time frame: 4.5 months.

Plants

Seed stocks: *Report on the source of all seed stocks or other plant material used. If applicable, state the seed stock centre and catalogue number. If plant specimens were collected from the field, describe the collection location, date and sampling procedures.*

Novel plant genotypes: *Describe the methods by which all novel plant genotypes were produced. This includes those generated by transgenic approaches, gene editing, chemical/radiation-based mutagenesis and hybridization. For transgenic lines, describe the transformation method, the number of independent lines analyzed and the generation upon which experiments were performed. For gene-edited lines, describe the editor used, the endogenous sequence targeted for editing, the targeting guide RNA sequence (if applicable) and how the editor was applied.*

Authentication: *Describe any authentication procedures for each seed stock used or novel genotype generated. Describe any experiments used to assess the effect of a mutation and, where applicable, how potential secondary effects (e.g. second site T-DNA insertions, mosiacism, off-target gene editing) were examined.*

Flow Cytometry

Plots

Confirm that:

- [ ] The axis labels state the marker and fluorochrome used (e.g. CD4-FITC).
- [ ] The axis scales are clearly visible. Include numbers along axes only for bottom left plot of group (a 'group' is an analysis of identical markers).
- [ ] All plots are contour plots with outliers or pseudocolor plots.
- [ ] A numerical value for number of cells or percentage (with statistics) is provided.

Methodology

Sample preparation: Whole blood samples in 4 mL sodium heparin tubes collected at sites as per protocol schedule of assessments were sent at

Sample preparation	ambient temperature for sample preparation and flow cytometry analysis according to validated assay protocols for research use.
Instrument	BD FACS Canto™ II (BD Biosciences, San Jose, CA USA); Cytek® Aurora (Cytek® Biosciences, US);
Software	BD FACSDiva™; SpectroFlo™
Cell population abundance	No cell sorting was performed
Gating strategy	Gating strategies are laid out in the methods section:<br>* Lymphocytes were gated on CD45+SSC, excluding debris and monocytes. T cells (CD3+), B cells (CD19+), and NK cells (CD3−CD16+CD56+) were identified within the lymphocyte gate, with CD4/CD8 defining T cell subsets.<br>* The combination of Fixable viability stain and SSC-A was used to exclude dead cells from analysis and CD45 expression to determine the CD45+Leukocyte population.

☐ Tick this box to confirm that a figure exemplifying the gating strategy is provided in the Supplementary Information.
