# Supplementary material for: Tumor-localized CD40 agonism with MP0317, a FAP x CD40 DARPin, reprograms the tumor microenvironment in patients with advanced solid tumors: an open-label, nonrandomized, dose-escalation phase 1 study
Source: Nat Cancer. 2026 May 1;7(5):810–22. doi: 10.1038/s43018-026-01150-1 (PMC13221297; doi:10.1038/s43018-026-01150-1)
Supplement: Supplementary file 2 — Redacted study protocol. [file 43018_2026_1150_MOESM2_ESM.pdf]

CONFIDENTIAL
CLINICAL STUDY PROTOCOL

**PROTOCOL TITLE**	A phase 1, first-in-human, multicenter, open-label, dose-escalation study to characterize the safety and tolerability of MP0317 in patients with relapsed/refractory advanced solid tumors
**PROTOCOL NUMBER**	MP0317-CP101
**INVESTIGATIONAL DRUG**	MP0317
**DEVELOPMENT PHASE**	Phase 1
**SPONSOR**	Molecular Partners AG<br>Wagistrasse 14<br>8952 Schlieren<br>Switzerland
**EUDRACT NUMBER**	2020-005516-22

VERSION HISTORY

Version Number	Release Date
Version 1.0 (not submitted)	12 November 2020
Version 2.0 (not submitted)	30 April 2021
Version 3.0	17 May 2021
Version 4.0	21 April 2022

The study will be conducted in compliance with the protocol, The International Council for Harmonisation of Technical Requirements for Pharmaceuticals for Human Use (ICH), Good Clinical Practice (GCP) and any applicable regulatory requirements.

CONFIDENTIAL
May not be used, divulged, published, or otherwise disclosed without the consent of
Molecular Partners AG, Schlieren, Switzerland

Signature Page – Authors

PROTOCOL TITLE: A phase 1, first-in-human, multicenter, open-label, dose-escalation study to characterize the safety and tolerability of MP0317 in patients with relapsed/refractory advanced solid tumors

PROTOCOL NUMBER: MP0317-CP101

The information contained in this protocol is consistent with the current benefit/risk evaluation of the study treatment.

This protocol has been approved by Molecular Partners AG. The signatures below document this approval.

**Name**<br>**Title, Company**	**Date**	**Signature**
[redacted]		
[redacted]		
[redacted]		

Signature Page – Principal Investigator

PROTOCOL TITLE: A phase 1, first-in-human, multicenter, open-label, dose-escalation study to characterize the safety and tolerability of MP0317 in patients with relapsed/refractory advanced solid tumors

PROTOCOL NUMBER: MP0317-CP101

I confirm that I have read and understood this protocol and agree to conduct the trial as outlined in the protocol and other information supplied to me. I agree to conduct the trial in accordance with the Declaration of Helsinki and its amendments, Good Clinical Practices (GCP) guidelines established by the International Council for Harmonisation of Technical Requirements for Pharmaceuticals for Human Use (ICH) and applicable local legal and regulatory requirements.

Furthermore, I confirm herewith that the Sponsor is allowed to enter and use my professional contact details and function in an electronic database for internal purposes only.

**Principal Investigator Name, Title, Institution**	**Date**	**Signature**

Signature Page – Coordinating Investigator

PROTOCOL TITLE: A phase 1, first-in-human, multicenter, open-label, dose-escalation study to characterize the safety and tolerability of MP0317 in patients with relapsed/refractory advanced solid tumors

PROTOCOL NUMBER: MP0317-CP101

I confirm that I have read and understood this protocol and agree to conduct the trial as outlined in the protocol and other information supplied to me. I agree to conduct the trial in accordance with the Declaration of Helsinki and its amendments, Good Clinical Practices (GCP) guidelines established by the International Council for Harmonisation of Technical Requirements for Pharmaceuticals for Human Use (ICH) and applicable local legal and regulatory requirements.

Furthermore, I confirm herewith that the Sponsor is allowed to enter and use my professional contact details and function in an electronic database for internal purposes only.

Coordinating Investigator Name, Title, Institution	Date	Signature
[redacted]		

CONTACT INFORMATION

Name and contact information for the Sponsor, Sponsor's Signatory, Sponsor Medical Monitor, Sponsor Project Leader, Sponsor Drug Safety Representative, Clinical Research Organization (CRO), CRO Medical Monitor, Central Laboratory and other clinical service providers are provided separately.

CONFIDENTIAL
May not be used, divulged, published, or otherwise disclosed without the consent of
Molecular Partners AG, Schlieren, Switzerland

TABLE OF CONTENTS

1 BACKGROUND 25
1.1 Medical Need for Safer CD40 Activators to Improve Cancer Immunotherapy .25
[redacted]
1.3 Rationale 29
[redacted] 29
1.3.2 Study Rationale and Benefit Risk Assessment 32
1.3.3 Rationale for DLT Evaluation Period 33
1.3.4 Rationale for Tissue and Blood Biomarker Sample Collection 33
2 STUDY OBJECTIVES AND ENDPOINTS 35
2.1 Objectives 35
2.1.1 Primary 35
2.1.2 Secondary 35
2.1.3 Exploratory 35
2.2 Endpoints 35
2.2.1 Primary 35
2.2.2 Secondary 35
2.2.3 Exploratory 36
3 STUDY DESIGN 37
3.1 Description of the Study 37
3.2 Number of Patients and Study Sites 38
3.3 Duration of Study 38
3.4 End of Study 38
3.5 Blinding and Randomization 38
4 STUDY POPULATION 39
4.1 Patient Population 39
4.2 Patient Inclusion Criteria 39
4.3 Patient Exclusion Criteria 40
5 STUDY TREATMENT AND PROCEDURES 43
5.1 Study Drug Description, Preparation and Dispensing 43
5.1.1 Product Description 43
5.1.2 Study Treatment Preparation 43
5.1.3 Study Treatment Handling and Storage 43
5.1.4 Study Treatment Compliance, Accountability and Destruction 44
5.2 Study Treatment Schedule and Administration 44
5.2.1 Study Drug Dose 44
5.2.2 Study Drug Administration 44
5.2.3 Initiation of a New Study Drug Administration 45
5.2.4 Treatment Period 45
5.2.5 Dose-Limiting Toxicities 45
5.2.6 Dose-Limiting Toxicity Evaluability 46
5.2.7 Dose-Escalation Decisions 46
5.2.8 Safety Waiting Period 48
5.2.9 Treatment Modification (Delay, Interruption) 48
5.2.10 Time Windows for Administration of Study Treatment 50
6 CONCOMITANT MEDICATION 52
6.1 Permitted Therapy for Chronic Diseases and Maintenance Therapy 52
6.2 Management of Infusion Related Reactions and Cytokine Release Syndrome ..52

6.3 Prophylactic Premedication ... 52
6.4 Anticancer Treatment ... 53
6.5 Vaccines ... 53
6.6 Herbal Therapies ... 53
7 VISIT SCHEDULES AND ASSESSMENTS ... 54
7.1 Study Flow and Visit Schedule ... 54
7.1.1 Q3W Dosing Schedule ... 54
7.1.2 Q1W Dosing Schedule ... 62
7.2 Screening Period ... 72
7.2.1 Waivers to Study Protocol ... 72
7.2.2 Patient Enrollment and Eligibility Recommendation ... 72
7.2.3 Treatment Assignment ... 72
7.2.4 Screen Failures ... 72
7.3 Treatment Period and Follow-up ... 73
7.3.1 Treatment Period and End of Treatment ... 73
7.3.2 Safety Follow-up and End of Study ... 73
7.3.3 Survival Follow-up ... 73
7.3.4 Patient Discontinuation ... 74
7.3.5 Withdrawal of Consent ... 74
7.4 Study Assessments ... 74
7.4.1 Medical History and Demography ... 74
7.4.2 Cancer Disease and Treatment History ... 75
7.4.3 Physical Examination and ECOG PS ... 75
7.4.4 Vital Signs ... 75
7.4.5 Pulse Oximetry ... 75
7.4.6 Electrocardiogram ... 75
7.4.7 Echocardiography or MUGA Scan ... 76
7.4.8 Thyroid Function Test ... 76
7.4.9 Urinalysis ... 76
7.4.10 Pregnancy Test ... 76
7.4.11 Local Laboratory (Safety) Assessments ... 76
7.4.12 Central Laboratory Assessments ... 77
7.4.13 Pharmacokinetic Assessments ... 78
7.4.14 Immunogenicity Assessments ... 78
7.4.15 Efficacy Assessments ... 78
7.4.16 Biomarker Assessments ... 78
8 SAFETY PLAN ... 81
8.1 Adverse Events ... 81
8.1.1 Definition and Reporting ... 81
8.1.2 Abnormal Laboratory Results ... 83
8.1.3 Adverse Events of Special Interest ... 83
8.2 Serious Adverse Events ... 84
8.2.1 Definition of an SAE ... 84
8.2.2 Reporting of SAEs/AESIs via SAE/AESI Report Form ... 85
8.3 Pregnancy ... 86
8.4 Overdose ... 86
8.5 Dose Escalation Review Committee and Stopping Rules ... 87
9 STATISTICAL ANALYSES ... 88
9.1 Sample Size Determination ... 88
9.2 Data Handling ... 88
9.2.1 Screen Failures ... 88
9.2.2 Missing Data and Treatment Discontinuation ... 88

9.3 Analysis Sets **88**
9.3.1 Safety Analysis Set 88
9.3.2 Dose-Determining Set 89
9.3.3 Pharmacokinetics Analysis Set 89
9.4 Primary Analysis **89**
9.4.1 Dose-Escalation 89
9.5 Patient Demography and Other Screening Characteristics **92**
9.6 Study Treatment and Concomitant Medication **92**
9.7 Safety Analyses **93**
9.7.1 Adverse Events 93
9.7.2 Laboratory Parameters 93
9.7.3 Vital Signs 94
9.7.4 Electrocardiogram 94
9.8 Pharmacokinetic Analyses **94**
9.9 Immunogenicity Analyses **94**
9.10 Efficacy Analyses **94**
9.11 Pharmacodynamic Analyses **95**
9.12 Exploratory Analyses **95**
9.13 Interim Analyses **96**
10 QUALITY 97
10.1 Data Quality Control and Quality Assurance **97**
10.2 Study Monitoring Requirements **97**
10.3 Case Report Form Completion **97**
10.4 Source Documents **97**
10.5 Data Protection **98**
10.6 Disclosure of Data **98**
10.7 Data Management **98**
10.8 Study Documentation, Record Keeping and Retention of Documents **99**
11 ETHICAL CONSIDERATIONS AND ADMINISTRATIVE PROCEDURES 100
11.1 Regulatory and Ethical Compliance **100**
11.1.1 Protocol and Protocol Amendments 100
11.1.2 Informed Consent Procedure 100
11.2 Responsibilities of the Investigator **100**
11.2.1 Protocol Adherence 101
11.2.2 Protocol Modification and Amendments 101
11.2.3 Coordinating Investigator 101
11.3 Study Termination **101**
11.4 Site Discontinuation **101**
11.5 Publication of Study Protocol and Results **102**
11.6 Dissemination of Clinical Study Data **102**
11.7 Audits and Inspections **102**
11.8 Liability, Insurance and Financial Disclosures **102**
12 APPENDICES 103
12.1 Appendix: ECOG Performance Status **103**
12.2 Appendix: Contraceptive Guidance **104**
12.3 Appendix: Response Evaluation with RECIST v1.1 .... **106**
12.4 Appendix: Evaluation and Guidance Using iRECIST **107**
[redacted] **109**
12.6 Appendix: Management of Infusion Related Reactions **110**
12.7 Appendix: Management of Cytokine Release Syndrome **112**
12.8 Appendix: Statistical Model Performance and Data Scenarios **113**

13 REFERENCES 120

List of Figures

Figure 1. Predicted Serum and Tumor Concentrations of MP0317 for the Proposed Clinical Dose-Escalation Scheme 30
Figure 2. Study Design 37
Figure 3. Prior Medians and 95% Credible Intervals 91
Figure 4. Management of Infusion Related Reactions 111
Figure 5. Management of Cytokine Release Syndrome 112

List of Tables

Table 1. Predicted Exposure Margins for Serum MP0317 31
Table 2. MP0317 Dose Calculation and Escalation for a Q1W Schedule based on Exposure-Matching Criteria 32
Table 3. Provisional Dose Levels for Dose-Escalation given Q3W or Q1W 47
Table 4. Guidelines for Study Treatment Modifications 48
Table 5. Time Windows for Administration of Study Treatment (Q3W) 50
Table 6. Time Windows for Administration of Study Treatment (Q1W) 51
Table 7. Schedule of Assessments (Q3W) 54
Table 8. Schedule of Assessments: Cycle 1 Detailed (Q3W) 58
Table 9. Schedule of Assessments: Cycle 2 Detailed (Q3W) 58
Table 10. Schedule of Assessments: Cycle 3 Detailed (Q3W) 59
Table 11. Schedule of Assessments: Cycle 4 Detailed (Q3W) 59
Table 12. Schedule of Assessments: Cycle 5 Detailed (Q3W) 60
Table 13. Schedule of Assessments: Cycle 6 and All Further Cycles Detailed (Q3W) 60
Table 14. Schedule of Assessments (Q1W) 62
Table 15. Schedule of Assessments: Cycle 1 Detailed (Q1W) 65
Table 16. Schedule of Assessments: Cycle 2 Detailed (Q1W) 66
Table 17. Schedule of Assessments: Cycle 3 Detailed (Q1W) 67
Table 18. Schedule of Assessments: Cycle 4 Detailed (Q1W) 68
Table 19. Schedule of Assessments: Cycle 5 Detailed (Q1W) 69
Table 20. Schedule of Assessments: Cycle 6 and All Further Cycles Detailed (Q1W) 70
Table 21. Local Laboratory Panel and Parameters 77
Table 22. SAE Definition 84
Table 23. Summary of Prior Distribution 90
Table 24. Prior Probabilities of DLT at Selected Doses 91
Table 25. RECIST v1.1 Guidelines for Tumor Response 106
Table 26. RECIST v1.1 Overall Response Criteria 106
Table 27. iRECIST Response Criteria Definitions 107
Table 28. iRECIST Guidelines for Progressive Disease Evaluation 108
[redacted] 109
Table 30. Hypothetical Data Scenarios 113
Table 31. Assumed True Dose-Toxicity Scenarios 118
Table 32. Simulated Operating Characteristics 118

List of Abbreviations and Acronyms

Abbreviation/Acronym	Full Term
ADA	Anti-drug antibodies
AE	Adverse event
AESI	Adverse event of special interest
ALP	Alkaline phosphatase
ALT	Alanine aminotransferase
APC	Antigen presenting cell
aPTT	Activated partial thromboplastin time
AST	Aspartate aminotransferase
ATC	Anatomical Therapeutic Classification
AUC	Area under the curve
$AUC_{(0\text{-}28days)}$	Area under the curve from first administration to 28 days
$AUC_{(0\text{-}inf)}$	Area under the curve from administration to infinity
BLRM	Bayesian Logistic Regression Model
BOR	Best overall response
BUN	Blood urea nitrogen
CHO	Chinese hamster ovary
CI	Confidence interval
CK	Creatine kinase
CL	Total clearance
C_{max}	Maximum serum concentration
C_{min}	Minimum serum concentration
CNS	Central nervous system
COPD	Chronic obstructive pulmonary disease
CR	Complete response
CRA	Clinical research associate
CrI	Credible interval
CRO	Clinical research organization
CRP	C-reactive protein
CRS	Cytokine release syndrome
CSR	Clinical Study Report
CT	Computed tomography
ctDNA	Circulating tumor DNA
CV	Confidence value
DC	Dendritic cell
DCR	Disease control rate
DDS	Dose-determining set
DERC	Dose-Escalation Review Committee
DNA	Deoxyribonucleic acid
DLT	Dose-limiting toxicity
DOR	Duration of response
EC_{20}	20% effective concentration
ECG	Electrocardiogram
ECOG	Eastern Cooperative Oncology Group

Abbreviation/Acronym	Full Term
eCRF	Electronic case report form
ELISA	Enzyme linked immune-sorbent assay
EMA	European Medicines Agency
EOI	End of infusion
EOS	End of study
EOT	End of treatment
EU	European Union
EudraCT	European Union Drug Regulating Authorities Clinical Trials Database
EWOC	Escalation With Overdose Control
FACS	Fluorescence activated cell sorting
FAP	Fibroblast activation protein
FCBP	Female of childbearing potential
FDA	Food and Drug Administration
FFPE	Formalin-fixed paraffin-embedded
FIH	First-in-human
FSH	Follicle stimulating hormone
FU	Follow-up
GCP	Good Clinical Practice
GFR	Glomerular filtration rate
GLP	Good Laboratory Practice
GnRH	Gonadotropin-releasing hormone
h	Hour(s)
HBsAg	Hepatitis B surface antigen
HBV	Hepatitis B virus
HCV	Hepatitis C virus
HIV	Human immunodeficiency virus
HNSCC	Head and neck squamous cell carcinoma
HNSTD	Highest non-severely toxic dose
HRT	Hormonal replacement therapy
HSA	Human serum albumin
IB	Investigator's Brochure
ICF	Informed consent form
ICH	International Council for Harmonisation of Technical Requirements for Pharmaceuticals for Human Use
ICMJE	International Committee of Medical Journal Editors
iCR	Immune complete response
iCPD	Immune confirmed progressive disease
Ig	Immunoglobulin
iUPD	Immune unconfirmed progressive disease
IEC	Independent Ethics Committee
IF	Immunofluorescence
IFN	Interferon
IHC	Immunohistochemistry
IL	Interleukin
IMP	Investigational medicinal product
INR	International normalized ratio

Abbreviation/Acronym	Full Term
iPD	Immune progressive disease
iPR	Immune partial response
IRB	Institutional Review Board
iRECIST	Immunotherapy Response Evaluation Criteria in Solid Tumors
IRR	Infusion related reaction
iSD	Immune stable disease
iSOD	Immune sum of diameters
IV	Intravenous
IVSS	IV solution stabilizer
K_D	Binding constant
LDH	Lactate dehydrogenase
LVEF	Left ventricular ejection fraction
Ly6H	Lymphocyte antigen 6H
MABEL	Minimum anticipated biological effect level
mAb	Monoclonal antibody
MCH	Mean corpuscular hemoglobin
MCHC	Mean corpuscular hemoglobin concentration
MCV	Mean corpuscular volume
MedDRA	Medical Dictionary for Regulatory Activities
MOA	Mode of action
MRI	Magnetic resonance imaging
mRNA	Messenger RNA
MRSD	Maximum recommended starting dose
MSD	Meso scale discovery
MTD	Maximum tolerated dose
MUGA	Multi-gated acquisition
NCI CTCAE	National Cancer Institute Common Terminology Criteria for Adverse Events
NGS	Next generation sequencing
NK (cell)	Natural killer (cell)
NL	New lesion
NOAEL	No observed adverse effect level
NSCLC	Non-small cell lung cancer
NTL	Non-target lesion
ORR	Overall response rate
OS	Overall survival
PBMC	Peripheral blood mononuclear cell
PCR	Polymerase chain reaction
PD	Progressive disease
PFS	Progression-free survival
PI	Product Information
PK	Pharmacokinetic(s)
PO	Per os
PR	Partial response
PS	Performance status
PT	Prothrombin time

Abbreviation/Acronym	Full Term
q1w	Every week
q3w	Every 3 weeks
QT	Time from the start of the Q wave to the end of the T wave
QTc	QT corrected
RBC	Red blood cell
RDE	Recommended dose for expansion
RECIST	Response Evaluation Criteria in Solid Tumors
RNA	Ribonucleic acid
SAE	Serious adverse event
SAS	Safety analysis set
SAP	Statistical Analysis Plan
sCD40	Soluble CD40
SD	Stable disease
sFAP	Soluble FAP
SJS	Stevens-Johnson Syndrome
SmPC	Summary of Product Characteristics
SOC	System Organ Class
SPECT	Single-photon emission computed tomography
STD	Standard deviation
SUSAR	Suspected unexpected serious adverse reaction
$t_{1/2}$	Half-life
TEAE	Treatment emergent adverse event
TEN	Toxic epidermal necrolysis
TL	Target lesion
t_{last}	Time to last measurable serum concentration
t_{max}	Time to maximum serum concentration
TNF	Tumor necrosis factor
TSH	Thyroid-stimulating hormone
TTP	Time to progression
ULN	Upper limit of normal
V_{ss}	Volume of distribution at steady state
WBC	White blood cell
WHO	World Health Organization

CONFIDENTIAL
May not be used, divulged, published, or otherwise disclosed without the consent of
Molecular Partners AG, Schlieren, Switzerland

Definition of Terms

Term	Definition
Enrolled	Informed consent form fully signed.
Screening	The screening period starts at the informed consent date, lasts for a maximum of 28 days and ends on the day of the first study drug administration.
Patient End of Treatment	The date when the decision is taken for the patient to discontinue from study treatment for any reason.
Safety Follow-up	A safety follow-up visit will be scheduled at Week 4 after the last study drug administration.
Patient End of Study	Completion of safety follow-up or discontinuation from the study for any reason.
Survival Follow-up	After patient end of study, survival follow-up will be performed to collect survival status approximately 3 and 6 months after patient end of study or until withdrawal of consent, patient is lost to follow-up, death or study termination by the Sponsor, whichever occurs first.
End of Study	Date when the last patient/last visit occurs or when the last data point required for statistical analysis or safety follow-up is received from the last patient, whichever occurs later.
Screen Failure	Patient who consented to participate in the clinical study, but who did not receive study treatment for any reason.
Lost to Follow-up	Patients who miss 3 consecutive study contacts by clinic visits and/or telephone
Dose-Limiting Toxicity Evaluation Period	Four-week period after first study drug administration.
End of Infusion	Defined as completing the investigational medicinal product infusion including flushing the entire infusion line with 25 mL of 0.9% NaCl (at the same rate as the infusion itself).

PROTOCOL SUMMARY

**Study Title**	A phase 1, first-in-human, multicenter, open-label, dose-escalation study to characterize the safety and tolerability of MP0317 in patients with relapsed/refractory advanced solid tumors
**Study Number**	MP0317-CP101
**Protocol Version**	4.0
**Sponsor**	Molecular Partners AG<br>Wagistrasse 14<br>8952 Schlieren<br>Switzerland
**EudraCT Number**	2020-005516-22
**Number of Sites and Countries**	The study will be conducted in a total of 4 sites in France and the Netherlands, in 2 sites in each country. Additional sites and countries may be considered during the study.
**Investigational Medicinal Product**	MP0317<br>A tri-specific fibroblast activation protein (FAP) x CD40 DARPin® drug candidate
**Phase**	1
**Indication**	Relapsed/refractory advanced solid tumors
**Study Rationale**	MP0317 (FAPxCD40) is a tri-specific FAP-targeting DARPin® molecule designed to combine high potency for CD40 activation with tumor-targeting and tumor-restricted receptor engagement and immune cell activation. By localizing and restricting the agonistic effect of CD40 to the tumor site, MP0317 is expected to limit the risk of potential systemic side effects (such as cytokine release syndrome [CRS]) and to increase the therapeutic window of CD40 activation.<br>The study population consists of cancer patients with advanced solid tumor types, which have been known to express medium to high levels of FAP[1, 2, 3] and for whom approved therapies have been exhausted or who are ineligible or unable to tolerate other treatments.<br>Future clinical development will focus on MP0317 in combination with other treatment modalities (e.g. checkpoint inhibitors, chemotherapy, radiotherapy).
**Study Design**	This is a phase 1, first-in-human (FIH), multicenter, open-label, dose-escalation study followed by a safety expansion part, evaluating the safety, tolerability, pharmacokinetics (PK), pharmacodynamics and preliminary antitumor activity of MP0317 in adult patients with advanced solid tumors.<br>The dose-escalation part is designed to determine the recommended dose for expansion (RDE) or the maximum tolerated dose (MTD) for MP0317 monotherapy. The safety expansion part is designed to confirm safety in a larger population (see figure below). The dose-escalation scheme will use an adaptive study design following a Bayesian Logistic Regression Model (BLRM). A dose-escalation review committee (DERC) will monitor safety and govern all cohort dosing decisions.<br>The sponsor in consultation with the DERC may advise on the opening of cohorts with alternative dosing schedules (e.g. every week; q1w). Such additional cohorts may be opened concurrently with the initial dosing schedule cohorts, during dose escalation as well as during safety expansion phase.

<table>
<tr><td></td><td colspan="2">
Once the RDE (or MTD) has been determined, the safety expansion cohort(s) will be opened and up to 15 additional patients will be treated with MP0317 monotherapy at this dose.
The first doses between the first 2 patients in any cohort must be separated by a minimum of 7 days.
Study treatment will be administered until progressive disease (PD), unacceptable toxicity, withdrawal of consent or other reasons to discontinue treatment occur, whichever comes first. Treatment beyond PD will be allowed as per Immunotherapy Response Evaluation Criteria in Solid Tumors (iRECIST)[4].
Paired (pre and on/post-treatment) tumor and skin biopsies are mandatory for all patients.
Biomarkers that may potentially correlate with antitumor activity or immunomodulatory effects of MP0317 may be explored during the study.
Study Design
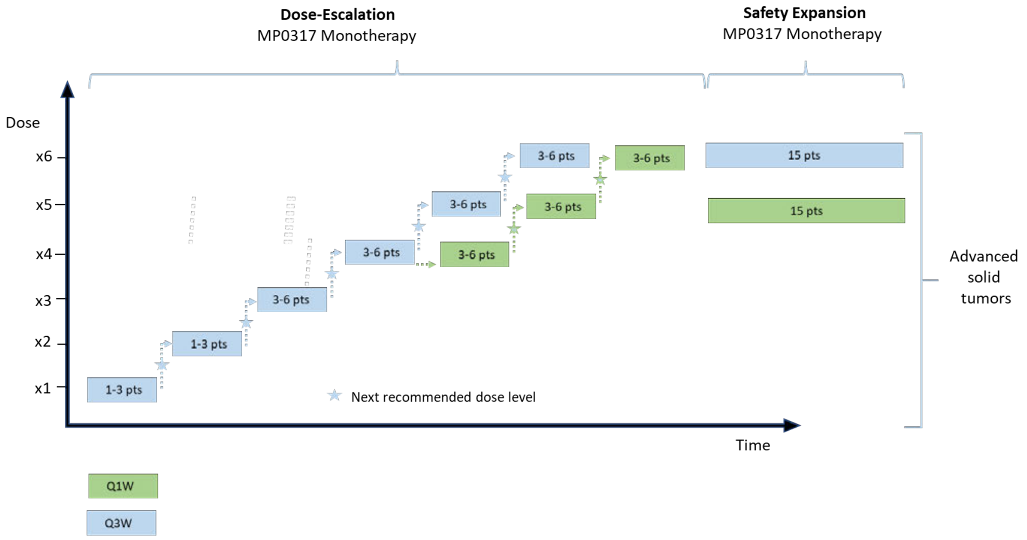

Pts, patients; Q1W, every week dosing schedule; Q3W, every 3 weeks dosing schedule.
</td></tr>
<tr><td rowspan="2">Primary Objectives and Endpoints</td><td>Objectives
• To determine the RDE or the MTD for MP0317 as monotherapy in patients with advanced solid tumors (dose-escalation part only)</td><td>Endpoints
• Incidence of dose-limiting toxicities (DLTs)</td></tr>
<tr><td>• To characterize the safety and tolerability of MP0317 as monotherapy in patients with advanced solid tumors</td><td>• Type, incidence and severity of adverse events (AEs) and serious adverse events (SAEs) according to the National Cancer Institute Common Terminology Criteria for Adverse Events (NCI CTCAE) v5.0
• Changes between screening and post-screening laboratory parameters and vital signs</td></tr>
<tr><td>Secondary Objectives and Endpoints</td><td>Objectives
• To describe the PK of MP0317 as monotherapy in patients with advanced solid tumors</td><td>Endpoints
• Serum concentration-time profiles following first and repeated MP0317 infusions</td></tr>
</table>

		• Determination of PK parameters including (but not limited to) maximum serum concentration (C_{max}), time to C_{max} (T_{max}), minimal serum concentration (C_{min}), area under the curve (AUC), total clearance (CL), volume of distribution at steady state (V_{ss}) and half-life ($t_{1/2}$)
	• To evaluate preliminary antitumor activity of MP0317 as monotherapy in patients with advanced solid tumors	• Overall response rate (ORR) based on best overall response (BOR) of complete response (CR) and partial response (PR) locally assessed using Response Evaluation Criteria in Solid Tumors (RECIST) v1.1 and iRECIST<br>• Disease control rate (DCR) of CR, PR or stable disease (SD) lasting 4 or more weeks following the initiation of MP0317<br>• Duration of response (DOR) of CR or PR based on RECIST v1.1 and iRECIST, time to progression (TTP) following the initiation of MP0317
	• To evaluate preliminary clinical benefit of MP0317 as monotherapy in patients with advanced solid tumors	• Progression-free survival (PFS) based on RECIST v1.1 and iRECIST<br>• Overall survival (OS)
**Exploratory Objectives and Endpoints**	**Objectives**<br>• To evaluate pharmacodynamic effects of MP0317 as monotherapy in peripheral blood and tissue in patients with advanced solid tumors	**Endpoints**<br>• Changes in frequency and functionality of B cells, dendritic cells (DC), macrophages and T cell subsets in peripheral blood and tissue biopsies<br>• Assess FAP and CD40 expression and co-localization with MP0317 in tissue biopsies<br>• Changes in cytokines in serum<br>• Changes in soluble FAP (sFAP) and soluble CD40 (sCD40) in serum<br>• Circulating tumor DNA (ctDNA) (safety expansion part only)
	To evaluate the immunogenicity of MP0317 as monotherapy in patients with advanced solid tumors	• Occurrence of anti-drug antibodies (ADAs)<br>• Incidence, titer and time-course of ADAs
**Inclusion Criteria**	A patient is eligible to be included in the study if they meet all of the following criteria:<br>1. Has an advanced, histologically-proven solid tumor of one of the following types, and for which approved therapies have been exhausted or for which the Investigator considers the patient ineligible or unable to tolerate other treatments:<br>a. Colorectal cancer<br>b. Ovarian cancer	

	c. Endometrial cancer<br>d. Gastric cancer<br>e. Pancreatic cancer<br>f. Anal cancer<br>g. Cervical cancer<br>h. Head and neck squamous cell carcinoma (HNSCC)<br>i. Mesothelioma<br>j. Prostate cancer<br>k. Non-small cell lung cancer (NSCLC)<br>l. Melanoma<br>m. Urothelial/bladder cancer<br>n. Microsatellite instability high cancer of any type<br>o. Cutaneous squamous cell cancer<br>p. Breast cancer<br>2. ≥ 18 years of age on the day of signing informed consent<br>3. Has signed and dated written informed consent before performing any study procedure, including screening<br>4. Eastern Cooperative Oncology Group (ECOG) performance status (PS) 0 to 1<br>5. Anticipated life expectancy ≥ 12 weeks by Investigator judgement<br>6. Measurable disease according to Response Evaluation Criteria in Solid Tumors (RECIST) v1.1<br>7. Should agree to undergo mandatory paired (pre and on-treatment) tumor biopsies and be considered to have biopsiable disease. The biopsies should be performed as follows:<br>a. At least 1 tumor lesion ≥ 20 mm amenable to percutaneous biopsy other than the target lesion(s) used to follow response as defined by RECIST v1.1.<br>b. For cutaneous or subcutaneous lesions, tumors should be ≥ 5 mm in diameter amenable to biopsy by excisional or punch biopsies without unacceptable risk of a major procedural complication.<br>c. For core needle biopsy specimens, at least 3 to 6 cores with an 18-gauge needle should be collected.<br>d. The on-treatment tumor biopsy should be taken from the same lesion as the pre-treatment biopsy. The biopsied lesion should be large enough to take both biopsies ≥ 1 cm apart.<br>8. Should agree to undergo mandatory paired (pre and on-treatment) skin biopsies<br>9. At least 28 days must have elapsed between any prior major surgery and screening. The following procedures are not considered major:<br>a. Obtaining the pre-treatment tumor and skin biopsies as per protocol requirements<br>b. Placement of a port for central venous access<br>c. Needle, punch or excisional biopsy of a clinically or radiographically detected lesion<br>10. Laboratory parameters at screening:<br>a. Hematology:<br>i. Platelet count ≥ 100,000 cells/mm^3<br>ii. Absolute neutrophil count ≥ 1,000 cells/mm^3

	iii. Hemoglobin ≥ 9 g/dL<br>b. Serum creatinine < 1.5 x upper limit of normal (ULN) or creatinine clearance > 50 mL/min on the basis of Cockcroft-Gault glomerular filtration rate estimation<br>c. Coagulation:<br>i. International normalized ratio (INR) < 1.5<br>ii. Prothrombin time (PT) and activated partial thromboplastin time (aPTT) ≤ 1.5 x ULN unless therapeutically warranted<br>d. Aspartate aminotransferase (AST) and alanine aminotransferase (ALT) < 3 x ULN<br>e. Bilirubin normal, except for patients with known familial hyperbilirubinemia (such as Gilbert syndrome); for patients with documented Gilbert's syndrome (Gilbert-Meulengracht syndrome) total bilirubin ≤ 3 x ULN is acceptable<br>f. Albumin > 2.8 g/dL or > 28 g/L, and without albumin transfusion for ≥ 7 days before screening<br>11. Is using highly effective contraception, for females of childbearing potential (FCBP) and for men, as follows and as defined in Appendix 12.2:<br>a. <u>Female:</u> Is not pregnant, is not breastfeeding, and one of the following applies:<br>- Not a FCBP<br>- A FCBP who agrees and/or whose male partner agrees to follow the contraceptive guidance from screening, during the treatment period, and for at least 3 months after the last study drug administration. A FCBP must have a negative serum pregnancy test result at screening.<br>b. <u>Male:</u> Agreement to use a highly effective contraception method from screening, during the treatment period, and for at least 3 months after the last study drug administration and to refrain from donating sperm during this period.
**Exclusion Criteria**	A patient will be ineligible if one or more of the following statements are applicable:<br>1. Known hypersensitivity to excipients used in the MP0317 formulation<br>2. Autoimmune diseases, except autoimmune endocrinopathies that are stable with hormone replacement therapy<br>3. Inflammatory diseases such as arthritis, colitis, liver fibrosis, cirrhosis, interstitial fibrosis or chronic obstructive pulmonary disease (COPD) that may have elevated tissue fibroblast activation protein (FAP) expression unless approved after consultation with the Sponsor<br>4. Serious illness or concomitant non-oncological disease considered by the Investigator to be incompatible with participating in the protocol<br>5. Left ventricular ejection fraction of < 50% on echocardiographic exam or multi-gated acquisition (MUGA) scan at screening<br>6. History or evidence of clinically significant cardiovascular disease defined as at least one of the following criteria:<br>a. Evidence of poorly controlled arterial hypertension (systolic blood pressure > 160 mmHg or diastolic blood pressure > 100 mmHg)<br>b. Myocardial infarction or instable angina pectoris within 6 months before screening<br>c. Heart failure (New York Heart Association Class III or IV)<br>d. Any cardiac arrhythmia that is not well controlled

	e. QT corrected (QTc) prolongation ≥ Grade 2 (> 480 ms) at screening measured on 2 separate electrocardiograms (ECG) at least 10 minutes apart<br>f. Clinically significant valvular heart disease<br>7. Severe dyspnea, pulmonary dysfunction or need for continuous supportive oxygen inhalation<br>8. Arterial thromboembolic event, stroke or transient ischemia attack within 12 months before screening<br>9. Known central nervous system (CNS) metastases that are either untreated or are treated but are associated with clinical symptoms (e.g. headache, convulsions); patients with CNS metastases that have been treated with radiotherapy and/or surgery are eligible if they are clinically without symptoms for at least 6 weeks before screening; if under treatment with corticosteroids (not exceeding 10 mg/day prednisone or equivalent) and/or anticonvulsive agents, patients must be on a stable dose for at least 14 days before first study drug administration.<br>10. Active uncontrolled bleeding or a bleeding diathesis<br>11. Therapy for active infection needs to be completed at least 7 days before first study drug administration<br>12. Known positivity for human immunodeficiency virus (HIV) or history of HIV (HIV testing is not mandatory)<br>13. Active hepatitis B (chronic or acute; HBV) defined as having a positive hepatitis B surface antigen (HBsAg) test at screening. Patients with past or resolved HBV infection (defined as having a negative HBsAg test and a positive hepatitis B core antigen antibody test) are eligible.<br>14. Active hepatitis C (HCV) infection defined as having a positive HCV antibody test followed by a positive HCV ribonucleic acid (RNA) test at screening. The HCV RNA test will be performed only for patients who have a positive HCV antibody test. Patients who are positive for HCV antibodies are eligible only if polymerase chain reaction (PCR) is negative for HCV RNA.<br>15. Serious or non-healing wound, skin ulcer or non-healing bone fracture<br>16. Abdominal fistula, gastrointestinal perforation or intra-abdominal abscess within 6 months before screening<br>17. Any vaccines within 28 days before first study drug administration (clarification in Section 6.5)<br>18. An allogenic tissue/solid organ transplant<br>19. History of another primary malignancy except for:<br>a. Malignancy treated with curative intent and with no known active disease ≥ 2 years before screening and of relatively low potential risk for recurrence<br>b. Adequately treated non-melanoma skin cancer or lentigo maligna without evidence of residual disease<br>c. Adequately treated carcinoma in situ without evidence of disease<br>d. Cancer patients with incidental histologic findings of prostate cancer that, in the opinion of the Investigator, is not deemed to require active therapy (e.g. incidental prostate cancer identified following cystoprostatectomy that is tumor/node/metastasis Stage ≤ pT2N0) may be eligible, pending discussion and approval by the Sponsor<br>20. Previous treatment with a DARPin® molecule<br>21. Concurrent enrollment in another clinical study, unless it is an observational (non-interventional) clinical study, or it is the follow-up period of an interventional study<br>22. Use of an investigational agent within 28 days before first study drug administration

	23. Any anticancer treatment, including chemotherapy, hormonal therapy or radiotherapy, within 21 days before first study drug administration; however, the following are allowed:<br>a. Hormonal therapy with gonadotropin-releasing hormone (GnRH) agonists or antagonists<br>b. Hormone-replacement therapy or oral contraceptives<br>c. Palliative radiotherapy for bone metastases within 14 days before first study drug administration<br>24. Continuous corticosteroid use exceeding 10 mg/day prednisone or equivalent<br>25. Any condition that, in the opinion of the Investigator, would interfere with evaluation of the investigational medicinal product (IMP) or interpretation of the patient's data<br>26. Unable or unwilling to comply with all study requirements for clinical visits, examinations, tests and procedures<br>27. Patient deprived of liberty by a judicial or administrative decision, patient admitted to a social institution or who is under a measure of legal protection, patient hospitalized without consent or who is in an emergency situation
**Study Treatment/ Administration**	**Dose formulation:** MP0317 is supplied as a concentrate solution for intravenous (IV) administration.<br>**Route of administration and treatment schedule:** The starting dose is 0.03 mg/kg every 3 weeks (q3w) and up to 6 dose levels are planned. A treatment cycle will be 3 weeks (21 days). The sponsor in consultation with the DERC may advise on the exploration of cohorts with alternative dosing schedules (e.g. q1w). Such additional cohorts may be opened concurrently with the initial q3w dosing schedule cohorts, during dose escalation as well as during safety expansion phase. The infusion duration should be at least 50 min (excluding flushing) and no longer than 2 h (including flushing). Usage of a 0.2 µm in-line filter is mandatory.
**Concomitant Medication**	Concomitant medication includes any medication taken within 28 days before the first study drug administration and until 28 days after the last study drug administration or patient end of study (EOS).<br>Stable doses of medications administered for chronic diseases are allowed.<br>Systemic corticosteroids and tumor necrosis factor (TNF)-α inhibitors may be administered at the discretion of the Investigator after consultation with the Sponsor.<br>Hormonal therapy with GnRH agonists or antagonists for prostate cancer, oral contraceptives, hormone-replacement therapy, prophylactic or therapeutic anticoagulation therapy should be continued.<br>Patients who experience infusion related reactions (IRRs) should be treated symptomatically with paracetamol (acetaminophen), ibuprofen (or another antipyretic drug), diphenhydramine (or another antihistamine drug) and/or cimetidine (or another H2 receptor antagonist) and, if considered necessary, corticosteroids, according to institutional standard practice. Serious symptoms of IRRs should be managed with supportive care.<br>Prophylactic premedication to prevent IRRs may be administered at the discretion of the Investigator. During the second DERC meeting held on 9th March 2022, based on safety data from the first two q3w dosed cohorts, the DERC members strongly recommended prophylactic premedication of patients with antihistamine and paracetamol. Furthermore, based on safety information emerging during the course of the study the DERC may consider mandating prophylactic premedication to manage IRRs.<br>Live vaccines are prohibited within 28 days before first study drug administration, during treatment and for 5 months following the last study drug administration.

CONFIDENTIAL
May not be used, divulged, published, or otherwise disclosed without the consent of
Molecular Partners AG, Schlieren, Switzerland

	Other vaccines (such as inactivated [e.g. seasonal flu], subunit, toxoid, or messenger RNA [mRNA] based [e.g. COVID-19] etc.) are prohibited within 28 days before first study drug administration and during the 4-week period after first study drug administration (i.e. patients can be vaccinated with these compounds starting from Cycle 2 Day 8). However, depending on a risk benefit assessment, individual patients could also be vaccinated within the prohibited time period after consultation with the Sponsor.
**Visit Schedule**	Patients will attend visits for screening, study treatment and monitoring, including regular tumor assessment, tissue biopsy collection, PK and biomarker assessments according to the schedule of assessments.<br>The screening period starts at the informed consent date, up to 28 days before the first study drug administration. Study treatment will be administered until the decision is taken for the patient to discontinue from treatment for any reason (i.e. end of treatment [EOT]). A safety follow-up (FU) visit will be scheduled at Week 4 after the last study drug administration. Completion of safety FU or discontinuation from the study for any reason defines patient EOS. Thereafter, survival FU will be performed to collect survival status approximately 3 and 6 months after patient EOS or until withdrawal of consent, patient is lost to follow-up, death or study termination by the Sponsor, whichever occurs first.
**Measurements and Assessments**	**Safety:** Physical examination, ECOG PS, vital signs (including body weight), pulse oximetry, ECG, echocardiography/MUGA, laboratory parameters (including hematology, clinical chemistry, coagulation, liver and kidney function, cardiac and inflammatory parameters, immunology, thyroid function), and urinalysis will be assessed at the protocol-defined time points. DLTs, AEs including SAEs and AEs of special interest (AESIs), graded according to NCI CTCAE v5.0, will be monitored continuously.<br>**Pharmacokinetics:** Blood samples will be collected from all patients who are scheduled for the first study drug administration, to measure serum concentrations of MP0317 before treatment start and on study at protocol-defined time points. AUC, CL and other PK parameters will be determined.<br>**Immunogenicity:** Blood samples will be collected from all patients who are scheduled for the first study drug administration to assess serum titers of ADA before treatment start and on study at protocol-defined time points.<br>**Efficacy:** Antitumor activity for evaluable patients will be evaluated radiographically at protocol-defined time points and patients' clinical response status will be classified according to RECIST v1.1[5] and iRECIST[4] criteria.<br>**Pharmacodynamics and exploratory biomarkers:** Whole blood, serum/plasma and tissue samples (tumor archival and fresh, and skin) will be collected for biomarker evaluations before treatment start and on study at protocol-specified time points.
**Number of Patients**	Up to 6 provisional dose levels are planned for this study with at least 1 to 3 patients per dose level. It is estimated that between 17 and 30 patients will be included for the dose-escalation part of the q3w schedule, and 12 to 18 patients for the dose-escalation part of the q1w schedule. Up to 15 patients treated at the RDE (or MTD) will be included in the safety expansion part of each dosing schedule, giving a total of between 32 and 78 patients. The actual number of patients will depend on the number of dose levels/cohorts that are tested, the number of patients considered non-evaluable for the dose-determining set (DDS) and who are replaced, and the safety profile seen at each dose level.
**Statistical Considerations**	**Study analysis sets**<br>*Safety analysis Set (SAS):* All patients who received at least one dose of MP0317 and had at least one post-dose safety assessment. The SAS will be the primary population for all demography, safety, immunogenicity, efficacy and pharmacodynamic related endpoints, except for determination of the dose-DLT relationship.<br>*Dose-determining Set (DDS):* All patients in the SAS, who either a) receive at least 2 administrations of MP0317 at q3w dosing schedule or 3 administrations at q1w dosing

	schedule at the assigned dose level and are observed until pre-dose of Cycle 2 Day 8 or b) experience a DLT as defined in the protocol during the DLT evaluation period. The DLT evaluation period is defined as the 4-week period after first study drug administration. Patients who do not meet these minimum treatment and safety evaluation requirements will be regarded as ineligible for inclusion in the DDS and will be replaced if needed until the minimum number of patients required for evaluation is reached. The DDS will be used in the BLRM to estimate the dose-DLT relationship in the dose-escalation part of the study.<br>*PK Set:* All patients who received at least one dose of MP0317 and had at least one post-dose PK measurement.<br>**Statistical methodology**<br>The detailed methodology for summary and statistical analyses of the data collected in this study will be documented in a Statistical Analysis Plan (SAP).<br>Data will be analyzed and presented by dose level and study part.<br>*Primary analysis*<br>An adaptive BLRM guided by the Escalation With Overdose Control (EWOC) principle will be used in the dose-escalation part to determine the RDE (or MTD).<br>*Safety analyses*<br>Safety will be assessed through summaries of DLTs, AEs, SAEs, AESIs, AEs leading to treatment adaptation or study drug discontinuation, changes in laboratory parameters, changes in vital signs and ECGs and exposure to MP0317. For the dose-escalation part, the summary of DLTs will be performed using the DDS. Unless specified otherwise, all other safety analyses will be conducted for the SAS.<br>*PK analyses*<br>No formal statistical analysis beyond descriptive statistics is planned. For each PK parameter, individual and mean data and summary statistics will be presented.<br>*Efficacy analyses*<br>All efficacy analyses will be done according to investigator-assessed response criteria. The following antitumor activity endpoints will be analyzed and summarized for the SAS: ORR, BOR, DCR, DOR, TTP, PFS and OS.<br>*Immunogenicity analyses*<br>Incidence of ADA response and the potential correlation with PK, pharmacodynamic and safety parameters may be assessed.<br>*Pharmacodynamic analyses*<br>Summary statistics of pharmacodynamic markers will be reported.<br>**Interim analyses**<br>Each dose-escalation step is considered to be an interim analysis. The BLRM will be updated with the respective number of patients treated and the number of DLTs observed in the last cohort. The updated model will then give a statistical recommendation for the next escalation step. In addition, a risk-benefit assessment that includes a comprehensive analysis of safety and available clinical information will be done to decide on the next escalation steps.
**Duration of Study**	The overall study duration is from the time of recruitment of the first patient until the overall EOS. The duration of the study will depend on safety and efficacy events encountered during the dose-escalation and the safety expansion parts and the rate of patient accrual.

CONFIDENTIAL
May not be used, divulged, published, or otherwise disclosed without the consent of
Molecular Partners AG, Schlieren, Switzerland

	Estimated time from when the study opens to enrollment until completion of data analyses is Q3 2021 to Q3 2023.

CONFIDENTIAL
May not be used, divulged, published, or otherwise disclosed without the consent of
Molecular Partners AG, Schlieren, Switzerland

1 BACKGROUND

1.1 Medical Need for Safer CD40 Activators to Improve Cancer Immunotherapy

The introduction of immune checkpoint inhibitors to cancer therapy has demonstrated the potential of enhancing antitumor immunity to improve patient outcome but has also shown that checkpoint inhibition alone is insufficient and thus ineffective in many cases. Consequently, many other agents involved in controlling immune responses are being investigated for their ability to improve tumor immunotherapy but marked improvements in treatment outcome have not yet been made. Amongst approaches under examination, one of the most frustrating has been CD40 agonism. This has been under investigation for many years but to-date its preclinical promise has not translated into clinical efficacy due to limitations imposed by dose-limiting peripheral immunotoxicity.

CD40 expressed on antigen presenting cells (APCs), particularly dendritic cells (DCs) and macrophages, plays a key role in regulating immune responses and triggering the induction of adaptive immunity. Binding of its trimeric ligand CD40L, which is mainly expressed by T helper cells, leads to CD40 clustering and activation of multiple downstream signaling pathways[6, 7]. As a result, APCs become activated resulting in increased expression of co-stimulatory molecules as well as production of proinflammatory cytokines and enhanced antigen processing and presentation[8, 9], creating an inflammatory milieu capable of activating humoral and cell-mediated antitumor immunity. Indeed, CD40 signaling in DCs has been demonstrated to be critical for both CD8+ and CD4+ T cell effector priming and activation and to be a prerequisite for tumor rejection in syngeneic tumor models[10].

Employing agonistic anti-CD40 antibodies to mimic this activation of APCs for tumor antigen presentation has long been recognized as an attractive strategy in the context of cancer immunotherapy. In particular, the ability of anti-CD40 molecules to activate DCs and subsequently increase (cross-)priming of tumor-specific T cells[11, 12] has fueled the notion that CD40 agonists could be crucial combination partners for eliciting long-lasting and sustainable antitumor responses[13, 14, 15]. A number of agonistic anti-CD40 antibodies have been developed over the last 20 years, of which several have undergone clinical evaluation in cancer patients[16, 17, 18, 19, 20, 21]. Although some of the tested antibodies triggered clinical responses, a major challenge to anti-CD40 agonist treatments has been the occurrence of dose-limiting side effects such as cytokine release syndrome (CRS) and hepatotoxicity[18, 19, 20, 21] resulting from systemic activation of CD40 at extratumoral sites.

In contrast, preclinical studies employing intratumoral or peritumoral injection of CD40-activating agents demonstrated that a stimulation of CD40 limited to the tumor area is well tolerated and induces effective antitumor responses. Importantly, despite the confinement of CD40 agonism to a primary tumor, systemic tumor-specific T cell responses were raised which enabled the eradication of secondary distant tumors and protected from tumor re-challenge[22, 23]. Together, these findings provide a solid rationale for targeting anti-CD40 agonism to the tumor in order to increase efficacy and reduce toxicity.

To this end, a tri-specific DARPin® molecule, MP0317, has been developed as a tumor-targeted anti-CD40 agonistic agent for systemic administration. MP0317 binds to CD40, fibroblast activating protein (FAP) and human serum albumin (HSA). Tumor targeting of MP0317 is achieved by the binding to FAP, a serine protease that is highly expressed on cancer-associated fibroblasts in the tumor microenvironment[24, 25] of a majority of carcinomas while only low amounts are expressed in a few healthy adult tissues such as lymph nodes[26, 27]. A report of a preferential localization of APC niches within the stromal area of human tumors[28] might provide further support for the potential of

FAP-targeted CD40 agonism in tumors. A key feature of the activity of MP0317 is that the binding of MP0317 to CD40 only induces activation when there is concomitant binding to FAP and when the FAP density is high enough to bring about clustering of CD40. In the absence of FAP or the presence of low density FAP, MP0317 binding to CD40 is unable to produce activation, thus systemic activation and resultant toxicity is avoided. Finally, the binding of MP0317 to HSA gives the molecule an extended half-life in the circulation.

In summary, the benefits of FAP-targeted CD40 agonism by MP0317 demonstrated in preclinical studies indicate the possibility of exploiting the full potential of CD40 signaling for cancer immunotherapy. Based on this, the current plan for exploring this potential involves a monotherapy phase 1 study in tumor types known to have high levels of FAP expression followed by exploration of combination studies as, based on published preclinical and clinical data, CD40 agonists have their greatest potential in combination therapies, for example with immune cell activating agents, chemotherapy[29, 30] and radiotherapy.

[redacted]

[redacted]

[redacted]

[redacted]

[redacted]

[redacted]

[redacted]

CONFIDENTIAL
May not be used, divulged, published, or otherwise disclosed without the consent of
Molecular Partners AG, Schlieren, Switzerland

[redacted]

[redacted]

[redacted]

[redacted]

[redacted]

[redacted]

[redacted]

[redacted]

[redacted]

1.3 Rationale

[redacted]

CONFIDENTIAL
May not be used, divulged, published, or otherwise disclosed without the consent of
Molecular Partners AG, Schlieren, Switzerland

[redacted]

[redacted]

[redacted]

[redacted]

[redacted]

[redacted]

CONFIDENTIAL
May not be used, divulged, published, or otherwise disclosed without the consent of
Molecular Partners AG, Schlieren, Switzerland

[redacted]

CONFIDENTIAL
May not be used, divulged, published, or otherwise disclosed without the consent of
Molecular Partners AG, Schlieren, Switzerland

[redacted]

1.3.2 Study Rationale and Benefit Risk Assessment

There is a clear need for a significant improvement to cancer immunotherapy. Introduction of immune checkpoint inhibitors to cancer therapy has demonstrated the potential of enhancing antitumor immunity to improve patient outcome but has also shown that checkpoint inhibition alone is ineffective in many cases. CD40 agonism has potential to improve cancer immunotherapy but agonist molecules evaluated to-date have had disappointing efficacy due to extratumoral CD40 activation and resultant dose-limiting peripheral immunotoxicities. In the Sponsor's opinion, this could be overcome by strict targeting of CD40 agonistic activity to the tumor – as intended with MP0317. Due to its unique mode of tumor-localized activation of CD40, MP0317 could offer such an improvement without causing side effects attributable to systemic immune cell activation, for example cytokine release and liver toxicity, seen with untargeted mAb CD40 agonists in clinical development. The tumor types selected for the proposed phase 1 monotherapy study represent good benefit/risk potential for clinical study explorations. Firstly, the advanced solid tumor types are known to express medium to high levels of FAP[24, 38] and secondly, study patients with the selected tumor types are those for whom approved therapies have been exhausted or who are ineligible or unable to tolerate other treatments.

Future clinical development will expand to include combination with other treatment modalities (e.g. checkpoint inhibitors, chemotherapy, radiotherapy). The selection of indications, combination partners and lines of therapy will be driven by biologic rationales, regulatory standards and state-of-art treatment and will be further defined as clinical data from MP0317 emerge. Based on non-clinical studies, it is thought that the FAP-targeted CD40 agonist MP0317 could induce tumor-localized generation of an inflammatory milieu capable of activating a humoral and cell-mediated antitumor immunity which could improve antitumor therapies, including immunotherapy, radiotherapy and chemotherapy without causing peripheral immunotoxicity.

Due to the expected markedly higher level of FAP in tumors than in healthy tissues, the FAP-targeted CD40 agonist MP0317 is expected to have a better safety profile, in particular with respect to CRS and liver toxicity, than the untargeted CD40 agonist mAbs that are currently in clinical development. Nevertheless, in the event that FAP expression at an extratumoral site is sufficient for CD40 activation, adverse effects associated with enhancement of an ongoing immune cell-mediated

inflammatory response could be possible. Otherwise, no activation or exacerbation of immune cell activity is expected at sites where there is low level or no FAP because CD40 activation by MP0317 is highly FAP-level-dependent. Weak off-target binding of MP0317 to Ly6H has been detected in an *in vitro* assay but no evidence of pharmacodynamic effects has been observed *in vivo*. Nevertheless, Investigators should be alert to the possibility that neurologic effects might be observed in patients in the FIH study.

Additionally, as MP0317 is a biological agent, there is a potential risk of IRRs occurring. Consequently, guidance for IRR management is provided in Appendix 12.6.

With respect to additional unknown risks, as is normal practice for phase 1, FIH studies, patients will be closely monitored to detect any additional study drug-specific adverse events (AEs).

Taking these potential benefits and risks into consideration, the Sponsor is of the opinion that the potential benefits of MP0317 for patients with advanced cancer outweigh the potential risks.

1.3.3 Rationale for DLT Evaluation Period

To allow for adequate assessment of the nature and incidence of, as well as recovery from, acute and delayed immune toxicities related to MP0317, the DLT evaluation period lasts for four weeks after the first study drug administration.

1.3.4 Rationale for Tissue and Blood Biomarker Sample Collection

The goal of the biomarker assessments is to confirm the mode of action (MOA) of MP0317, characterize pharmacodynamic effects and duration of pharmacodynamic response, evaluate the relationship between MP0317 exposure and pharmacodynamic biomarkers and identify predictors of safety or antitumor activity following MP0317 administration(s).

MP0317 treatment may result in changes of peripheral blood immune cells and soluble circulating markers. Therefore, plasma and serum samples will be collected and analyzed for soluble factor changes (including, but not limited to, soluble CD40 [sCD40], soluble FAP [sFAP], IL-12p70, IFN-γ, TNF-α etc.) and markers in tumor tissue (including, but not limited to, FAP, CD40, CD8+, DCs etc.) that may be associated with benefit from treatment. Blood samples will also be collected and analyzed with respect to alterations in the number and activation/differentiation of immune cells (including, but not limited to, CD40, CD8+, CD20, CD14 etc.) following treatment with MP0317.

Administration of therapeutic proteins can be associated with IRRs and CRS. Plasma or serum samples will be collected in the event of IRR or CRS for exploratory analysis to understand the underlying mechanism, by assessment of cytokines, such as IFN-γ, TNF-α, inflammation markers etc.

Moreover, treatment with MP0317 is expected to result in alterations of intratumoral residing immune cells and in particular activation of DCs, macrophages, B cells and T cells. Therefore, tumor samples will be collected from patients included in the dose-escalation part of the study. Mandatory tumor biopsies will be collected on two occasions, at screening and on-treatment, if clinically feasible (Section 7.1). If preliminary data suggest that modification of the on-treatment tumor biopsy time point would be more appropriate, alternative on-treatment tumor biopsy time points may be considered in future cohorts. Optional biopsies may be taken at progressive disease (PD) or response if the patient consents to these samples being taken to aid the understanding of immune resistance mechanisms.

Upon treatment, intratumoral immune cell changes are expected with respect to density of different immune cell lineages (including, but not limited to DCs, CD4+ T cells, CD8+ T cells, B cells, NK

cells and macrophages), activation/differentiation status as well as their location (including, but not limited to, MP0317, CD40, FAP, CD68 etc.). Such changes will be determined by immunofluorescence (IF) and gene expression analysis methods.

Preclinical studies suggest a certain threshold of cell-surface FAP expression is required to enable MP0317 crosslinking of surface CD40 leading to activation of target immune cells. Thus, the assessment and quantification of FAP in tumor specimens may correlate with responses to MP0317 treatment and allow for pre-selection of patients likely to benefit from treatment with this agent in future clinical studies. In addition to tumor biopsies, archival paraffin-embedded tumor tissue will be used to assess FAP expression and to investigate changes in the assessed markers over time compared to the mandatory pre-treatment baseline biopsy.

To help understand potential on-target off-tumor safety risks of MP0317 treatment linked to the FAP outside the tumor, tissue biopsies from wounded skin will be obtained from all participants. Surrogate pharmacodynamic markers will be explored pre and on-treatment to allow evaluation of the MP0317 effect on reactive fibroblasts expressing FAP and CD40 and on activated macrophages expressing CD40 involved in the process of skin repair. A potential MP0317 dose-dependent effect on the cell populations of interest that are present in the wound will also be explored (e.g. neutrophil recruitment, macrophage and immune cell infiltration).

Finally, all specimens will also be used for research purposes to identify additional biomarkers that may be useful to predict and monitor response to MP0317 treatment and safety, assess pharmacodynamic effects of MP0317 treatment and investigate any mechanisms of resistance to therapy. Additional markers may be measured in the case that a strong scientific rationale develops. Whole blood, serum/plasma and tissue samples will be collected at time points specified in Section 7.1.

CONFIDENTIAL
May not be used, divulged, published, or otherwise disclosed without the consent of
Molecular Partners AG, Schlieren, Switzerland

2 STUDY OBJECTIVES AND ENDPOINTS

2.1 Objectives

2.1.1 Primary

- To determine the recommended dose for expansion (RDE) or the maximum tolerated dose (MTD) for MP0317 as monotherapy in patients with advanced solid tumors (dose-escalation part only)
- To characterize the safety and tolerability of MP0317 as monotherapy in patients with advanced solid tumors

2.1.2 Secondary

- To describe the PK of MP0317 as monotherapy in patients with advanced solid tumors
- To evaluate preliminary antitumor activity of MP0317 as monotherapy in patients with advanced solid tumors
- To evaluate preliminary clinical benefit of MP0317 as monotherapy in patients with advanced solid tumors

2.1.3 Exploratory

- To evaluate pharmacodynamic effects of MP0317 as monotherapy in peripheral blood and tissue in patients with advanced solid tumors
- To evaluate the immunogenicity of MP0317 as monotherapy in patients with advanced solid tumors

2.2 Endpoints

2.2.1 Primary

- Incidence of DLTs
- Type, incidence and severity of AEs and serious adverse events (SAEs) according to the National Cancer Institute Common Terminology Criteria for Adverse Events (NCI CTCAE) v5.0
- Changes between screening and post-screening laboratory parameters and vital signs

2.2.2 Secondary

- Serum concentration-time profiles following first and repeated MP0317 infusions
- Determination of PK parameters including (but not limited to) C_{max}, time at C_{max} (T_{max}), minimal serum concentration (C_{min}), AUC, total clearance (CL), volume of distribution at steady state (V_{ss}) and half-life ($t_{1/2}$)
- Overall response rate (ORR) based on best overall response (BOR) of complete response (CR) and partial response (PR) locally assessed using Response Evaluation Criteria in Solid Tumors (RECIST) v1.1 and immunotherapy RECIST (iRECIST)

- Disease control rate (DCR) of CR, PR or SD lasting 4 or more weeks following the initiation of MP0317
- Duration of response (DOR) of CR or PR based on RECIST v1.1 and iRECIST, time to progression (TTP) following the initiation of MP0317
- Progression-free survival (PFS) based on RECIST v1.1 and iRECIST
- Overall survival (OS)

2.2.3 Exploratory

- Changes in frequency and functionality of B cells, DC, macrophages and T cell subsets in peripheral blood and tissue biopsies
- Assess FAP and CD40 expression and co-localization with MP0317 in tissue biopsies
- Changes in cytokines in serum
- Changes in sFAP and sCD40 in serum
- Circulating tumor DNA (ctDNA) (safety expansion part only)
- Occurrence of ADAs
- Incidence, titer and time-course of ADAs

CONFIDENTIAL
May not be used, divulged, published, or otherwise disclosed without the consent of
Molecular Partners AG, Schlieren, Switzerland

3 STUDY DESIGN

3.1 Description of the Study

This is a phase 1, FIH, multicenter, open-label, dose-escalation study followed by a safety expansion part, evaluating the safety, tolerability, PK, pharmacodynamics and preliminary antitumor activity of MP0317 in adult patients with advanced solid tumors. See Sections 1.3.1 and 1.3.3 for the rationale behind the dose, regimen and DLT evaluation period.

The dose-escalation part is designed to determine the RDE or the MTD for MP0317 monotherapy. The safety expansion part is designed to confirm safety in a larger population (Figure 2). The dose-escalation scheme will use an adaptive study design following a Bayesian Logistic Regression Model (BLRM). A dose-escalation review committee (DERC) will monitor safety and govern all cohort dosing decisions (Sections 5.2.7 and 8.5).

The sponsor in consultation with the DERC may advise on the opening of cohorts with alternative dosing schedules (e.g. q1w). Such additional cohorts may be opened concurrently with the initial q3w dosing schedule cohorts, during dose escalation as well as during safety expansion phase.

Once the RDE (or MTD) has been determined for each schedule, the safety expansion cohorts will be opened and up to 15 additional patients in each cohort will be treated with MP0317 monotherapy at this dose.

The first doses between the first 2 patients in any cohort must be separated by a minimum of 7 days.

Study treatment will be administered as described in Sections 7.3.1 and 5.2.2.

Paired (pre and on-treatment) tumor and skin biopsies will be mandatory for all patients. Biomarkers that may potentially correlate with antitumor activity or immunomodulatory effects of MP0317 may be explored during the study.

Figure 2. Study Design

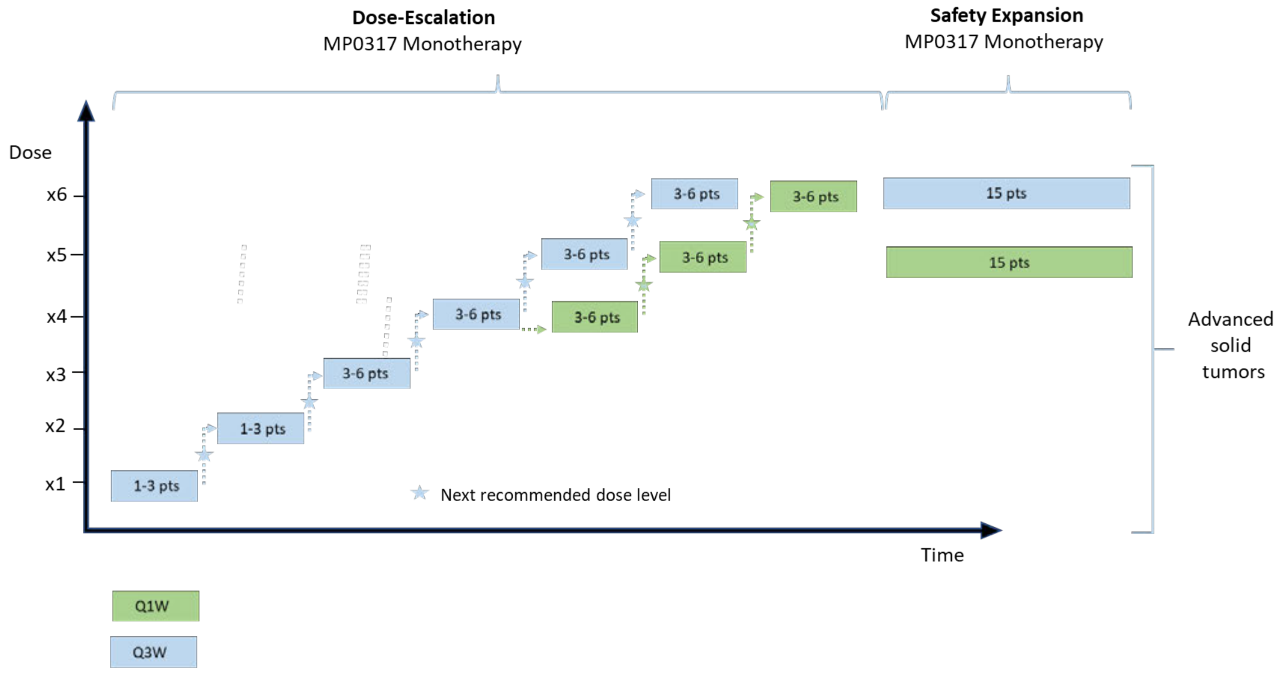

Pts, patients; Q1W, every week dosing schedule; Q3W, every 3 weeks dosing schedule.

3.2 Number of Patients and Study Sites

A total of between 32 and 78 patients are anticipated, 17 to 30 during the dose-escalation part for the q3w schedule, 12 to 18 for the q1w schedule and 15 during the safety expansion parts for each schedule chosen for expansion.

The study will be conducted in France and the Netherlands, in 2 sites in each country. Additional sites and countries may be considered during the study.

3.3 Duration of Study

For individual patients, the screening period starts at the informed consent date, up to 28 days before the first study drug administration. Study treatment will be administered until the decision is taken for the patient to discontinue from treatment for any of the events described in Section 7.3.4 (i.e., end of treatment [EOT]).

A safety follow-up (FU) visit will be scheduled at Week 4 after the last study drug administration (Section 7.3.2). Completion of safety FU or discontinuation from the study for any reason defines patient end of study (EOS). Thereafter, survival FU will be performed to collect survival status approximately 3 and 6 months after patient EOS or until withdrawal of consent, patient is lost to follow-up, death or study termination by the Sponsor, whichever occurs first (Section 7.3.3).

The overall study duration is from the time of recruitment of the first patient until the overall EOS (Section 3.4). The duration of the study will depend on safety and efficacy events encountered during the dose-escalation and the safety expansion parts and the rate of patient accrual.

Estimated time from when the study opens to enrollment until completion of data analyses is Q3 2021 to Q3 2023.

3.4 End of Study

The overall EOS is defined as the date when the last patient/last visit occurs or when the last data point required for statistical analysis or safety FU is received from the last patient, whichever occurs later.

In addition, the Sponsor has the right to terminate or suspend the entire study or dosing schedules thereof, or close a given study site at any time for any reason (Sections 11.3 and 11.4). Such a decision will be communicated to the Investigators in writing.

3.5 Blinding and Randomization

This is an open-label, non-randomized study. Neither blinding nor randomization techniques will be used in this protocol. Patients, Investigators, site staff and persons performing the assessments and data analysts as well as the Sponsor will know the identity of the treatment throughout the study.

CONFIDENTIAL
May not be used, divulged, published, or otherwise disclosed without the consent of
Molecular Partners AG, Schlieren, Switzerland

4 STUDY POPULATION

4.1 Patient Population

Patients who meet the inclusion and none of the exclusion criteria in Sections 4.2 and 4.3 will be eligible for participation in this study.

4.2 Patient Inclusion Criteria

A patient is eligible to be included in the study if they meet all of the following criteria:

1. Has an advanced, histologically-proven solid tumor of one of the following types, and for which approved therapies have been exhausted or for which the Investigator considers the patient ineligible or unable to tolerate other treatments:
    a. Colorectal cancer
    b. Ovarian cancer
    c. Endometrial cancer
    d. Gastric cancer
    e. Pancreatic cancer
    f. Anal cancer
    g. Cervical cancer
    h. Head and neck squamous cell carcinoma (HNSCC)
    i. Mesothelioma
    j. Prostate cancer
    k. Non-small cell lung cancer (NSCLC)
    l. Melanoma
    m. Urothelial/bladder cancer
    n. Microsatellite instability high cancer of any type
    o. Cutaneous squamous cell cancer
    p. Breast cancer
2. ≥ 18 years of age on the day of signing informed consent
3. Has signed and dated written informed consent before performing any study procedure, including screening
4. Eastern Cooperative Oncology Group (ECOG) performance status (PS) 0 to 1
5. Anticipated life expectancy ≥ 12 weeks by Investigator judgement
6. Measurable disease according to Response Evaluation Criteria in Solid Tumors (RECIST) v1.1
7. Should agree to undergo mandatory paired (pre and on-treatment) tumor biopsies and be considered to have biopsiable disease. The biopsies should be performed as follows:
    a. At least 1 tumor lesion ≥ 20 mm amenable to percutaneous biopsy other than the target lesion(s) used to follow response as defined by RECIST v1.1.
    b. For cutaneous or subcutaneous lesions, tumors should be ≥ 5 mm in diameter amenable to biopsy by excisional or punch biopsies without unacceptable risk of a major procedural complication.

CONFIDENTIAL
May not be used, divulged, published, or otherwise disclosed without the consent of
Molecular Partners AG, Schlieren, Switzerland

   c. For core needle biopsy specimens, at least 3 to 6 cores with an 18-gauge needle should be collected.
   d. The on-treatment tumor biopsy should be taken from the same lesion as the pre-treatment biopsy. The biopsied lesion should be large enough to take both biopsies ≥ 1 cm apart.
8. Should agree to undergo mandatory paired (pre and on-treatment) skin biopsies
9. At least 28 days must have elapsed between any prior major surgery and screening. The following procedures are not considered major:
   a. Obtaining the pre-treatment tumor and skin biopsies as per protocol requirements
   b. Placement of a port for central venous access
   c. Needle, punch or excisional biopsy of a clinically or radiographically detected lesion
10. Laboratory parameters at screening:
    a. Hematology:
       i. Platelet count ≥ 100,000 cells/mm^3
       ii. Absolute neutrophil count ≥ 1,000 cells/mm^3
       iii. Hemoglobin ≥ 9 g/dL
    b. Serum creatinine < 1.5 x upper limit of normal (ULN) or creatinine clearance > 50 mL/min on the basis of Cockcroft-Gault glomerular filtration rate estimation
    c. Coagulation:
       i. International normalized ratio (INR) < 1.5
       ii. Prothrombin time (PT) and activated partial thromboplastin time (aPTT) ≤ 1.5 x ULN unless therapeutically warranted
    d. Aspartate aminotransferase (AST) and alanine aminotransferase (ALT) < 3 x ULN
    e. Bilirubin normal, except for patients with known familial hyperbilirubinemia (such as Gilbert syndrome); for patients with documented Gilbert's syndrome (Gilbert-Meulengracht syndrome) total bilirubin ≤ 3 x ULN is acceptable
    f. Albumin > 2.8 g/dL or > 28 g/L, and without albumin transfusion for ≥ 7 days before screening
11. Is using highly effective contraception, for females of childbearing potential (FCBP) and for men, as follows and as defined in Appendix 12.2:
    a. <u>Female:</u> Is not pregnant, is not breastfeeding, and one of the following applies:
       - Not a FCBP
       - A FCBP who agrees and/or whose male partner agrees to follow the contraceptive guidance from screening, during the treatment period, and for at least 3 months after the last study drug administration. A FCBP must have a negative serum pregnancy test result at screening.
    b. <u>Male:</u> Agreement to use a highly effective contraception method from screening, during the treatment period, and for at least 3 months after the last study drug administration and to refrain from donating sperm during this period.

4.3 Patient Exclusion Criteria

A patient will be ineligible if one or more of the following statements are applicable:

28. Known hypersensitivity to excipients used in the MP0317 formulation
29. Autoimmune diseases, except autoimmune endocrinopathies that are stable with hormone replacement therapy

CONFIDENTIAL
May not be used, divulged, published, or otherwise disclosed without the consent of
Molecular Partners AG, Schlieren, Switzerland

30. Inflammatory diseases such as arthritis, colitis, liver fibrosis, cirrhosis, interstitial fibrosis or chronic obstructive pulmonary disease (COPD) that may have elevated tissue fibroblast activation protein (FAP) expression unless approved after consultation with the Sponsor
31. Serious illness or concomitant non-oncological disease considered by the Investigator to be incompatible with participating in the protocol
32. Left ventricular ejection fraction of < 50% on echocardiographic exam or multi-gated acquisition (MUGA) scan at screening
33. History or evidence of clinically significant cardiovascular disease defined as at least one of the following criteria:
    a. Evidence of poorly controlled arterial hypertension (systolic blood pressure > 160 mmHg or diastolic blood pressure > 100 mmHg)
    b. Myocardial infarction or instable angina pectoris within 6 months before screening
    c. Heart failure (New York Heart Association Class III or IV)
    d. Any cardiac arrhythmia that is not well controlled
    e. QT corrected (QTc) prolongation ≥ Grade 2 (> 480 ms) at screening measured on 2 separate electrocardiograms (ECG) at least 10 minutes apart
    f. Clinically significant valvular heart disease
34. Severe dyspnea, pulmonary dysfunction or need for continuous supportive oxygen inhalation
35. Arterial thromboembolic event, stroke or transient ischemia attack within 12 months before screening
36. Known central CNS metastases that are either untreated or are treated but are associated with clinical symptoms (e.g. headache, convulsions); patients with CNS metastases that have been treated with radiotherapy and/or surgery are eligible if they are clinically without symptoms for at least 6 weeks before screening; if under treatment with corticosteroids (not exceeding 10 mg/day prednisone or equivalent) and/or anticonvulsive agents, patients must be on a stable dose for at least 14 days before first study drug administration.
37. Active uncontrolled bleeding or a bleeding diathesis
38. Therapy for active infection needs to be completed at least 7 days before first study drug administration
39. Known positivity for human immunodeficiency virus (HIV) or history of HIV (HIV testing is not mandatory)
40. Active hepatitis B (chronic or acute; HBV) defined as having a positive hepatitis B surface antigen (HBsAg) test at screening. Patients with past or resolved HBV infection (defined as having a negative HBsAg test and a positive hepatitis B core antigen antibody test) are eligible.
41. Active hepatitis C (HCV) infection defined as having a positive HCV antibody test followed by a positive HCV ribonucleic acid (RNA) test at screening. The HCV RNA test will be performed only for patients who have a positive HCV antibody test. Patients who are positive for HCV antibodies are eligible only if polymerase chain reaction (PCR) is negative for HCV RNA.
42. Serious or non-healing wound, skin ulcer or non-healing bone fracture
43. Abdominal fistula, gastrointestinal perforation or intra-abdominal abscess within 6 months before screening
44. Any vaccine within 28 days before first study drug administration (clarification in Section 6.5)
45. An allogenic tissue/solid organ transplant
46. History of another primary malignancy except for:

CONFIDENTIAL
May not be used, divulged, published, or otherwise disclosed without the consent of
Molecular Partners AG, Schlieren, Switzerland

   a. Malignancy treated with curative intent and with no known active disease ≥ 2 years before screening and of relatively low potential risk for recurrence
   b. Adequately treated non-melanoma skin cancer or lentigo maligna without evidence of residual disease
   c. Adequately treated carcinoma in situ without evidence of disease
   d. Cancer patients with incidental histologic findings of prostate cancer that, in the opinion of the Investigator, is not deemed to require active therapy (e.g. incidental prostate cancer identified following cystoprostatectomy that is tumor/node/metastasis Stage ≤ pT2N0) may be eligible, pending discussion and approval by the Sponsor

47. Previous treatment with a DARPin® molecule
48. Concurrent enrollment in another clinical study, unless it is an observational (non-interventional) clinical study or it is the follow-up period of an interventional study
49. Use of an investigational agent within 28 days before first study drug administration
50. Any anticancer treatment, including chemotherapy, hormonal therapy or radiotherapy, within 21 days before first study drug administration; however, the following are allowed:
    a. Hormonal therapy with gonadotropin-releasing hormone (GnRH) agonists or antagonists
    b. Hormone-replacement therapy or oral contraceptives
    c. Palliative radiotherapy for bone metastases within 14 days before first study drug administration
51. Continuous corticosteroid use exceeding 10 mg/day prednisone or equivalent
52. Any condition that, in the opinion of the Investigator, would interfere with evaluation of the investigational medicinal product (IMP) or interpretation of the patient’s data
53. Unable or unwilling to comply with all study requirements for clinical visits, examinations, tests and procedures
54. Patient deprived of liberty by a judicial or administrative decision, patient admitted to a social institution or who is under a measure of legal protection, patient hospitalized without consent or who is in an emergency situation

CONFIDENTIAL
May not be used, divulged, published, or otherwise disclosed without the consent of
Molecular Partners AG, Schlieren, Switzerland

5 STUDY TREATMENT AND PROCEDURES

5.1 Study Drug Description, Preparation and Dispensing

5.1.1 Product Description

MP0317 is a recombinant antibody mimetic protein (designed ankyrin repeat protein or DARPin®). It is supplied by Molecular Partners AG.

Dosage Formulation: Concentrate solution for IV administration.

[redacted]

Route of Administration: IV infusion is performed via peripheral or central venous line. Detailed guidance and information on the use and administration of study treatment MP0317 is provided in the 'Instructions for Preparation and Administration of MP0317 IV Infusion' manual (hereinafter referred to as 'IMP Manual').

5.1.2 Study Treatment Preparation

Detailed guidance and information for the use and administration of MP0317 IMP to patients are provided in the IMP Manual. [redacted]

MP0317 doses are calculated based on body weight. Body weight should be assessed according to the Schedule of Assessment (Table 7 and Table 14). Body weight measured on the day of a dosing visit should be used for dose calculation. If an infusion bag needs to be prepared one day prior to the dosing visit, body weight measured at the previous visit should be used.

5.1.3 Study Treatment Handling and Storage

[redacted]

The Investigator or designee will be responsible for handling the preparation of the study drug of the appropriate doses to be administered, and completion of study-specific drug accountability logs. All study drug must be stored in a secure, environmentally controlled, and monitored area (manually or automated) in accordance with specified storage conditions, with access limited to the Investigator and authorized site staff.

Only patients participating in the study may receive study treatment and only authorized site staff may supply or administer study treatment.

CONFIDENTIAL
May not be used, divulged, published, or otherwise disclosed without the consent of
Molecular Partners AG, Schlieren, Switzerland

5.1.4 Study Treatment Compliance, Accountability and Destruction

All study drug must be received at the study site by a trained designated person, and handled and stored safely and properly and kept in a secure location with limited access restricted to authorized personnel only. The Investigator or designee must maintain current and accurate records of the receipt (documentation from shipments of study drugs received), administration (patient-by-patient and overall accounting) and disposal/destruction of study drug.

A clinical research associate (CRA) or designee will be responsible for monitoring the sites' study drug accountability. The CRA is the point of contact for any questions concerning administration of study drug. Records of study drug accountability, storage and handling must be made available to the CRA for the purposes of study drug accountability. Any discrepancy and/or deficiency must be recorded with an explanation.

Instructions for the disposal of unused study treatment will be provided in the IMP Manual.

Investigators and other study staff should refer to the IMP Manual for detailed instructions on study drug.

Study drug may not be used for any purpose other than that described in the protocol.

5.2 Study Treatment Schedule and Administration

5.2.1 Study Drug Dose

For each patient, the assigned dose of MP0317 will be based on the available cohort as recommended by the DERC. For the dose-escalation study part on a q3w schedule, the starting dose is 0.03 mg/kg q3w and up to 6 q3w dose levels are planned, as shown in Table 3. The doses to be evaluated on a q1w schedule in the dose-escalation part are shown in Table 3. A treatment cycle will be 3 weeks (21 days), with DLT evaluation period of 4 weeks after first study drug administration. See Section 5.2.9 for study treatment modifications.

[redacted]

[redacted]

[redacted]

[redacted]

5.2.2 Study Drug Administration

The IV infusion must be administered according to standard clinical procedures, under the supervision of an Investigator or designee. [redacted]

Patients who receive the study treatment will remain under medical supervision for at least 24 hours after the end of their first and second study drug administrations. After the end of their third to fifth infusions, patients should remain under medical supervision for at least 4 h, and from the sixth infusion onwards for at least 1 h.

5.2.3 Initiation of a New Study Drug Administration

Study drug must only be administered to eligible patients. Patient suitability for receiving study treatment is decided before each administration by the Investigator, based on protocol eligibility criteria for the initial administration (Section 4) and on scheduled pre-infusion safety assessments for subsequent administrations (Sections 7.4.11).

5.2.4 Treatment Period

Study treatment will be administered as described in Sections 7.3.1 and 5.2.2.

5.2.5 Dose-Limiting Toxicities

DLT evaluation and a recommendation regarding dose-escalation steps will be made by the DERC (Section 8.5), after consideration of all available safety information.

A DLT is defined as an AE or abnormal laboratory result meeting any of the criteria below, that is assessed by the Investigator as related to study treatment, and that occurs within the DLT evaluation period, including death (Section 5.2.6). Severity of all AEs will be assessed according to NCI CTCAE v5.0.

- Any ≥ Grade 3 study drug-related non-hematologic AE, including the following abnormal laboratory results: Hepatic laboratory parameters potentially fulfilling Hy's Law criteria[39, 40]: AST or ALT ≥ 3 x ULN and concomitant total bilirubin ≥ 2 x ULN
- Any Grade 4 study drug-related hematologic AE or abnormal laboratory result (hematologic or non-hematologic)
- Grade 3 neutropenia lasting > 7 days despite adequate standard-of-care medication
- Grade 3 or 4 febrile neutropenia (fever ≥ 38.5 °C)
- Grade 3 anemia that cannot be adequately treated by blood transfusions
- Inability to administer the next dose due to a drug-related AE or delayed administration of the next dose due to a drug-related AE and/or abnormal laboratory result lasting > 14 days despite adequate medication
- Any other AE/abnormal laboratory result which, in the view of the Investigator and/or Sponsor, is deemed clinically significant and considered a DLT

Note that the following AEs/abnormal laboratory results, some of which are expected with the study treatment, will not be considered a DLT:

- Any transient Grade 3 or 4 abnormal laboratory results without any related clinical symptoms and lasting < 72 h
- Grade 3 events that have not been optimally treated with standard-of-care medication, lasting < 7 days
- Grade 4 amylase or lipase not associated with symptoms or clinical manifestations or radiologic signs of pancreatitis

CONFIDENTIAL
May not be used, divulged, published, or otherwise disclosed without the consent of
Molecular Partners AG, Schlieren, Switzerland

- Grade ≥ 3 lymphocytopenia not associated with symptoms or clinical manifestations
- Grade 3 hypertension resolving within 48 h
- Grade 3 IRR resolving under adequate treatment (first event only)
- Grade 3 fatigue lasting ≤ 14 days

An AE considered by the Investigator to be disease-related should not be considered a DLT.

DLTs which meet the criteria for an SAE or AE of special interest (AESI) should be reported according to the procedure outlined in Section 8.2.2. All DLTs must be reported within 24 h to the Sponsor and documented in the electronic case report form (eCRF) as a DLT.

5.2.6 Dose-Limiting Toxicity Evaluability

The evaluability rules for DLT assessment apply to dose-escalation cohorts only.

The DLT evaluation period is defined as the 4-week period after first study drug administration.

Patients evaluable for DLT must either a) receive at least 2 administrations of MP0317 at q3w dosing schedule or 3 administrations at q1w dosing schedule at the assigned dose level and be observed until pre-dose of Cycle 2 Day 8 or b) experience a DLT as defined in Section 5.2.5 during the DLT evaluation period.

A patient will <u>not</u> be considered as DLT evaluable and may be replaced if they experience any of the following events during the DLT evaluation period:

- Received an MP0317 dose which is less than 90% or more than 110% of the planned infusion dose.
- Discontinued from treatment due to AE or death unrelated to study treatment, withdrawal of consent, lost to follow-up or Investigator decision (unrelated to study treatment).

5.2.7 Dose-Escalation Decisions

5.2.7.1 Dose-Escalation

Dose-escalation of MP0317 will be guided by a BLRM[41] based on any DLTs observed during the DLT evaluation period as defined in Section 5.2.6. The BLRM is a well-established method to estimate the RDE/MTD in cancer patients. The adaptive BLRM will be guided by the Escalation With Overdose Control (EWOC) principle[41, 42] to control the risk of DLT in patients at escalated dose levels. This method allows the incorporation of available information and updates the model parameters based upon new data for any DLTs observed during the course of the study. The model's estimation of the probability of DLT and the recommendation of the next dose level will be performed at each escalation step and will utilize the entire history of all available data from previous cohorts. The model will be utilized to help decision-making by the DERC (Section 8.5).

Once the starting dose level of MP0317 is determined to be safe, it will be escalated per the provisional dose levels outlined in Table 3. Dose level -1 represents a dose that may be evaluated if dose level 1 is poorly tolerated. No dose de-escalation below this level is planned. If dose level -1 is poorly tolerated, the study will be terminated.

For the purposes of dose-escalation decisions, the first two cohorts will consist of 1 to 3 evaluable patients to assess safety and tolerability of the MP0317 starting dose. Based on the safety profile, additional patients may be included in these cohorts as needed. Each subsequent cohort will consist of 3 to 6 evaluable patients (Table 3). Dose-escalation decisions will be made once the cohort of

CONFIDENTIAL
May not be used, divulged, published, or otherwise disclosed without the consent of
Molecular Partners AG, Schlieren, Switzerland

patients has met the criteria defined in Section 5.2.6. If only two patients in a cohort are evaluable and neither experiences treatment-related AEs ≥ Grade 2 (NCI CTCAE v5.0), dose-escalation decisions may be considered. Q1w dosing schedule will be independent from q3w dosing schedule decision rules.

Based on the recommendation of the BLRM considering the highest dose that may not be exceeded at any decision point during escalation and the maximum dose increase allowed by the protocol, the DERC may advise exploration of an alternative dosing schedule (e.g. q1w) based on review of available clinical data. If a new schedule is explored the total planned dose intensity/exposure for the treatment cycle will not exceed that of a previously tolerated schedule, or a currently allowed escalation thereof, and the maximum daily dose will not exceed a 233% increase of a previously tolerated dose on any schedule. Additional higher and/or intermediate dose levels might be added to either schedule during the course of the study. Cohorts may be added at any dose level below the MTD to better understand the safety, PK and/or pharmacodynamic profile. Multiple dose levels below the MTD may be evaluated simultaneously to obtain PK and pharmacodynamic data across a range of doses and to establish the RDE/MTD.

There will be no intra-patient dose-escalation during the DLT evaluation period. However, after selection of the RDE, patients treated at a lower dose can be given the option to increase their dose to the RDE at the discretion of the Investigator and after consultation with the Sponsor.

Table 3. Provisional Dose Levels for Dose-Escalation given Q3W or Q1W

Cohort Number	Dose Level (mg/kg)	Dose schedule	Number of Patients	Dose Level Increment (cumulative dose in mg/kg)
-1 [a]	0.01	q3w	1 to 3	- 66%
1	0.03	q3w	1 to 3	Starting dose
2	0.10	q3w	1 to 3	233%
3	0.30	q3w	3 to 6	200%
4	1.00	q3w	3 to 6	233%
4b	0.50	q1w[b]	3 to 6	50% compared to 4
5	3.00	q3w	3 to 6	200% compared to 4
5b	1.50	q1w[b]	3 to 6	200% compared to 4b
6	10.00	q3w	3 to 6	233% compared to 5
6b	5.00	q1w[b]	3 to 6	233% compared to 5b

[a] Dose level -1 represents a dose that may be evaluated if dose level 1 is poorly tolerated. No dose de-escalation below this level is planned. If dose level -1 is poorly tolerated, the study will be terminated.

[b] Initiated after DERC recommendation on the advice of the sponsor.

5.2.7.2 MTD Definition

The MTD is defined as the highest dose of study drug where the posterior probability of the true DLT rate in the target interval [0.16 to 0.33] of the MTD is above 0.50 with less than 25% risk of the true DLT rate being above 33%, and at least 6 patients have been treated in a confirmatory cohort of patients for the duration of the DLT evaluation period (Section 5.2.6).

5.2.7.3 Estimation of the RDE/MTD

A two-parameter BLRM employing the EWOC principle will be used during the escalation part for selection of doses to investigate, and for estimation of the MTD. Estimation of the MTD will be based

CONFIDENTIAL
May not be used, divulged, published, or otherwise disclosed without the consent of
Molecular Partners AG, Schlieren, Switzerland

on the estimation of the probability of DLT during the DLT evaluation period in patients in the dose-determining set (DDS; Section 9.3.2). If the MTD is not reached during the dose-escalation part, the DERC will recommend the appropriate dose level as described above. The MTD of different dosing schedules will be determined independent from each other.

At all dose-escalation decision time points, the adaptive BLRM permits alterations in the dose increments based on the observed toxicities.

In general, dose-escalation will not exceed a 3.33-fold increase from the current dose being studied.

Dose-escalation, in either schedule (q1w, q3w), may be terminated at any time based on emerging safety concerns without establishing the MTD.

Based on emerging PK data, a dose that is considered acceptably safe after safety evaluation of the initial 3-6 patients (i.e. shown to be lower than any potential MTD) may be further expanded to better characterize the safety, PK and pharmacodynamic biomarkers at this dose level. Patients in the safety expansion part who do not have at least one post-screening efficacy assessment will be replaced.

5.2.8 Safety Waiting Period

The first doses between the first 2 patients in any cohort must be separated by a minimum of 7 days. No further safety waiting periods for patients on the same dose level are required, however, the DERC can increase the initial safety waiting period according to the nature of emerging safety data.

5.2.9 Treatment Modification (Delay, Interruption)

Guidelines for study treatment modifications are shown in Table 4.

The following should be taken into consideration:

- If supportive care is ineffective, drug administration delays or infusion interruptions should be considered to prevent worsening of the related AE(s).
- Drug administration delays or infusion interruptions may be implemented for Grade 1 related AEs if the Investigator feels it is in the interests of the patient's safety.
- Drug administration delay for any reason is allowed for up to 3 weeks. Re-starting treatment after a delay longer than 3 weeks may be allowed with approval of the Sponsor. During such delay, a limited set of study assessments/visits may be performed as determined by the Investigator in consultation with the Sponsor.
- Guidelines for the management of IRRs and CRS are provided in Appendix 12.6 and Appendix 12.7, respectively.
- Any treatment modifications such as drug administration delays or infusion interruptions should be entered in the eCRF.

Study treatment should be permanently discontinued for Grade 4 or recurrent Grade 3 AEs, unless otherwise specified in Table 4.

Table 4. Guidelines for Study Treatment Modifications

Immune-Related AE [a]	Severity (NCI CTCAE v5.0)	Treatment Modification
IRR (Appendix 12.6)	Grade 1 to 3	Appendix 12.6

Immune-Related AE [a]	Severity (NCI CTCAE v5.0)	Treatment Modification
	Grade 4 or recurrent Grade 3 (that cannot be adequately treated with standard-of-care)	Permanently discontinue
CRS (Appendix 12.7)	Grade 1 to 3	Withhold until clinical improvement of symptoms according to Investigator judgement. Re-start at 50% of infusion rate of the current infusion and increase gradually.
	Grade 4 or recurrent Grade 3 (that cannot be adequately treated with standard-of-care)	Permanently discontinue
Non-infectious pneumonitis	Grade 2	Withhold until recovered to Grade 0 to 1 [a]
	Grade 3 or 4 or recurrent Grade 2	Permanently discontinue
Non-infectious colitis	Grade 2 or 3	Withhold until recovered to Grade 0 to 1 [a]
	Grade 4 or recurrent Grade 3	Permanently discontinue
Non-infectious nephritis	Grade 2 with serum creatinine > 1.5 x ULN to ≤ 3 x ULN	Withhold until recovered to Grade 0 to 1 [a]
	≥ Grade 3 with serum creatinine > 3 x ULN	Permanently discontinue
Endocrinopathies	Symptomatic hypophysitis<br>Diabetes mellitus type 1 associated with ≥ Grade 3 hyperglycemia (glucose > 250 mg/dL or > 13.9 mmol/L) or associated with ketoacidosis<br>Hyperthyroidism ≥ Grade 3	Withhold until recovered to Grade 0 to 1 [a]<br>For patients with Grade 3 or 4 endocrinopathy that improved to Grade 2 or lower and is controlled with hormone replacement, if indicated, continuation of study treatment may be considered after corticosteroid taper, if needed. Otherwise treatment should be permanently discontinued.<br>Hypothyroidism may be managed with replacement therapy without study treatment interruption.
Non-infectious hepatitis	Grade 2 with AST or ALT ≥ 3 to 5 x ULN or total bilirubin > 1.5 to 3 x ULN	Withhold until recovered to Grade 0 to 1 [a]
	≥ Grade 3 with AST or ALT > 5 x ULN or total bilirubin > 3 x ULN	Permanently discontinue
	In case of liver metastasis with screening Grade 2 elevation of AST or ALT, hepatitis with AST or ALT increases ≥ 50% and lasts ≥ 7 days	Permanently discontinue

**Immune-Related AE [a]**	**Severity (NCI CTCAE v5.0)**	**Treatment Modification**
Skin reactions	Grade 3 or suspected Stevens-Johnson syndrome (SJS) or toxic epidermal necrolysis (TEN)	Withhold until recovered to Grade 0 to 1 [a]
	Grade 4 or confirmed SJS or TEN	Permanently discontinue
Other immune-related adverse reactions	Based on severity and type of reaction (Grade 2 or 3)	Withhold until recovered to Grade 0 to 1 [a]
	Grade 3 or 4 myocarditis<br>Grade 3 or 4 encephalitis<br>Grade 3 or 4 Guillain-Barré syndrome<br>Recurrent Grade 3 or Grade 4	Permanently discontinue
Tumor lysis syndrome	Grade 3 or 4	Permanently discontinue

[a] If an immune-related AE does not resolve to Grade 0 to 1 within 3 weeks after last study drug administration or if corticosteroid dosing cannot be reduced to ≤ 10 mg prednisone or equivalent per day within 3 weeks, study drug administration should be permanently discontinued.

ALT, alanine aminotransferase; AST, aspartate aminotransferase; NCI CTCAE, National Cancer Institute Common Terminology Criteria for Adverse Events; SJS, Stevens-Johnson syndrome; TEN, toxic epidermal necrolysis; ULN, upper limit of normal.

5.2.10 Time Windows for Administration of Study Treatment

If the study treatment cannot be administered on the scheduled day for reasons other than AE the following time windows are allowed for q3w dosing schedule (Table 5) and q1w dosing schedule (Table 6):

Table 5. Time Windows for Administration of Study Treatment (Q3W)

**Treatment Cycles**	**Allowed Time Window**
**For Dose-Escalation Cohorts**	
Between Cycle 1 and 2	21 + maximum 2 days
From Cycle 2 to Cycle 4	21 ± 2 days
From Cycle 5 onwards	21 ± 3 days
**For Safety Expansion Cohort**	
From Cycle 1 to Cycle 4	21 ± 2 days
From Cycle 5 onwards	21 ± 3 days

Table 6. Time Windows for Administration of Study Treatment (Q1W)

Treatment Cycles	Allowed Time Window
**For Dose-Escalation Cohorts**	
During DLT evaluation period	+ maximum 1 day
After DLT evaluation period	± 1 day
**For Safety Expansion Cohort**	
For all administration visits	± 1 day

Whenever possible the study treatment frequency should be respected.

CONFIDENTIAL
May not be used, divulged, published, or otherwise disclosed without the consent of
Molecular Partners AG, Schlieren, Switzerland

6 CONCOMITANT MEDICATION

Concomitant medication includes any medication (e.g. prescription drugs, over-the-counter drugs, herbal medication, homeopathic remedies, nutritional supplements) taken within 28 days before the first study drug administration and until 28 days after the last study drug administration or patient EOS.

The patient should report all such medication to the Investigator, and it should be recorded in the eCRF, including:

- Reason for use
- Dates of administration including start and end dates
- Dosage information including dose, frequency and route

The Sponsor should be contacted if there are any questions regarding concomitant medication, therapies or procedures.

6.1 Permitted Therapy for Chronic Diseases and Maintenance Therapy

Stable doses of medications administered for chronic diseases (e.g. hypertension) are allowed. The Investigator must discuss any concomitant therapy with the Sponsor on a case-by-case basis where there is a potential for it to interfere with MP0317 or to alter the interpretation of results.

Systemic corticosteroids and TNF-α inhibitors may attenuate potential beneficial immunologic effects of treatment with MP0317 but may be administered at the discretion of the Investigator after consultation with the Sponsor. If feasible, alternatives to corticosteroids should be considered. The use of inhaled corticosteroids and mineralocorticoids (e.g. fludrocortisone) for patients with orthostatic hypotension or adrenocortical insufficiency is allowed. Megastrol administered as an appetite stimulant is acceptable while the patient is participating in the study. Planned use of other medications should be discussed with the Sponsor.

Hormonal therapy with GnRH agonists or antagonists for prostate cancer, oral contraceptives, hormone-replacement therapy, prophylactic or therapeutic anticoagulation therapy (such as low molecular weight heparin or warfarin at a stable dose level) or other allowed maintenance therapy should be continued.

Males and females of reproductive potential must use highly effective means of contraception (Section 4.2 and Appendix 12.2).

6.2 Management of Infusion Related Reactions and Cytokine Release Syndrome

Guidelines for the management of IRRs and CRS are provided in Appendix 12.6 and Appendix 12.7, respectively.

6.3 Prophylactic Premedication

Prophylactic premedication to prevent IRRs may be administered at the discretion of the Investigator. During the second DERC meeting held on 9th March 2022, based on safety data from the first two q3w dosed cohorts, the DERC members strongly recommended prophylactic premedication of patients with antihistamine and paracetamol. Furthermore, based on safety information emerging

during the course of the study, the DERC may consider mandating prophylactic premedication to manage IRRs, e.g., by using the regimen detailed in Appendix 12.6.

6.4 Anticancer Treatment

Concomitant use of anticancer treatment other than MP0317 is specified in Section 4.3.

6.5 Vaccines

Any live vaccines are prohibited within 28 days before first study drug administration, during treatment and for 5 months following the last study drug administration.

Other vaccines (such as inactivated [e.g. seasonal flu], subunit, toxoid, or messenger RNA [mRNA] based [e.g. COVID-19] etc.) are prohibited within 28 days before first study drug administration and during the 4-week period after first study drug administration (i.e. patients can be vaccinated with these compounds starting from Cycle 2 Day 8). However, depending on a risk benefit assessment, individual patients could also be vaccinated within the prohibited time period after consultation with the Sponsor.

6.6 Herbal Therapies

Concomitant use of herbal therapies is not recommended given that their PK, safety profile and potential drug-drug interactions are generally unknown.

7 VISIT SCHEDULES AND ASSESSMENTS

7.1 Study Flow and Visit Schedule

7.1.1 Q3W Dosing Schedule

Table 7. Schedule of Assessments (Q3W)

	Screening	Treatment Period							Follow-up	
**Visit**		**Cycle 1**	**Cycle 2**	**Cycle 3**	**Cycle 4**	**Cycle 5**	**Cycle 6**	**Cycle 7 +**	**Safety FU** [c]	**Survival FU** [d]
Visit days [a]	Days -28 to -1	Day 1,2,5,8,11, 15,19	Day 1,8,15	Day 1,8,15	Day 1,8,15	Day 1,8,15	Day 1	Day 1		
**Assessment**										
Informed consent [e]	X									
Eligibility review [f]	X									
Medical history [g]	X									
Demography	X									
Cancer disease and treatment history [h]	X									
Echocardiography or MUGA scan	X		X (D1) [i]						X	
Physical examination [b, j]	X	X (D1)	X (D1,8)	X (D1)	X (D1)	X (D1)	X	X	X	
ECOG PS [j]	X	X (D1)	X (D1)	X (D1)	X (D1)	X (D1)	X	X	X	
Vital signs [b, k, bb]	X	X	X	X	X	X	X	X	X	
ECG [b, l]	X	X (D1,D8,D15) [l]	X [l]	X [l]	X [l]	X (D1) [l]	X [l]	X [l]	X	
Thyroid function [b, m]	X			X (D1)		X (D1)		X (Cycle 7 and every other cycle thereafter)	X	
Pulse oximetry [b, n]	X	X	X	X	X	X	X	X	X	
Urinalysis [b, o]	X	X (D1)	X (D1)	X (D1)	X (D1)	X (D1)	X	X	X	
Pregnancy test [b, p]	X	X (D1)	X (D1)	X (D1)	X (D1)	X (D1)	X	X	X	
Local laboratory safety assessments [b, q]	X	X (D1,D8,D15)	X	X	X	X	X	X	X	
Immunology [aa]	X	X (D1)	X (D1)	X (D1)	X (D1)	X (D1)	X	X	X	
Central laboratory assessments in the events of IRR or CRS [v]		(X)	(X)	(X)	(X)	(X)	(X)	(X)		
HBV, HCV test [r]	X									
PK [b, s]		X	X	X	X	X	X	X	X	
Immunogenicity (ADA) [b, s]		X (D1,D8,D15)	X	X (D1)	X (D1)	X (D1)	X	X	X	
Blood biomarkers [b, s]		X	X	X	X (D1,D8)	X (D1)			X	

	Screening	Treatment Period							Follow-up	
**Visit**		**Cycle 1**	**Cycle 2**	**Cycle 3**	**Cycle 4**	**Cycle 5**	**Cycle 6**	**Cycle 7 +**	**Safety FU** [c]	**Survival FU** [d]
Visit days [a]	Days -28 to -1	Day 1,2,5,8,11, 15,19	Day 1,8,15	Day 1,8,15	Day 1,8,15	Day 1,8,15	Day 1	Day 1		
Tumor assessment by CT/MRI [u]	X [t]		X [u]		X [u]			X (Cycle 7 and every 3rd cycle thereafter)	X [u]	
Tumor biopsy (archival) [w]	X									
Tumor biopsy (fresh), pre and on-treatment [b, x]	X		X (D8)							
Tumor biopsy (fresh), on PD/response (optional) [y]		(X)							(X)	
Skin biopsy [b]	X [cc]	X (D5,D8) [cc]								
Adverse events	X [dd]	X	X	X	X	X	X	X	X	X [ee]
Prior and concomitant medication	X	X	X	X	X	X	X	X	X	
MP0317 administration [z]		X (D1)	X (D1)	X (D1)	X (D1)	X (D1)	X	X		
Survival information										X

a **Visit days.** Visits are in calendar days, not business days.

b **Detailed schedule of assessments.** For assessment time points and/or allowed visit windows see Table 8 to Table 13.

c **Safety FU.** At Week 4 (± 1 week) after the last study drug administration (Section 7.3.2). All patients should complete the safety FU period after treatment discontinuation. If the safety FU visit cannot be performed (e.g. withdrawal of consent), the last visit will be considered the safety FU visit for this patient. If a subsequent anticancer treatment is initiated during the 28-day safety FU period, the safety FU visit should be before initiation of such therapy. If this is not possible, the safety (FU) visit will be done as soon as possible after initiation of such therapy.

d **Survival FU.** Survival FU will be performed after completion of the last safety FU visit or discontinuation from the study for any reason (i.e. patient EOS). Survival status will be collected by the study site via telephone contact, clinic visits and/or consultation of the patient medical records at approximately 3 (± 2 weeks) and 6 months (± 2 weeks) after patient EOS, or until withdrawal of consent, patient is lost to follow-up, death or study termination by the Sponsor, whichever occurs first (Section 7.3.3).

e **Informed consent.** Must be dated and signed before any study-specific procedure is performed (Section 11.1.2).

f **Eligibility review.** All inclusion/exclusion criteria must be reviewed during screening (Sections 4.2 and 4.3).

g **Medical history.** Including clinically significant diseases, surgeries, reproductive status and medication (including over-the-counter or prescription medicines, vitamins and/or herbal supplements) taken within 28 days before the first study drug administration will be recorded (Sections 6 and 7.4.1).

h **Cancer disease and treatment history.** Includes date(s) of diagnosis, classification by origin, tissue type, grade, stage, markers and mutations if available, prior anticancer treatments and procedures (Section 7.4.2).

i **Echocardiography or MUGA scan.** Measurements should follow European Society for Cardiology Guidelines 2016[43] and be performed pre-dose (Section 7.4.7).

j **Physical examination and ECOG PS.** Will be collected as per institutional standard practice and assessed according to ECOG criteria (Appendix 12.1). Physical examination needs to be done before each study drug administration and at Day 8 in Cycle 2 during dose escalation part. Abnormalities observed at screening are recorded in the medical history eCRF. At subsequent visits (or as clinically indicated), limited, symptom-directed physical examination should be performed. New or worsened clinically significant abnormalities occurring after screening should be recorded as AEs in the eCRF (Sections 7.4.3 and 8.1).

k **Vital signs.** Assessments include body weight, height (at screening only), body temperature, respiratory rate, pulse rate and blood pressure while the patient is in a supine or seated position and before any blood draws. Assessment of blood pressure and pulse rate will be preceded by at least 5 min rest for the patient in a quiet setting without distractions (e.g. television, mobile phone) (Section 7.4.4). Measurement of body weight will be made with the patient wearing indoor daytime clothing without shoes according to institutional standard practice.

l **ECG.** For each patient, ECGs should be obtained from the same machine whenever possible. Dose-escalation part: Screening, D1 pre-dose, D8 and D15 in Cycle 1 to Cycle 4. From Cycle 5 onwards at D1 pre-dose only. Safety expansion part: Screening and all cycles at D1 pre-dose (Section 7.4.6).

CONFIDENTIAL
May not be used, divulged, published, or otherwise disclosed without the consent of
Molecular Partners AG, Schlieren, Switzerland

[m] **Thyroid function.** TSH, free T3, free T4 will be monitored repeatedly throughout the study to detect any potential autoimmune thyroiditis (Section 7.4.8).

[n] **Pulse oximetry.** To detect presence of hypoxemia (e.g. from possible pneumonitis; Section 7.4.5).

[o] **Urinalysis.** Urine dipstick at screening, on Day 1 of each cycle before study drug administration, and at safety FU visit. If positive findings are noted and are thought to be of clinical significance, detailed urinalysis will be performed at a local laboratory (Section 7.4.9).

[p] **Pregnancy test.** Female patients of childbearing potential will have a serum pregnancy test at screening and a serum or urine pregnancy test before each study drug administration and at the safety FU visit (Sections 7.4.10 and 8.3).

[q] **Local laboratory safety assessments.** Parameters to be evaluated are detailed in Section 7.4.11. Values based on blood samples taken up to 48 h before scheduled study drug administration will be used by the Investigator to decide if the patient is suitable for study treatment before each administration (Section 5.2.3).

[r] **HBV, HCV test.** HBV test includes HBsAg. HCV test includes HCV antibody (if positive an HCV RNA test by PCR must be done; Section 7.4.11).

[s] **PK, immunogenicity (ADA) and blood biomarkers.** PK and immunogenicity (ADA) will be assessed using serum, and blood biomarkers (to evaluate disease status, safety or predictive biomarkers) will be assessed using serum, plasma and/or fresh blood. See Sections 7.4.13, 7.4.14 and 7.4.16.3 and refer to the Laboratory Manual.

[t] **Tumor assessments at screening.** If a tumor assessment (including CT scan according to RECIST v1.1) was performed within 28 days before screening it is not required to be repeated and can be used as screening assessment.

[u] **Tumor assessments.** Tumor measurements will be made at screening and every 6 to 8 weeks after the start of study treatment (within 7 days before scheduled infusion of Cycles 3, 5 and 8 then every third cycle and according to institutional standard practice) until PD, withdrawal of consent, death, or study termination, whichever occurs first. CT scans will be performed using institutional standard operating procedures. In specific cases, tumor measurements may be performed by MRI scans instead of (or in addition to) CT scans. However, the same imaging modality as used for screening should be used throughout the study (Section 7.4.15).

[v] **Central laboratory assessments in the events of IRR or CRS.** Cytokines, complement factors, drug-specific IgE and tryptase will be assessed in the events of IRR or CRS (Section 7.4.12). Sample should be taken 30-40 minutes within onset of reaction.

[w] **Tumor biopsy (archival).** If an archival tumor sample is available with an associated pathology report, performed at any time prior to study entry, this should be provided. However, only tissue from core needle, punch or excisional biopsy samples will be considered for study analyses, while fine needle aspiration, brushing and lavage samples are not acceptable. For all biopsy types, at least 5 to 15 unstained good quality slides should be provided or blocks that have sufficient tissue to generate at least 15 slides. Tissue for which the pathology report specifies that the overall tumor content is low (e.g. ‘sparse’ or ‘scant’) will not be considered.

[x] **Tumor biopsy (fresh), pre and on-treatment.** The biopsies should be performed as follows: a) At least 1 tumor lesion ≥ 20 mm amenable to percutaneous biopsy other than the target lesion(s) used to follow response as defined by RECIST v1.1. b) For cutaneous or subcutaneous lesions, tumors should be ≥ 5 mm in diameter amenable to biopsy by excisional or punch biopsies without unacceptable risk of a major procedural complication. c) For core needle biopsy specimens, at least 3 to 6 cores with an 18-gauge needle should be collected. d) The on-treatment tumor biopsy should be taken from the same lesion as the pre-treatment biopsy. The biopsied lesion should be large enough to take both biopsies ≥ 1 cm apart.

[y] **Tumor biopsy (fresh), on PD/response (optional).** Patients may agree to an additional tumor biopsy, preferably taken at the time of radiographic PD or response, that will be collected at the discretion of the Investigator and only if the patient is capable of providing it at the time requested.

[z] **MP0317 administration.** The MP0317 infusion solution must be administered completely and the entire infusion line flushed with 25 mL 0.9% NaCl. The infusion duration should be at least 50 min (excluding flushing) and no longer than 2 h (including flushing). Usage of a 0.2 µm in-line filter is mandatory. It is recommended to start infusions in the morning to allow adequate time for pre-infusion assessments, infusion, post-infusion medical supervision and PK assessments (Section 5.2.2). Body weight measured at Day 1 of each treatment cycle should be used for dose calculation. If an infusion bag needs to be prepared one day prior to the Day 1 visit of the respective cycle, body weight measured at the previous visit should be used (Section 5.1.2).

[aa] **Immunology.** Assess at the beginning of each cycle (parameters to be evaluated are detailed in Section 7.4.11).

[bb] **Height.** At screening only. Measurements will be made with the patient wearing indoor daytime clothing without shoes according to institutional standard practice (Section 7.4.4).

[cc] **Skin biopsy.** Pre and on-treatment skin samples will be taken from mirror areas of normal skin. They will be obtained using a 2 and a 4 mm diameter punch biopsy device to create the injury and to collect the wound healing material, respectively. Two skin biopsies will be collected during screening with the first punch (2 mm) followed by the second punch (4 mm) 3 days later and before the first treatment administration. Two on-treatment skin biopsies will also be collected in Cycle 1, with the first punch (2 mm) on Day 5 and the second punch (4 mm) on Day 8.

[dd] **AEs.** All SAEs occurring after informed consent date and until the first study drug administration will be collected following SAE reporting guidelines and will be recorded in the AE eCRF (Section 8.1.1).

[ee] **AEs.** All SAEs/AESIs occurring after 28 days after the last study drug administration or patient EOS should be reported to the Sponsor if the Investigator suspects a causal relationship to the study treatment (Section 8.1.1).

CONFIDENTIAL
May not be used, divulged, published, or otherwise disclosed without the consent of
Molecular Partners AG, Schlieren, Switzerland

ADA, anti-drug antibody; AE, adverse event; AESI, adverse event of special interest; CRS, cytokine release syndrome; CT, computed tomography; D, Day; ECG, electrocardiogram; ECOG, Eastern Cooperative Oncology Group; eCRF, electronic case report form; EOS, end of study; FU, follow-up; HBsAg, hepatitis B surface antigen; HBV, hepatitis B virus; HCV, hepatitis C virus; Ig, immunoglobulin; IRR, infusion related reaction; MRI, magnetic resonance imaging; MUGA, multi-gated acquisition scan; PCR, polymerase chain reaction; PD, progressive disease; PK, pharmacokinetic(s); PS, performance status; RECIST, Response Evaluation Criteria in Solid Tumors; RNA, ribonucleic acid; SAE, serious adverse event; TSH, thyroid-stimulating hormone.

CONFIDENTIAL
May not be used, divulged, published, or otherwise disclosed without the consent of
Molecular Partners AG, Schlieren, Switzerland

Table 8. Schedule of Assessments: Cycle 1 Detailed (Q3W)

	Assessment Time Point	Physical Examination	Vital Signs and Pulse Oximetry	ECG [a]	Local Laboratory Safety Assessments, Pregnancy Test [b], Urinalysis [b]	PK (Serum)	Immunogenicity (ADA, Serum)	Blood Biomarkers (Serum, Plasma and/or Fresh Blood)	Skin Biopsy
Cycle 1	Day 1, before start of infusion	X	X (max -2 h)	X (max -2 h)	X (max -48 h)	X (max -2 h)	X (max -2 h)	X (max -2 h)	
	Day 1, start of infusion		X (± 15 min)						
	Day 1, during infusion every 30 min		X (± 15 min)						
	Day 1, EOI		X (± 15 min)						
	Day 1, EOI + 15 min					X (± 10 min)		X (± 10 min)	
	Day 1, EOI + 6 h		X (± 15 min)			X (± 15 min)		X (± 15 min)	
	Day 2, EOI + 24 h		X (± 4 h)			X (± 4 h)		X (± 4 h)	
	Day 5, EOI + 96 h		X (± 4 h)			X (± 4 h)		X (± 4 h)	X [c]
	Day 8, EOI + 168 h		X (± 4 h)	X (± 4 h)	X (± 4 h)	X (± 4 h)	X (± 4 h)	X (± 4 h)	X [c]
	Day 11, EOI + 240 h		X (± 4 h)			X (± 4 h)		X (± 4 h)	
	Day 15, EOI + 336 h		X (± 4 h)	X (± 4 h)	X (± 4 h)	X (± 4 h)	X (± 4 h)	X (± 4 h)	
	Day 19, EOI + 432 h		X (± 4 h)			X (± 4 h)		X (± 4 h)	

Table 9. Schedule of Assessments: Cycle 2 Detailed (Q3W)

	Assessment Time Point	Physical Examination	Vital Signs and Pulse Oximetry	ECG [a]	Local Laboratory Safety Assessments, Pregnancy Test [b], Urinalysis [b]	PK (Serum)	Immunogenicity (ADA, Serum)	Blood Biomarkers (Serum, Plasma and/or Fresh Blood)	Tumor Assessment	Tumor Biopsy
Cycle 2	Day 1, before start of infusion	X	X (max -2 h)	X (max -2 h)	X (max -48h)	X (max -2 h)	X (max -2 h)	X (max -2 h)		
	Day 1, start of infusion		X (± 15 min)							
	Day 1, during infusion every 30 min		X (± 15 min)							
	Day 1, EOI		X (± 15 min)							
	Day 1, EOI + 15 min					X (± 10 min)				
	Day 1, EOI + 4 h		X (± 15 min)			X (± 15 min)		X (± 15 min)		
	Day 8, EOI + 168 h	X	X (± 4 h)	X (± 4 h)	X (± 4 h)	X (± 4 h)	X (± 4 h)	X (± 4 h)		X (± 3 days) [d]
	Day 15, EOI + 336 h		X (± 4 h)	X (± 4 h)	X (± 4 h)	X (± 4 h)	X (± 4 h)	X (± 4 h)		
	Day of tumor assessment (within 7 days before scheduled infusion of Cycle 3)								X	X [e]

Table 10. Schedule of Assessments: Cycle 3 Detailed (Q3W)

	Assessment Time Point	Physical Examination	Vital Signs and Pulse Oximetry	ECG [a]	Local Laboratory Safety Assessments, Pregnancy Test [b], Urinalysis [b]	Thyroid Function	PK (Serum)	Immunogenicity (ADA, Serum)	Blood Biomarkers (Serum, Plasma and/or Fresh Blood)
Cycle 3	Day 1, before start of infusion	X	X (max -2 h)	X (max -2 h)	X (max -48 h)	X (max -48 h)	X (max -2 h)	X (max -2 h)	X (max -2 h)
	Day 1, start of infusion		X (± 15 min)						
	Day 1, during infusion every 30 min		X (± 15 min)						
	Day 1, EOI		X (± 15 min)						
	Day 1, EOI + 15 min						X (± 10 min)		
	Day 1, EOI + 4 h		X (± 15 min)				X (± 15 min)		X (± 15 min)
	Day 8, EOI + 168 h		X (± 4 h)	X (± 4 h)	X (± 4 h)		X (± 4 h)		X (± 4 h)
	Day 15, EOI + 336 h		X (± 4 h)	X (± 4 h)	X (± 4 h)		X (± 4 h)		X (± 4 h)

Table 11. Schedule of Assessments: Cycle 4 Detailed (Q3W)

	Assessment Time Point	Physical Examination	Vital Signs and Pulse Oximetry	ECG [a]	Local Laboratory Safety Assessments, Pregnancy Test [b], Urinalysis [b]	PK (Serum)	Immunogenicity (ADA, Serum)	Blood Biomarkers (Serum, Plasma and/or Fresh Blood)	Tumor Assessment	Tumor Biopsy
Cycle 4	Day 1, before start of infusion	X	X (max -2 h)	X (max -2 h)	X (max -48 h)	X (max -2 h)	X (max -2 h)	X (max -2 h)		
	Day 1, start of infusion		X (± 15 min)							
	Day 1, during infusion every 30 min		X (± 15 min)							
	Day 1, EOI		X (± 15 min)							
	Day 1, EOI + 15 min					X (± 10 min)				
	Day 1, EOI + 4 h		X (± 15 min)			X (± 15 min)				
	Day 8, EOI + 168 h		X (± 4 h)	X (± 4 h)	X (± 4 h)	X (± 4 h)		X (± 4 h)		
	Day 15, EOI + 336 h		X (± 4 h)	X (± 4 h)	X (± 4 h)	X (± 4 h)				
	Day of tumor assessment (within 7 days before scheduled infusion of Cycle 5)								X	X [e]

Table 12. Schedule of Assessments: Cycle 5 Detailed (Q3W)

	Assessment Time Point	Physical Examination	Vital Signs and Pulse Oximetry	ECG [a]	Local Laboratory Safety Assessments, Pregnancy Test [b], Urinalysis [b]	Thyroid Function	PK (Serum)	Immunogenicity (ADA, Serum)	Blood Biomarkers (Serum, Plasma and/or Fresh Blood)
Cycle 5	Day 1, before start of infusion	X	X (max -2 h)	X (max -2 h)	X (max -48 h)	X (max -48 h)	X (max -2 h)	X (max -2 h)	X (max -2 h)
	Day 1, start of infusion		X (± 15 min)						
	Day 1, during infusion every 30 min		X (± 15 min)						
	Day 1, EOI		X (± 15 min)						
	Day 1, EOI + 15 min						X (± 10 min)		
	Day 1, EOI + 4 h		X (± 15 min)				X (± 15 min)		
	Day 8, EOI + 168 h		X (± 4 h)		X (± 4 h)		X (± 4 h)		
	Day 15, EOI + 336 h		X (± 4 h)		X (± 4 h)		X (± 4 h)		

Table 13. Schedule of Assessments: Cycle 6 and All Further Cycles Detailed (Q3W)

	Assessment Time Point	Physical Examination	Vital Signs and Pulse Oximetry	ECG [a]	Local Laboratory Safety Assessments, Pregnancy Test [b], Urinalysis [b]	Thyroid Function	PK (Serum)	Immunogenicity (ADA, Serum)	Tumor Assessment	Tumor Biopsy
Cycle 6+	Day 1, before start of infusion	X	X (max -2 h)	X (max -2 h)	X (max -48 h)	Cycle 7 and every other cycle thereafter (max -48 h)	X (max -2 h)	X (max -2 h)		
	Day 1, start of infusion		X (± 15 min)							
	Day 1, during infusion every 30 min		X (± 15 min)							
	Day 1, EOI		X (± 15 min)							
	Day 1, EOI + 15 min						X (± 10 min)			
	Day of tumor assessment (within 7 days before scheduled infusion of Cycle 8 and of every third cycle thereafter)								X	X [e]

[a] **ECG.** Dose-escalation part: D1 pre-dose, D8 and D15 Cycle 1 to Cycle 4. From Cycle 5 onwards at D1 pre-dose only. Safety expansion part: All cycles at D1 pre-dose (Section 7.4.6).

[b] **Pregnancy test and urinalysis.** D1 before start of infusion only.

[c] **Skin biopsy.** Pre and on-treatment skin samples will be taken from mirror areas of normal skin. They will be obtained using a 2 and a 4 mm diameter punch biopsy device to create the injury and to collect the wound healing material, respectively. Two skin biopsies will be collected during screening with the first punch (2 mm) followed by the second punch (4 mm) 3 days later and before the first treatment administration. Two on-treatment skin biopsies will also be collected in Cycle 1, with the first punch (2 mm) on Day 5 and the second punch (4 mm) on Day 8.

[d] **Tumor biopsy (fresh), pre and on-treatment.** The biopsies should be performed as follows: a) At least 1 tumor lesion ≥ 20 mm amenable to percutaneous biopsy other than the target lesion(s) used to

follow response as defined by RECIST v1.1. b) For cutaneous or subcutaneous lesions, tumors should be ≥ 5 mm in diameter amenable to biopsy by excisional or punch biopsies without unacceptable risk of a major procedural complication. c) For core needle biopsy specimens, at least 3 to 6 cores with an 18-gauge needle should be collected. d) The on-treatment tumor biopsy should be taken from the same lesion as the pre-treatment biopsy. The biopsied lesion should be large enough to take both biopsies ≥ 1 cm apart.

[e] **Tumor biopsy (fresh), on PD/response (optional).** Patients may agree to an additional tumor biopsy, preferably taken at the time of radiographic PD or response, that will be collected at the discretion of the Investigator and only if the patient is capable of providing it at the time requested.

ADA, anti-drug antibody; ECG, electrocardiogram; EOI, end of infusion (including flushing the entire infusion line with 25 mL of 0.9% NaCl); PD, progressive disease; PK, pharmacokinetic(s).

CONFIDENTIAL
May not be used, divulged, published, or otherwise disclosed without the consent of
Molecular Partners AG, Schlieren, Switzerland

7.1.2 Q1W Dosing Schedule

Table 14. Schedule of Assessments (Q1W)

	Screening	Treatment Period							Follow-up	
**Visit**		**Cycle 1**	**Cycle 2**	**Cycle 3**	**Cycle 4**	**Cycle 5**	**Cycle 6**	**Cycle 7 +**	**Safety FU** [c]	**Survival FU** [d]
Visit days [a]	Days -28 to -1	Day **1**,2,5,**8**,11, **15**,19	Day 1,8,15	Day 1,8,15	Day 1,8,15	Day 1,8,15	Day 1,8,15	Day 1,8,15		
**Assessment**										
Informed consent [e]	X									
Eligibility review [f]	X									
Medical history [g]	X									
Demography	X									
Cancer disease and treatment history [h]	X									
Echocardiography or MUGA scan	X		X (D1) [i]						X	
Physical examination [b, j]	X	X (D1,D8,D15)	X	X	X	X	X	X	X	
ECOG PS [j]	X	X (D1)	X (D1)	X (D1)	X (D1)	X (D1)	X (D1)	X (D1)	X	
Vital signs [b, k, bb]	X	X	X	X	X	X	X	X	X	
ECG [b, l]	X	X (D1,D8,D15) [l]	X (D1) [l]	X (D1) [l]	X (D1) [l]	X (D1) [l]	X (D1) [l]	X (D1) [l]	X	
Thyroid function [b, m]	X		X (D1)		X (D1)		X (Cycle 6 and every other cycle thereafter)		X	
Pulse oximetry [b, n]	X	X	X	X	X	X	X	X	X	
Urinalysis [b, o]	X	X (D1)	X (D1)	X (D1)	X (D1)	X (D1)	X (D1)	X (D1)	X	
Pregnancy test [b, p]	X	X (D1,D8,D15)	X	X	X	X	X	X	X	
Local laboratory safety assessments [b, q]	X	X (D1,D8,D15)	X (D1,D15)	X (D8)	X (D1,D15)	X (D8)	X (D1,D15)	X [q]	X	
Immunology [aa]	X	X (D1)	X (D1)	X (D1)	X (D1)	X (D1)	X (D1)	X (D1)	X	
Central laboratory assessments in the events of IRR or CRS [v]		(X)	(X)	(X)	(X)	(X)	(X)	(X)		
HBV, HCV test [r]	X									
PK [b, s]		X	X	X	X	X	X	X	X	
Immunogenicity (ADA) [b, s]		X (D1,D8,D15)	X	X (D1)	X (D1)	X (D1)	X (D1)	X (D1)	X	
Blood biomarkers [b, s]		X	X	X	X (D1,D8)	X (D1)			X	
Tumor assessment by CT/MRI [u]	X [t]		X [u]		X [u]			X (Cycle 7 and every 3rd cycle	X [u]	

	Screening	Treatment Period							Follow-up	
**Visit**		**Cycle 1**	**Cycle 2**	**Cycle 3**	**Cycle 4**	**Cycle 5**	**Cycle 6**	**Cycle 7 +**	**Safety FU** [c]	**Survival FU** [d]
Visit days [a]	Days -28 to -1	Day **1**,2,5,**8**,11, **15**,19	Day 1,8,15	Day 1,8,15	Day 1,8,15	Day 1,8,15	Day 1,8,15	Day 1,8,15		
								thereafter)		
Tumor biopsy (archival) [w]	X									
Tumor biopsy (fresh), pre and on-treatment [b, x]	X		X (D8)							
Tumor biopsy (fresh), on PD/response (optional) [y]		(X)							(X)	
Skin biopsy [b]	X [cc]	X (D5,D8) [cc]								
Adverse events	X [dd]	X	X	X	X	X	X	X	X	X [ee]
Prior and concomitant medication	X	X	X	X	X	X	X	X	X	
MP0317 administration [z]		X (D1,D8,D15)	X	X	X	X	X	X		
Survival information										X

[a] **Visit days.** Visits are in calendar days, not business days.

[b] **Detailed schedule of assessments.** For assessment time points and/or allowed visit windows see Table 15 to Table 20.

[c] **Safety FU.** At Week 4 (± 1 week) after the last study drug administration (Section 7.3.2). All patients should complete the safety FU period after treatment discontinuation. If the safety FU visit cannot be performed (e.g. withdrawal of consent), the last visit will be considered the safety FU visit for this patient. If a subsequent anticancer treatment is initiated during the 28-day safety FU period, the safety FU visit should be before initiation of such therapy. If this is not possible, the safety (FU) visit will be done as soon as possible after initiation of such therapy.

[d] **Survival FU.** Survival FU will be performed after completion of the last safety FU visit or discontinuation from the study for any reason (i.e. patient EOS). Survival status will be collected by the study site via telephone contact, clinic visits and/or consultation of the patient medical records at approximately 3 (± 2 weeks) and 6 months (± 2 weeks) after patient EOS, or until withdrawal of consent, patient is lost to follow-up, death or study termination by the Sponsor, whichever occurs first (Section 7.3.3).

[e] **Informed consent.** Must be dated and signed before any study-specific procedure is performed (Section 11.1.2).

[f] **Eligibility review.** All inclusion/exclusion criteria must be reviewed during screening (Sections 4.2 and 4.3).

[g] **Medical history.** Including clinically significant diseases, surgeries, reproductive status and medication (including over-the-counter or prescription medicines, vitamins and/or herbal supplements) taken within 28 days before the first study drug administration will be recorded (Sections 6 and 7.4.1).

[h] **Cancer disease and treatment history.** Includes date(s) of diagnosis, classification by origin, tissue type, grade, stage, markers and mutations if available, prior anticancer treatments and procedures (Section 7.4.2).

[i] **Echocardiography or MUGA scan.** Measurements should follow European Society for Cardiology Guidelines 2016[43] and be performed pre-dose (Section 7.4.7).

[j] **Physical examination and ECOG PS.** Will be collected as per institutional standard practice and assessed according to ECOG criteria (Appendix 12.1). Physical examination needs to be done before each study drug administration. Abnormalities observed at screening are recorded in the medical history eCRF. At subsequent visits (or as clinically indicated), limited, symptom-directed physical examination should be performed. New or worsened clinically significant abnormalities occurring after screening should be recorded as AEs in the eCRF (Sections 7.4.3 and 8.1).

[k] **Vital signs.** Assessments include body weight, height (at screening only), body temperature, respiratory rate, pulse rate and blood pressure while the patient is in a supine or seated position and before any blood draws. Assessment of blood pressure and pulse rate will be preceded by at least 5 min rest for the patient in a quiet setting without distractions (e.g. television, mobile phone) (Section 7.4.4). Measurement of body weight will be made with the patient wearing indoor daytime clothing without shoes according to institutional standard practice.

[l] **ECG.** For each patient, ECGs should be obtained from the same machine whenever possible. Dose-escalation part: Screening, Cycle 1 D1, D8 and D15 pre-dose. From Cycle 2 onwards at D1 pre-dose only. Safety expansion part: Screening and all cycles at D1 pre-dose (Section 7.4.6).

[m] **Thyroid function.** TSH, free T3, free T4 will be monitored repeatedly throughout the study to detect any potential autoimmune thyroiditis (Section 7.4.8).

[n] **Pulse oximetry.** To detect presence of hypoxemia (e.g. from possible pneumonitis; Section 7.4.5).

[o] **Urinalysis.** Urine dipstick at screening, on Day 1 of each cycle before study drug administration, and at safety FU visit. If positive findings are noted and are thought to be of clinical significance, detailed urinalysis will be performed at a local laboratory (Section 7.4.9).

[p] **Pregnancy test.** Female patients of childbearing potential will have a serum pregnancy test at screening and a serum or urine pregnancy test before each study drug administration and at the safety FU visit (Sections 7.4.10 and 8.3).

[q] **Local laboratory safety assessments.** Parameters to be evaluated are detailed in Section 7.4.11. Values based on blood samples taken up to 48 h before scheduled laboratory safety assessment will be used by the Investigator to decide if the patient is suitable for study treatment (Section 5.2.3). Laboratory safety assessments from cycle 2 onwards take place before every second drug administration.

[r] **HBV, HCV test.** HBV test includes HBsAg. HCV test includes HCV antibody (if positive an HCV RNA test by PCR must be done; Section 7.4.11).

[s] **PK, immunogenicity (ADA) and blood biomarkers.** PK and immunogenicity (ADA) will be assessed using serum, and blood biomarkers (to evaluate disease status, safety or predictive biomarkers) will be assessed using serum, plasma and/or fresh blood. See Sections 7.4.13, 7.4.14 and 7.4.16.3 and refer to the Laboratory Manual.

[t] **Tumor assessments at screening.** If a tumor assessment (including CT scan according to RECIST v1.1) was performed within 28 days before screening it is not required to be repeated and can be used as screening assessment.

[u] **Tumor assessments.** Tumor measurements will be made at screening and every 6 to 8 weeks after the start of study treatment (<u>within</u> 7 days before first scheduled infusion of Cycles 3, 5 and 8 then every third cycle and according to institutional standard practice) until PD, withdrawal of consent, death, or study termination, whichever occurs first. CT scans will be performed using institutional standard operating procedures. In specific cases, tumor measurements may be performed by MRI scans instead of (or in addition to) CT scans. However, the same imaging modality as used for screening should be used throughout the study (Section 7.4.15).

[v] **Central laboratory assessments in the events of IRR or CRS.** Cytokines, complement factors, drug-specific IgE and tryptase will be assessed in the events of IRR or CRS (Section 7.4.12). Sample should be taken 30-40 minutes after onset of the reaction.

[w] **Tumor biopsy (archival).** If an archival tumor sample is available with an associated pathology report, performed at any time prior to study entry, this should be provided. However, only tissue from core needle, punch or excisional biopsy samples will be considered for study analyses, while fine needle aspiration, brushing and lavage samples are not acceptable. For all biopsy types, at least 5 to 15 unstained good quality slides should be provided or blocks that have sufficient tissue to generate at least 15 slides. Tissue for which the pathology report specifies that the overall tumor content is low (e.g. 'sparse' or 'scant') will not be considered.

[x] **Tumor biopsy (fresh), pre and on-treatment.** The biopsies should be performed as follows: a) At least 1 tumor lesion ≥ 20 mm amenable to percutaneous biopsy other than the target lesion(s) used to follow response as defined by RECIST v1.1. b) For cutaneous or subcutaneous lesions, tumors should be ≥ 5 mm in diameter amenable to biopsy by excisional or punch biopsies without unacceptable risk of a major procedural complication. c) For core needle biopsy specimens, at least 3 to 6 cores with an 18-gauge needle should be collected. d) The on-treatment tumor biopsy should be taken from the same lesion as the pre-treatment biopsy. The biopsied lesion should be large enough to take both biopsies ≥ 1 cm apart.

[y] **Tumor biopsy (fresh), on PD/response (optional).** Patients may agree to an additional tumor biopsy, preferably taken at the time of radiographic PD or response, that will be collected at the discretion of the Investigator and only if the patient is capable of providing it at the time requested.

[z] **MP0317 administration.** The MP0317 infusion solution must be administered completely and the entire infusion line flushed with 25 mL 0.9% NaCl. The infusion duration should be at least 50 min (excluding flushing) and no longer than 2 h (including flushing). Usage of a 0.2 µm in-line filter is mandatory. It is recommended to start infusions in the morning to allow adequate time for pre-infusion assessments, infusion, post-infusion medical supervision and PK assessments (Section 5.2.2). Body weight measured at Day 1 of each treatment cycle should be used for dose calculation. If an infusion bag needs to be prepared one day prior to the Day 1 visit of the respective cycle, body weight measured at the previous visit should be used (Section 5.1.2).

[aa] **Immunology.** Assess beginning of each cycle (parameters to be evaluated are detailed in Section 7.4.11).

[bb] **Height.** At screening only. Measurements will be made with the patient wearing indoor daytime clothing without shoes according to institutional standard practice (Section 7.4.4).

[cc] **Skin biopsy.** Pre and on-treatment skin samples will be taken from mirror areas of normal skin. They will be obtained using a 2 and a 4 mm diameter punch biopsy device to create the injury and to collect the wound healing material, respectively. Two skin biopsies will be collected during screening with the first punch (2 mm) followed by the second punch (4 mm) 3 days later and before the first treatment administration. Two on-treatment skin biopsies will also be collected in Cycle 1, with the first punch (2 mm) on Day 5 and the second punch (4 mm) on Day 8 before drug administration.

[dd] **AEs.** All SAEs occurring after informed consent date and until the first study drug administration will be collected following SAE reporting guidelines and will be recorded in the AE eCRF (Section 8.1.1).

[ee] **AEs.** All SAEs/AESIs occurring after 28 days after the last study drug administration or patient EOS should be reported to the Sponsor if the Investigator suspects a causal relationship to the study treatment (Section 8.1.1).

CONFIDENTIAL
May not be used, divulged, published, or otherwise disclosed without the consent of
Molecular Partners AG, Schlieren, Switzerland

ADA, anti-drug antibody; AE, adverse event; AESI, adverse event of special interest; CRS, cytokine release syndrome; CT, computed tomography; D, Day; ECG, electrocardiogram; ECOG, Eastern Cooperative Oncology Group; eCRF, electronic case report form; EOS, end of study; FU, follow-up; HBsAg, hepatitis B surface antigen; HBV, hepatitis B virus; HCV, hepatitis C virus; Ig, immunoglobulin; IRR, infusion related reaction; MRI, magnetic resonance imaging; MUGA, multi-gated acquisition scan; PCR, polymerase chain reaction; PD, progressive disease; PK, pharmacokinetic(s); PS, performance status; RECIST, Response Evaluation Criteria in Solid Tumors; RNA, ribonucleic acid; SAE, serious adverse event; TSH, thyroid-stimulating hormone.

Table 15. Schedule of Assessments: Cycle 1 Detailed (Q1W)

	Assessment Time Point	Physical Examination	Vital Signs and Pulse Oximetry	ECG [a]	Local Laboratory Safety Assessments, Pregnancy Test [b]	Urinalysis [b]	PK (Serum)	Immunogenicity (ADA, Serum)	Blood Biomarkers (Serum, Plasma and/or Fresh Blood)	Skin Biopsy
Cycle 1	Day 1, before start of infusion	X	X (max -2 h)	X (max -2 h)	X (max -48 h)	X	X (max -2 h)	X (max -2 h)	X (max -2 h)	
	Day 1, start of infusion		X (± 15 min)							
	Day 1, during infusion every 30 min		X (± 15 min)							
	Day 1, EOI		X (± 15 min)							
	Day 1, EOI + 15 min						X (± 10 min)		X (± 10 min)	
	Day 1, EOI + 6 h		X (± 15 min)				X (± 15 min)		X (± 15 min)	
	Day 2, EOI + 24 h		X (± 4 h)				X (± 4 h)		X (± 4 h)	
	Day 5, EOI + 96 h		X (± 4 h)				X (± 4 h)		X (± 4 h)	X [c]
	Day 8, before start of infusion	X	X (max -2 h)	X (max -2 h)	X (max -48 h)		X (max -2 h)	X (max -2 h)	X (max -2 h)	X [c]
	Day 8, start of infusion		X (± 15 min)							
	Day 8, during infusion every 30 min		X (± 15 min)							
	Day 8, EOI		X (± 15 min)							
	Day 8, EOI + 15 min						X (± 10 min)			
	Day 8, EOI + 6 h		X (± 15 min)				X (± 15 min)			
	Day 11, EOI + 72 h		X (± 4 h)				X (± 4 h)		X (± 4 h)	
	Day 15, before start of infusion	X	X (max -2 h)	X (max -2 h)	X (max -48 h)		X (max -2 h)	X (max -2 h)	X (max -2 h)	
	Day 15, start of infusion		X (± 15 min)							
	Day 15, during infusion every 30 min		X (± 15 min)							
	Day 15, EOI		X (± 15 min)							
	Day 15, EOI + 15 min						X (± 10 min)			
	Day 15, EOI + 4 h		X (± 15 min)				X (± 15 min)			
	Day 19, EOI + 96 h		X (± 4 h)				X (± 4 h)		X (± 4 h)	

Table 16. Schedule of Assessments: Cycle 2 Detailed (Q1W)

	Assessment Time Point	Physical Examination	Vital Signs and Pulse Oximetry	ECG[a]	Local Laboratory Safety Assessments	Pregnancy Test[b]	Urinalysis[b], Thyroid Function	PK (Serum)	Immunogenicity (ADA, Serum)	Blood Biomarkers (Serum, Plasma and/or Fresh Blood)	Tumor Assessment	Tumor Biopsy
Cycle 2	Day 1, before start of infusion	X	X (max -2 h)	X (max -2 h)	X (max -48h)	X	X (max -48h)	X (max -2 h)	X (max -2 h)	X (max -2 h)		
	Day 1, start of infusion		X (± 15 min)									
	Day 1, during infusion every 30 min		X (± 15 min)									
	Day 1, EOI		X (± 15 min)									
	Day 1, EOI + 15 min							X (± 10 min)				
	Day 1, EOI + 4 h		X (± 15 min)					X (± 15 min)		X (± 15 min)		
	Day 8, before start of infusion	X	X (max -2 h)			X		X (max -2 h)	X (max -2 h)	X (max -2 h)		X (± 3 days)[d]
	Day 8, start of infusion		X (± 15 min)									
	Day 8, during infusion every 30 min		X (± 15 min)									
	Day 8, EOI		X (± 15 min)									
	Day 8, EOI + 15 min							X (± 10 min)				
	Day 8, EOI + 4 h		X (± 15 min)					X (± 15 min)				
	Day 15, before start of infusion	X	X (max -2 h)		X (max -48h)	X		X (max -2 h)	X (max -2 h)	X (max -2 h)		
	Day 15, start of infusion		X (± 15 min)									
	Day 15, during infusion every 30 min		X (± 15 min)									
	Day 15, EOI		X (± 15 min)									
	Day 15, EOI + 15 min							X (± 10 min)				
	Day of tumor assessment (within 7 days before scheduled infusion of Cycle 3)										X	X[e]

Table 17. Schedule of Assessments: Cycle 3 Detailed (Q1W)

	Assessment Time Point	Physical Examination	Vital Signs and Pulse Oximetry	ECG[a]	Local Laboratory Safety Assessments	Pregnancy Test[b]	Urinalysis[b]	PK (Serum)	Immunogenicity (ADA, Serum)	Blood Biomarkers (Serum, Plasma and/or Fresh Blood)
Cycle 3	Day 1, before start of infusion	X	X (max -2 h)	X (max -2 h)		X	X	X (max -2 h)	X (max -2 h)	X (max -2 h)
	Day 1, start of infusion		X (± 15 min)							
	Day 1, during infusion every 30 min		X (± 15 min)							
	Day 1, EOI		X (± 15 min)							
	Day 1, EOI + 15 min							X (± 10 min)		
	Day 8, before start of infusion	X	X (max -2 h)		X (max -48 h)	X		X (max -2 h)		X (max -2 h)
	Day 8, start of infusion		X (± 15 min)							
	Day 8, during infusion every 30 min		X (± 15 min)							
	Day 8, EOI		X (± 15 min)							
	Day 8, EOI + 15 min							X (± 10 min)		
	Day 15, before start of infusion	X	X (max -2 h)			X		X (max -2 h)		X (max -2 h)
	Day 15, start of infusion		X (± 15 min)							
	Day 15, during infusion every 30 min		X (± 15 min)							
	Day 15, EOI		X (± 15 min)							
	Day 15, EOI + 15 min							X (± 10 min)		

CONFIDENTIAL
May not be used, divulged, published, or otherwise disclosed without the consent of
Molecular Partners AG, Schlieren, Switzerland

Table 18. Schedule of Assessments: Cycle 4 Detailed (Q1W)

	Assessment Time Point	Physical Examination	Vital Signs and Pulse Oximetry	ECG[a]	Local Laboratory Safety Assessments	Pregnancy Test[b]	Urinalysis[b], Thyroid Function	PK (Serum)	Immunogenicity (ADA, Serum)	Blood Biomarkers (Serum, Plasma and/or Fresh Blood)	Tumor Assessment	Tumor Biopsy
Cycle 4	Day 1, before start of infusion	X	X (max -2 h)	X (max -2 h)	X (max -48 h)	X	X (max -48 h)	X (max -2 h)	X (max -2 h)	X (max -2 h)		
	Day 1, start of infusion		X (± 15 min)									
	Day 1, during infusion every 30 min		X (± 15 min)									
	Day 1, EOI		X (± 15 min)									
	Day 1, EOI + 15 min							X (± 10 min)				
	Day 8, before start of infusion	X	X (max -2 h)			X		X (max -2 h)		X (max -2 h)		
	Day 8, start of infusion		X (± 15 min)									
	Day 8, during infusion every 30 min		X (± 15 min)									
	Day 8, EOI		X (± 15 min)									
	Day 8, EOI + 15 min							X (± 10 min)				
	Day 15, before start of infusion	X	X (max -2 h)		X (max -48 h)	X		X (max -2 h)				
	Day 15, start of infusion		X (± 15 min)									
	Day 15, during infusion every 30 min		X (± 15 min)									
	Day 15, EOI		X (± 15 min)									
	Day 15, EOI + 15 min							X (± 10 min)				
	Day of tumor assessment (within 7 days before scheduled infusion of Cycle 5)										X	X[e]

Table 19. Schedule of Assessments: Cycle 5 Detailed (Q1W)

	Assessment Time Point	Physical Examination	Vital Signs and Pulse Oximetry	ECG[a]	Local Laboratory Safety Assessments	Pregnancy Test[b]	Urinalysis[b]	PK (Serum)	Immunogenicity (ADA, Serum)	Blood Biomarkers (Serum, Plasma and/or Fresh Blood)
Cycle 5	Day 1, before start of infusion	X	X (max -2 h)	X (max -2 h)		X	X	X (max -2 h)	X (max -2 h)	X (max -2 h)
	Day 1, start of infusion		X (± 15 min)							
	Day 1, during infusion every 30 min		X (± 15 min)							
	Day 1, EOI		X (± 15 min)							
	Day 1, EOI + 15 min							X (± 10 min)		
	Day 8, before start of infusion	X	X (max -2 h)		X (max -48 h)	X		X (max -2 h)		
	Day 8, start of infusion		X (± 15 min)							
	Day 8, during infusion every 30 min		X (± 15 min)							
	Day 8, EOI		X (± 15 min)							
	Day 8, EOI + 15 min							X (± 10 min)		
	Day 15, before start of infusion	X	X (max -2 h)			X		X (max -2 h)		
	Day 15, start of infusion		X (± 15 min)							
	Day 15, during infusion every 30 min		X (± 15 min)							
	Day 15, EOI		X (± 15 min)							
	Day 15, EOI + 15 min							X (± 10 min)		

CONFIDENTIAL
May not be used, divulged, published, or otherwise disclosed without the consent of
Molecular Partners AG, Schlieren, Switzerland

Table 20. Schedule of Assessments: Cycle 6 and All Further Cycles Detailed (Q1W)

	Assessment Time Point	Physical Examination	Vital Signs and Pulse Oximetry	ECG [a]	Local Laboratory Safety Assessments	Pregnancy Test [b]	Urinalysis [b]	Thyroid Function	PK (Serum)	Immunogenicity (ADA, Serum)	Tumor Assessment	Tumor Biopsy
Cycle 6+	Day 1, before start of infusion	X	X (max -2 h)	X (max -2 h)	X (max -48 h)	X	X	Cycle 6 and every other cycle thereafter (max -48 h)	X (max -2 h)	X (max -2 h)		
	Day 1, start of infusion		X (± 15 min)									
	Day 1, during infusion every 30 min		X (± 15 min)									
	Day 1, EOI		X (± 15 min)									
	Day 1, EOI + 15 min								X (± 10 min)			
	Day 8, before start of infusion	X	X (max -2 h)			X			X (max -2 h)			
	Day 8, start of infusion		X (± 15 min)									
	Day 8, during infusion every 30 min		X (± 15 min)									
	Day 8, EOI		X (± 15 min)									
	Day 8, EOI + 15 min								X (± 10 min)			
	Day 15, before start of infusion	X	X (max -2 h)		X (max -48 h)	X			X (max -2 h)			
	Day 15, start of infusion		X (± 15 min)									
	Day 15, during infusion every 30 min		X (± 15 min)									
	Day 15, EOI		X (± 15 min)									
	Day 15, EOI + 15 min								X (± 10 min)			
	Day of tumor assessment (within 7 days before scheduled infusion of Cycle 8 and of every third cycle thereafter)										X	X [e]

a **ECG.** Dose-escalation part: Cycle 1 D1, D8 and D15 pre-dose. From Cycle 2 onwards at D1 pre-dose only. Safety expansion part: All cycles at D1 pre-dose (Section 7.4.6).

b **Pregnancy test and urinalysis.** On the indicated days, before start of infusion.

c **Skin biopsy.** Pre and on-treatment skin samples will be taken from mirror areas of normal skin. They will be obtained using a 2 and a 4 mm diameter punch biopsy device to create the injury and to collect the wound healing material, respectively. Two skin biopsies will be collected during screening with the first punch (2 mm) followed by the second punch (4 mm) 3 days later and before the first treatment administration. Two on-treatment skin biopsies will also be collected in Cycle 1, with the first punch (2 mm) on Day 5 and the second punch (4 mm) on Day 8 before drug administration.

d **Tumor biopsy (fresh), pre and on-treatment.** The biopsies should be performed as follows: a) At least 1 tumor lesion ≥ 20 mm amenable to percutaneous biopsy other than the target lesion(s) used to

follow response as defined by RECIST v1.1. b) For cutaneous or subcutaneous lesions, tumors should be ≥ 5 mm in diameter amenable to biopsy by excisional or punch biopsies without unacceptable risk of a major procedural complication. c) For core needle biopsy specimens, at least 3 to 6 cores with an 18-gauge needle should be collected. d) The on-treatment tumor biopsy should be taken from the same lesion as the pre-treatment biopsy. The biopsied lesion should be large enough to take both biopsies ≥ 1 cm apart.

[e] **Tumor biopsy (fresh), on PD/response (optional).** Patients may agree to an additional tumor biopsy, preferably taken at the time of radiographic PD or response, that will be collected at the discretion of the Investigator and only if the patient is capable of providing it at the time requested.

ADA, anti-drug antibody; ECG, electrocardiogram; EOI, end of infusion (including flushing the entire infusion line with 25 mL of 0.9% NaCl); PD, progressive disease; PK, pharmacokinetic(s).

CONFIDENTIAL
May not be used, divulged, published, or otherwise disclosed without the consent of
Molecular Partners AG, Schlieren, Switzerland

7.2 Screening Period

Patients will be screened for eligibility to participate in the study. All patients must provide written informed consent before any study-specific procedures being performed (Section 11.1.2).

The screening period starts at the informed consent date, lasts for a maximum of 28 days and ends on the day of the first study drug administration.

In the event that a laboratory parameter listed in the eligibility criteria is outside the protocol-specified range at screening, the assessment may be repeated once and as soon as possible. If the repeat value remains outside the protocol-specified range, the patient must not be included in the study.

Data collected during routine work-up before the informed consent date may be used for assessments as appropriate. If a tumor assessment (including CT scan according to RECIST v1.1) was performed within 28 days before screening it is not required to be repeated and can be used as screening assessment.

7.2.1 Waivers to Study Protocol

No waivers to the study protocol will be granted by the Investigator and the Sponsor or its designee for any patient enrolled into the study. Any questions regarding a patient's eligibility should be discussed with the Sponsor.

7.2.2 Patient Enrollment and Eligibility Recommendation

A centralized patient enrollment process will be used to track patient screening and planned timing of first study drug administration. Once an Investigator has verified that the patient has signed the informed consent form (ICF) (Section 11.1.2) and meets all eligibility criteria, they will complete the Patient Eligibility Form (provided separately) and send it to the Sponsor's Medical Monitor/Director as specified on the form.

The Sponsor's Medical Monitor/Director may request additional information from the Investigator. After evaluation of eligibility the Sponsor's Medical Monitor/Director or designee will return the Patient Eligibility Form to the Investigator within one working day, with or without a recommendation for study treatment. For patients recommended for treatment, the dosing schedule and cohort to which the patient is allocated will be specified on the form. For patients not recommended for study treatment, the reason will be specified.

The results of the patient eligibility assessment must be entered in the patient medical records.

7.2.3 Treatment Assignment

This is an open-label study. Patients who have met all eligibility criteria and are recommended for treatment will be assigned to a currently open dosing schedule and cohort.

7.2.4 Screen Failures

Screen failures are defined as patients who consented to participate in the clinical study, but who did not receive study treatment for any reason. A minimal set of screen failure information is required to ensure transparent reporting of screen failure participants, to meet the Consolidated Standards of Reporting Trials (CONSORT) publishing requirements and to respond to queries from regulatory authorities. This includes demography, reason for screen failure, eligibility criteria and any SAEs.

CONFIDENTIAL
May not be used, divulged, published, or otherwise disclosed without the consent of
Molecular Partners AG, Schlieren, Switzerland

In specific cases, in agreement with the Sponsor, screen failures may be rescreened. If patients who fail screening are subsequently rescreened, a limited set of screening assessments may be performed as determined by the Investigator in consultation with the Sponsor.

7.3 Treatment Period and Follow-up

7.3.1 Treatment Period and End of Treatment

On-study evaluations will be performed as per the schedule of assessments (Section 7.1) and as detailed in Section 7.4. Assessments during unscheduled visits should be performed as clinically indicated and recorded in the eCRF.

Laboratory assessments performed as a part of the screening evaluations within 48 hours before the first study drug administration are not required to be repeated on the day of first study drug administration (Section 5.2.3).

Allowed visit/assessment windows are detailed in Table 8 to Table 13 (q3w dosing schedule) and Table 15 to Table 20 (q1w dosing schedule), and will not constitute a protocol deviation.

Patients can only receive study treatment if the requirements specified in Sections 5.2.3 and 5.2.9 are met.

Study treatment will be administered as described in Section 5.2.2 until any of the events described in Section 7.3.4 occurs (EOT). The EOT date is defined as the date when the decision is taken for the patient to discontinue from study treatment for any reason.

Treatment beyond PD will be allowed as per iRECIST[4] if the Investigator believes that the patient is still receiving clinical benefit from study treatment and that the potential benefit of continuing study treatment outweighs any potential risk, and only with Sponsor approval.

7.3.2 Safety Follow-up and End of Study

A safety FU visit will be scheduled at Week 4 (± 1 week) after the last study drug administration. Evaluations will be performed as per the schedule of assessments (Table 7 and Table 14) and as detailed in Section 7.4. All patients should complete the safety FU period after treatment discontinuation. If the safety FU visit cannot be performed (e.g. withdrawal of consent), the last visit will be considered the safety FU visit for this patient.

If a subsequent anticancer treatment is initiated during the 28-day safety FU period, the safety FU visit should be before initiation of such therapy. If this is not possible, the safety FU visit will be done as soon as possible after initiation of such therapy but not later than 28 days after last study drug administration.

Completion of safety FU or discontinuation from study for any reason defines patient EOS.

7.3.3 Survival Follow-up

Survival FU will be performed after completion of the safety FU visit or discontinuation from the study for any reason (i.e. patient EOS). Survival status will be collected by the study site via telephone contact, clinic visits and/or consultation of the patient medical records approximately 3 (± 2 weeks) and 6 months (± 2 weeks) after patient EOS or until withdrawal of consent, patient is lost to follow-up, death or study termination by the Sponsor, whichever occurs first.

CONFIDENTIAL
May not be used, divulged, published, or otherwise disclosed without the consent of
Molecular Partners AG, Schlieren, Switzerland

7.3.4 Patient Discontinuation

The reason(s) for discontinuation and the corresponding dates must be documented in the patient's source documents and in the eCRF.

A patient will be discontinued from study treatment (EOT) and/or the study (EOS) for any of the following reasons:

- PD
- Unacceptable toxicity
- Patient refusal to continue study treatment [a]
- Withdrawal of consent
- Pregnancy (Section 8.3)
- Study terminated by Sponsor
- Lost to follow-up [b]
- Death
- Investigator decision
- Screen failure
- Other

[a] Patients who wish to discontinue from further study treatment should be encouraged to complete the safety and/or survival FU.

[b] Patients will be considered lost to follow-up if they miss 3 consecutive study contacts by clinic visits and/or telephone.

7.3.5 Withdrawal of Consent

Patients may discontinue from the study at their own request at any time, for any reason, without jeopardizing their current or future care.

If a patient withdraws consent from the study, the Investigator should make a reasonable effort to ascertain the reason(s) for withdrawal of consent, while fully respecting the patient's rights, and record it in the patient's source documents.

7.4 Study Assessments

See the schedule of assessments (Section 7.1 and Table 7 through Table 20) for the timing, timing windows and visit frequency of all assessments listed below.

7.4.1 Medical History and Demography

The patient's medical history will be obtained by the Investigator or qualified designee.

Medical history, including clinically significant diseases, surgeries, reproductive status and prior medication (including over-the-counter or prescription medicines, vitamins and/or herbal supplements) taken within 28 days before the first study drug administration, as described in Section 6, will be recorded.

CONFIDENTIAL
May not be used, divulged, published, or otherwise disclosed without the consent of
Molecular Partners AG, Schlieren, Switzerland

Medical history will include all active conditions and any condition diagnosed that are considered to be clinically significant by the Investigator.

Demographic information will be collected.

7.4.2 Cancer Disease and Treatment History

Information on cancer disease, including date(s) of diagnosis, classification by origin, tissue type, grade, stage, markers and mutations if available and prior anticancer treatments and procedures will be collected at screening.

7.4.3 Physical Examination and ECOG PS

Physical examination and performance status will be collected as per institutional standard practice and assessed according to ECOG criteria (Appendix 12.1).

Physical examination needs to be done before each study drug administration and at Cycle 2 Day 8 during dose escalation phase for q3w dosing schedule. Abnormalities observed at screening are recorded in the medical history eCRF. At subsequent visits (or as clinically indicated), limited, symptom-directed physical examination should be performed. New or worsened clinically significant abnormalities occurring after screening should be recorded as AEs in the eCRF (Section 8.1).

Investigators should pay special attention to clinical signs related to previous serious illnesses.

7.4.4 Vital Signs

Assessments include body weight, height (at screening only), body temperature, respiratory rate, pulse rate and blood pressure while the patient is in a supine or seated position and before any blood draws. Assessment of blood pressure and pulse rate will be preceded by at least 5 min rest for the patient in a quiet setting without distractions (e.g. television, mobile phone).

Body weight and height measurements will be made with the patient wearing indoor daytime clothing without shoes, according to institutional standard practice.

7.4.5 Pulse Oximetry

Pulse oximetry is used as a general method for monitoring patient oxygenation in the clinical care setting. By forewarning about the presence of hypoxemia, pulse oximetry may lead to quicker treatment of serious hypoxemia (e.g. from possible pneumonitis) and circumvent serious complications. It will be performed every cycle. At dosing visits, oxygenation at the timepoints indicated in the schedules of assessment will be captured in the eCRF.

7.4.6 Electrocardiogram

Single 12-lead ECG will be obtained using an ECG machine that automatically calculates heart rate and measures PR interval, QRS, QT and QTc intervals. For QTc, the Fridericia-correction should be used. ECGs performed on the day of treatment administration must be performed pre-dose.

For each patient, ECGs should be obtained from the same machine whenever possible.

The ECGs should be printed out and adequately labelled and signed by the site personnel performing the evaluation. If fading of the original ECG printout cannot be excluded, a copy needs to be retained as a source document.

The Investigator's assessment of 'normal ECG' or 'abnormal ECG' as well as its clinical significance will be recorded on the ECG trace printouts as a source document and in the eCRF. Machine-

CONFIDENTIAL
May not be used, divulged, published, or otherwise disclosed without the consent of
Molecular Partners AG, Schlieren, Switzerland

calculated ECG abnormalities must be confirmed manually by the Investigator or a competent designee. In case of discrepant ECG interpretation, manual assessment is considered more reliable.

7.4.7 Echocardiography or MUGA Scan

Left ventricular ejection fraction (LVEF) is assessed as follows:

- By echocardiography or MUGA scan
- Measurements should follow European Society for Cardiology Guidelines 2016[43]
- Printouts and/or reports need to be adequately signed and labelled by the physician performing the evaluation and a copy retained as a source document
- The assessment will be recorded as a source document and in the eCRF

7.4.8 Thyroid Function Test

Thyroid function (thyroid-stimulating hormone [TSH], free T3, free T4) will be monitored repeatedly throughout the study to detect any potential autoimmune thyroiditis emerging from immune stimulating treatments.

7.4.9 Urinalysis

Urinalysis will be performed with urine dipstick at screening, on Day 1 of each cycle before study drug administration, and at safety FU visit. If positive findings are noted and are thought to be of clinical significance, detailed urinalysis will be performed at a local laboratory.

7.4.10 Pregnancy Test

Female patients of childbearing potential will have a serum pregnancy test at screening and a serum or urine pregnancy test before each study drug administration and at the safety FU visit.

For actions to take in case of pregnancy see Section 8.3.

7.4.11 Local Laboratory (Safety) Assessments

All laboratory parameters listed in Table 21 will be done by local laboratories according to standard practice.

Local laboratory safety assessments (including hematology, clinical chemistry, coagulation, liver function, kidney function, cardiac parameters, inflammatory parameters) based on blood samples taken up to 48 h before study drug administration at timepoints indicated in the Schedule of Assessment in Table 7 and Table 14 (including screening, which will be based on protocol eligibility criteria for the initial administration [Section 4]), will be used by the Investigator to decide if the patient is suitable for receiving study treatment (Sections 5.2.3 and 5.2.9).

All local laboratory results will be kept as source documents and must be entered in the eCRF. The Investigator must review all laboratory reports and document this review throughout the study.

All results must be evaluated for criteria defining an AE (Sections 8.1.1 and 8.1.2) and reported as such if the criteria are met.

All laboratory tests with abnormal results considered clinically significant during or after the treatment period should be repeated until they are no longer considered clinically significant. If this does not happen or does not happen within a period of time judged reasonable by the Investigator, the etiology should be identified and the Sponsor notified.

CONFIDENTIAL
May not be used, divulged, published, or otherwise disclosed without the consent of
Molecular Partners AG, Schlieren, Switzerland

Table 21. Local Laboratory Panel and Parameters

**Laboratory Panel**	**Laboratory Parameters**
Hematology [a]	Hemoglobin, hematocrit, RBC count, RBC indices (including MCV, MCH, MCHC), absolute and differential count of WBCs, neutrophils, lymphocytes, eosinophils, basophils, monocytes, platelet count
Clinical chemistry [a]	Sodium, magnesium, calcium, potassium, phosphate, chloride, glucose, albumin, total protein, cholesterol, lipase, alpha-amylase, triglycerides, LDH
Coagulation [a]	INR, PT, aPTT
Liver function [a]	Total bilirubin and if elevated, direct and indirect bilirubin, AST, ALT, ALP
Kidney function [a]	Serum creatinine, BUN or urea, uric acid, creatinine clearance on the basis of Cockcroft-Gault GFR estimation
Cardiac parameters [a]	CK, troponin
Inflammatory parameters [a]	CRP
Immunology	Total IgA, total IgE, total IgG, total IgM
Urinalysis	Section 7.4.9
Pregnancy	Section 7.4.10
HBV, HCV	HBV test includes HBsAg; HCV test includes HCV antibody (if positive an HCV RNA test by PCR must be done)
Thyroid function	TSH, free T3, free T4 (Section 7.4.8)

[a] Laboratory safety assessments.

ALP, alkaline phosphatase; ALT, alanine transaminase; aPTT, activated partial thromboplastin time; AST, aspartate aminotransferase; BUN, blood urea nitrogen; CK, creatine kinase; CRP, C-reactive protein; GFR, glomerular filtration rate; HBsAg, hepatitis B surface antigen; HBV, hepatitis B virus; HCV, hepatitis C virus; Ig, immunoglobulin; INR, international normalized ratio; LDH, lactate dehydrogenase; MCH, mean corpuscular hemoglobin; MCHC, mean corpuscular hemoglobin concentration; MCV, mean corpuscular volume; PCR, polymerase chain reaction; PT, prothrombin time; RBC, red blood cell; RNA, ribonucleic acid; TSH, thyroid-stimulating hormone; WBC, white blood cell.

7.4.12 Central Laboratory Assessments

In the event of IRR or CRS, blood samples will be taken to evaluate levels of cytokines (IL-2, IL-6, IL-8, TNF-α, IFN-γ), complement factors (C3a, C5a, SC5b-9) and other analytes (e.g. drug-specific

CONFIDENTIAL
May not be used, divulged, published, or otherwise disclosed without the consent of
Molecular Partners AG, Schlieren, Switzerland

immunoglobulin (Ig) E and tryptase. The measurements will be performed in a central laboratory. The sample will be taken within 30 – 40 minutes of onset of the reaction.

7.4.13 Pharmacokinetic Assessments

Blood samples will be collected from all patients who are scheduled for the first study drug administration, to measure serum concentrations of MP0317 before treatment start and on study at protocol-defined time points in relation to end of infusion (EOI), as detailed in Section 7.1, Table 8 through Table 13 (q3w dosing schedule) and Table 15 to Table 20 (q1w dosing schedule). EOI is defined as completing the whole IMP infusion including flushing the entire infusion line with 25 mL of 0.9% NaCl (at the same rate as the infusion itself).

Planned PK blood sampling times should be adhered to as closely as possible. It is essential that the actual times (i.e. infusion start and stop) are recorded accurately in the patient medical records and in the eCRF, along with the date and time of collection of each blood sample. Full instructions for PK sample preparation, storage and shipping are provided in a Laboratory Manual.

Serum samples must be sent as soon as possible to a central laboratory for analysis, to allow for availability of results when reviewing safety data.

7.4.14 Immunogenicity Assessments

Blood samples will be collected from all patients who are scheduled for the first study drug administration to assess serum titers of ADA (i.e. drug-specific IgG and IgM) before treatment start and on-study at protocol-defined time points detailed in Section 7.1, Table 8 through Table 13 (q3w dosing schedule) and Table 15 to Table 20 (q1w dosing schedule).

Analyses will be performed centrally. Full instructions for sample preparation, handling procedures, storage and shipping of samples will be provided in the Laboratory Manual.

7.4.15 Efficacy Assessments

Patients will be evaluated for response using RECIST v1.1[5] (Appendix 12.3) and iRECIST[4] (Appendix 12.4) as per Investigator assessment.

Tumor measurements will be made at screening and every 6 to 8 weeks after the start of study treatment (within 7 days before scheduled infusion on Day 1 of Cycles 3, 5 and 8 then every third cycle and according to institutional standard practice) until PD, withdrawal of consent, death, or study termination, whichever occurs first. Tumor assessment results must be available before next planned study drug administration that follows the tumor assessment.

CT scans will be performed using institutional standard operating procedures. In specific cases, tumor measurements may be performed by magnetic resonance imaging (MRI) scans instead of (or in addition to) CT scans. However, the same imaging modality as used for screening should be used throughout the study. Scans will be read by institutional radiologists and reported. The reports will be filed in source documents and the results entered into the eCRF.

7.4.16 Biomarker Assessments

The time and date of collection plus further sampling information will be recorded in the patient medical records and eCRF for all samples (tumor tissue, skin, and blood). Analyses will be performed centrally. Full instructions for tumor, skin and liquid biopsy sample preparation, handling procedures, storage and shipping are provided in the Laboratory Manual, as well as guidelines for procedures to

CONFIDENTIAL
May not be used, divulged, published, or otherwise disclosed without the consent of
Molecular Partners AG, Schlieren, Switzerland

follow for any unused samples obtained from a patient who withdraws consent at any time during or after the study.

Biomarker sampling will be performed at the time points detailed in Section 7.1, Table 8 through Table 13 (q3w dosing schedule) and Table 15 to Table 20 (q1w dosing schedule).

7.4.16.1 Tumor specimens

Tumor biopsy (archival). If an archival tumor sample is available with an associated pathology report, performed at any time prior to study entry, this should be provided. However, only tissue from core needle, punch or excisional biopsy samples will be considered for study analyses, while fine needle aspiration, brushing and lavage samples are not acceptable. For all biopsy types, at least 5 to 15 unstained good quality slides should be provided or blocks that have sufficient tissue to generate at least 15 slides. Tissue for which the pathology report specifies that the overall tumor content is low (e.g. 'sparse' or 'scant') will not be considered.

Tumor biopsy (fresh), pre and on-treatment. A pre-treatment tumor biopsy will be performed during the screening period. An on-treatment tumor biopsy should be performed on Day 8 of Cycle 2. The biopsies should be performed as follows:

- At least 1 tumor lesion ≥ 20 mm amenable to percutaneous biopsy other than the target lesion(s) used to follow response as defined by RECIST v1.1.
- For cutaneous or subcutaneous lesions, tumors should be ≥ 5 mm in diameter amenable to biopsy by excisional or punch biopsies without unacceptable risk of a major procedural complication.
- For core needle biopsy specimens, at least 3 to 6 cores with an 18-gauge needle should be collected. Two thirds of the core biopsies (2 to 4 specimens) will be processed into formalin-fixed paraffin-embedded (FFPE) blocks for multiplex immunofluorescence tissue analysis. One third of the core biopsies (1 to 2 specimens) will be cryo-preserved for targeted gene-expression analysis.
- The on-treatment tumor biopsy should be taken from the same lesion as the pre-treatment biopsy. The biopsied lesion should be large enough to take both biopsies ≥ 1 cm apart.

Tumor biopsy (fresh), on PD/response (optional). Patients may agree to an additional tumor biopsy, preferably taken at the time of radiographic PD or response, that will be collected at the discretion of the Investigator and only if the patient is capable of providing it at the time requested.

7.4.16.2 Skin specimens

Pre and on-treatment skin samples will be taken from the same or mirror areas of normal skin. They will be obtained using a 2 and a 4 mm diameter punch biopsy device to create the wound and to collect the wound healing material, respectively.

Two skin biopsies will be collected during screening with the first punch (2 mm) followed by the second punch (4 mm) 3 days later and before the first treatment administration. Two on-treatment skin biopsies will also be collected in Cycle 1, with the first punch (2 mm) on Day 5 and the second punch (4 mm) on Day 8.

CONFIDENTIAL
May not be used, divulged, published, or otherwise disclosed without the consent of
Molecular Partners AG, Schlieren, Switzerland

7.4.16.3 Blood specimens

Serial blood samples will be collected at time points described in the schedule of assessments (Section 7.1) to evaluate disease status, safety or predictive biomarkers to support the exploratory objective (Section 2).

7.4.16.4 Analyses

See Sections 9.11 and 9.12 for planned and exploratory analyses of tumor markers and pharmacodynamic biomarkers.

CONFIDENTIAL
May not be used, divulged, published, or otherwise disclosed without the consent of
Molecular Partners AG, Schlieren, Switzerland

8 SAFETY PLAN

Measures will be taken to ensure the safety of patients participating in this study, including the use of inclusion and exclusion criteria (Section 4) and close patient monitoring during the study. Safety will be assessed throughout the study via measurement of laboratory parameters (including hematology, clinical chemistry, coagulation, liver, kidney, cardiac and thyroid function tests and inflammatory parameters), vital signs, physical examination, weight, ECOG PS evaluation, ECG evaluation, MUGA, and continuous monitoring of DLTs and AEs, including SAEs and AESIs.

All SAEs and protocol-defined AESIs will be reported in an expedited way (Section 8.2).

Administration of study treatment will be performed in a monitored setting with immediate access to trained personnel and adequate equipment/treatment to manage potentially serious reactions.

Planned time points for all safety assessments are provided in the schedule of assessments (Section 7.1).

To date too few patients have been exposed to MP0317 in the current study, therefore any safety information is based on non-clinical and clinical studies and published data on similar compounds. Thus, the entire safety profile is not known at this point in time.

Potential safety issues anticipated in this study, as well as measures intended to avoid or minimize such toxicities, are outlined in Section 5.2.9.

8.1 Adverse Events

8.1.1 Definition and Reporting

An AE is defined as the appearance of (or worsening of any pre-existing) undesirable sign(s), symptom(s) or medical condition(s) that occurs after patient's signed informed consent has been obtained.

After informed consent date, AEs will be reported as follows:

- All SAEs occurring after informed consent date and until the first study drug administration will be reported via SAE/AESI report form following SAE reporting guidelines and will be recorded in the AE eCRF.
- All AEs (serious, non-serious, protocol-defined AESIs) occurring after the first study drug administration and until 28 days after the last study drug administration or patient EOS will be recorded in the AE sections of eCRF.
- All SAEs/AESIs occurring after the first study drug administration and until 28 days after the last study drug administration or patient EOS will be reported via SAE/AESI report form following SAE reporting guidelines.
- All SAEs/AESIs occurring after 28 days after the last study drug administration or patient EOS should be reported if the Investigator suspects a causal relationship to the study treatment using the eCRF and SAE/AESI report form.
- Unresolved ongoing SAEs/AESIs will be followed up until resolved or deemed unresolvable (i.e. chronic) or return to the patient's screening condition. The Sponsor and the Investigator will agree on any appropriate, additional follow-up for these patients on a case-by-case basis.

CONFIDENTIAL
May not be used, divulged, published, or otherwise disclosed without the consent of
Molecular Partners AG, Schlieren, Switzerland

Assessment should be made at each visit (or more frequently, if necessary) on any changes in severity, the suspected relationship to study treatment, the interventions required to treat the condition and the outcome.

AEs (including abnormal laboratory results that constitute AEs; Section 8.1.2) should be described using the underlying diagnosis whenever possible, rather than individual signs and symptoms. When a clear diagnosis cannot be identified each sign or symptom should be reported as a separate AE.

The severity of AEs will be assessed according to NCI CTCAE v5.0 (http://evs.nci.nih.gov/ftp1/CTCAE/). If an AE term is not specifically listed, severity will be assessed according to the general grading guidelines in NCI CTCAE v5.0. Each change in severity during the course of an AE must be recorded separately.

The occurrence of AEs should be sought by non-directive questioning of the patient during the screening period after the informed consent date and at each visit during the study. AEs also may be detected when they are reported by the patient during the screening process or between visits, or through physical examination, laboratory tests or other assessments. A qualified Investigator will evaluate each AE to determine:

1. Severity according to NCI CTCAE v5.0
2. Duration (start and end date [including start and end time for IRRs] or if ongoing at end of study)
3. Relationship to study treatment (i.e. causality)
4. Action taken with respect to study treatment (not changed, interrupted, withdrawn, unknown, not applicable)
5. Whether treatment (e.g. medication, surgery etc.) was required
6. Whether it is an SAE (Section 8.2.1)
7. Outcome (not recovered/not resolved, recovered/resolved, recovering/resolving, recovered/resolved with sequelae, fatal, unknown)

In terms of assessment of causality, the Investigator must assess and document the relationship between study treatment and occurrence of each AE. It is an essential criterion used when determining regulatory reporting requirements. Each AE must be assessed as either:

- Unrelated: Bears no relation to timing of study treatment and similar symptoms or signs expected in the disease process and does not recur on re-challenge
- Related AE: Existence of facts, evidence and/or arguments to suggest a causal relationship, rather than that a relationship cannot be ruled out
    - AEs considered related to study treatment will be considered adverse reactions
    - Alternative causes, such as underlying disease(s), concomitant treatment and other risk factors, as well as the temporal relationship of the event to study drug administration will be considered and investigated
    - For marketed products the Investigator should consult the IB/Product Information (PI)/Summary of Product Characteristics (SmPC)

Progression of disease under study (including fatal outcome), if evaluated by an appropriate method (e.g. RECIST, iRECIST), **should not** be reported as an AE/SAE. Also, signs and symptoms, clinically significant abnormal laboratory findings or abnormal results of safety assessments which are

associated with the disease under study, unless judged by the Investigator to be more severe than expected for the patient's condition, **should not** be reported as an AE/SAE.

Information about common side effects of study treatment already known can be found in the Reference Safety Information of the IB or will be communicated between IB updates in the form of Investigator Notifications. This information will be included in the patient ICF and should be discussed with the patient during the study as needed.

8.1.2 Abnormal Laboratory Results

Abnormal laboratory results that constitute an AE in their own right (are considered clinically significant, induce clinical signs and symptoms, require concomitant therapy [e.g. hematologic abnormality that requires transfusion or hematologic stem cell support] or require changes in study treatment, as defined by the protocol), should be recorded as AEs in the eCRF. Whenever possible, a diagnosis, rather than a symptom should be provided (e.g. anemia instead of low hemoglobin). Abnormal laboratory results that meet the criteria of AE should be followed until they have returned to normal or screening value. When an abnormal laboratory result corresponds to a sign/symptom or an already reported AE, it is not necessary to record it as an additional event.

Abnormal laboratory results, that do not meet the above definition of an AE, should not be reported as such.

8.1.3 Adverse Events of Special Interest

AESIs are defined as events (serious or non-serious) which are of scientific and medical concern specific to the Sponsor's product or program, for which ongoing monitoring and rapid communication by the Investigator to the Sponsor may be appropriate. Such events may require further investigation in order to characterize and understand them.

AESIs are defined on the basis of an ongoing review of the safety data.

For MP0317, the following AEs will be considered AESIs and will be reported to the Sponsor within 24 hours of receipt of the information, even if not classified as serious:

- Non-infectious pneumonitis any Grade
- Non-infectious colitis any Grade
- Non-infectious nephritis ≥ Grade 2 and as defined in Table 4
- Endocrinopathies ≥ Grade 3
- Non-infectious hepatitis any Grade and as defined in Table 4
- Skin reactions ≥ Grade 3
- Immune-related adverse reactions ≥ Grade 2
- IRR ≥ Grade 2
- CRS any Grade
- Tumor lysis syndrome any Grade

8.2 Serious Adverse Events

8.2.1 Definition of an SAE

An SAE is any AE that meets any of the criteria described in Table 22.

The terms 'severe' and 'serious' are not synonymous. A grade 3 or 4 event (severe) as per NCI CTCAE does not automatically indicate an SAE unless it meets the definition of serious and/or per Investigator judgment. Severity refers to the intensity of an AE according to NCI CTCAE v5.0, the event itself may be of relatively minor medical significance (such as severe headache without any further findings). Severity and seriousness need to be assessed independently from each other for each AE recorded in the eCRF.

Table 22. SAE Definition

**An SAE is defined as any untoward medical occurrence that:**
**Results in death**
**Is life-threatening**<br>The term 'life-threatening' in the definition of 'serious' refers to an event in which the patient was at risk of death at the time of the event. It does not refer to an event, which hypothetically might have caused death, if it were more severe.
**Requires inpatient hospitalization or prolongation of existing hospitalization**<br>In general, hospitalization (if not required by protocol procedures) signifies that the patient has been detained (usually involving at least an overnight stay) at the hospital or emergency ward for observation and/or treatment that would not have been appropriate in the physician's office or outpatient setting. Complications that occur during hospitalization are AEs. If a complication prolongs hospitalization or fulfils any other serious criteria, the event is serious. When in doubt as to whether 'hospitalization' occurred or was necessary, the AE should be considered serious.<br>Note that hospitalization will **not** be considered to be an SAE if it is:<br>• A routine treatment or monitoring of the disease under study, not associated with any deterioration in condition<br>• Elective or pre-planned treatment for a pre-existing condition that is unrelated to the disease under study and has not worsened since the start of study treatment<br>• For social reasons in the absence of any deterioration of the patient's general condition
**Results in persistent disability/incapacity**<br>• The term disability means a substantial disruption of a person's ability to conduct normal life functions<br>• This definition is not intended to include experiences of relatively minor medical significance such as uncomplicated headache, nausea, vomiting, diarrhea, influenza and accidental trauma (e.g. sprained ankle) which may interfere with or prevent everyday life functions but do not constitute a substantial disruption
**Is a congenital anomaly/birth defect**

Other medically important situations:

- Medical or scientific judgment should be exercised in deciding whether SAE reporting is appropriate in other situations such as important medical events that may not be immediately life-threatening or result in death or hospitalization but may jeopardize the patient or may require medical or surgical intervention to prevent one of the other outcomes listed in the above definition. These events should usually be considered serious.

  Examples of such events include invasive or malignant cancers, intensive treatment in an emergency room or at home for allergic bronchospasm, blood dyscrasias or convulsions that do not result in hospitalization, or development of drug dependency or drug abuse.

AE, adverse event; SAE, serious adverse event.

8.2.2 Reporting of SAEs/AESIs via SAE/AESI Report Form

To ensure patient safety, all SAEs/AESIs, regardless of suspected causality, occurring after informed consent date and until 28 days after the last study drug administration or patient EOS must be **reported to the Sponsor within 24 hours** of learning of its occurrence using the paper SAE/AESI Report Form.

Any additional information for the SAE/AESI including complications, progression of the initial SAE/AESI and recurrent episodes must be reported as follow-up to the original episode within 24 hours of the Investigator receiving the follow-up information. An SAE/AESI occurring during a different time interval or otherwise considered completely unrelated to a previously reported SAE/AESI should be reported separately as a new event.

All SAE/AESI occurring more than 28 days after the last study drug administration or patient EOS should be reported to the Sponsor **if the Investigator suspects a causal relationship to the study treatment**.

Information about all SAEs/AESIs is collected and recorded on the paper SAE/AESI Report Form:

- When an SAE/AESI occurs, it is the Investigator's responsibility to review all related medical documentation (e.g. hospital progress notes, laboratory and diagnostic reports) related to event and complete all applicable sections of the SAE/AESI Report Form in order to provide a clinically thorough report.
- The Investigator must assess and record the relationship of each SAE/AESI to study drug, complete the SAE/AESI Report Form in English and submit it **within 24 hours** of learning about the SAE/AESI to the Sponsor. The detailed procedure for reporting SAEs/AESIs is located in the Investigator File provided to each site. It is not acceptable for the Investigator to send photocopies of the patient's medical records to the Sponsor or designee in lieu of completing the SAE/AESI Report Form.
- There may be situations in which an AE has occurred and the Investigator has minimal information to include in the initial report to the Sponsor. However, **it is very important that the Investigator always makes an assessment of causality for every event before the initial transmission of the SAE data to the Sponsor.**
- The Investigator may change their opinion of causality in light of follow-up information and send an SAE follow-up report with the updated causality assessment.

CONFIDENTIAL
May not be used, divulged, published, or otherwise disclosed without the consent of
Molecular Partners AG, Schlieren, Switzerland

In parallel, the SAE/AESI should also be reported in the clinical database in the AE eCRF. Detailed instructions regarding the SAE submission process and requirements for signatures are found in the Investigator File provided to each site.

Suspected Unexpected Serious Adverse Reaction (SUSAR)

A serious adverse reaction is an SAE considered to be related to the study treatment.

Adverse reactions may also be expected or unexpected. An 'unexpected adverse reaction' is an adverse reaction, the nature or severity of which is not consistent with the applicable product information, which is described in the MP0317 IB. Information about expected adverse reactions already known about the study treatment can be found in the IB. Relevant safety information from the IB will be included in the patient ICF and should be discussed with the patient during the study.

If the serious adverse reaction is not documented in the IB and is thought to be related to study treatment it is considered as SUSAR. In such situations, a Sponsor Drug Safety representative may urgently require further information from the Investigator for Health Authority reporting.

In this case, the Sponsor may need to issue an Investigator Notification to inform all relevant stakeholders, including investigators of the study where the event occurred, and the regulatory authorities and ethics boards involved.

SUSARs will be collected and reported to the Competent Authorities and relevant Institutional Review Board (IRB)/Independent Ethics Committee (IEC) in accordance with the Directive 2001/20/EC or as per national regulatory requirements in participating countries.

8.3 Pregnancy

Any pregnancy occurring in a female patient or a female partner of a male patient will be collected from the first study drug administration and until 28 days after the last study drug administration or patient EOS. Patients will also be instructed to notify the Investigator after completion of the safety FU or discontinuation from the study if she (for female patients) or the patient's partner (for male patients) becomes pregnant within 3 months after the last study drug administration.

A patient who falls pregnant on study must be withdrawn from study treatment immediately.

Pregnancy should be reported to **Sponsor Drug Safety representative within 24 hours** of learning of its occurrence using a Clinical Pregnancy Form.

Sponsor Drug Safety representative will request a pregnancy case update every 3 months and within 2 weeks of the estimated delivery date. The pregnancy should be followed up to determine outcome, including spontaneous or voluntarily termination, details of the birth and the presence or absence of any birth defects, congenital abnormalities or maternal and/or new born complications. Pregnancy follow-up should be recorded on the same form and should include an assessment of the possible relationship to the study treatment of any pregnancy outcome. Any SAE experienced during pregnancy, as well as abnormal pregnancy outcomes (e.g. spontaneous abortion, fetal death, stillbirth, congenital anomalies and ectopic pregnancy) must be reported on the SAE Form.

The detailed procedure for reporting pregnancies is located in the Investigator File provided to each site.

8.4 Overdose

An overdose is the accidental or intentional use of a study treatment in an amount higher than the planned dose being studied.

CONFIDENTIAL
May not be used, divulged, published, or otherwise disclosed without the consent of
Molecular Partners AG, Schlieren, Switzerland

Definition of overdose: Any single dose of MP0317 that is ≥ 10% higher than the assigned dose.

In the event of an overdose, the Investigator should contact the Sponsor immediately and document the quantity of the excess dose as well as the duration of the overdosing in the eCRF. Decisions regarding any symptomatic treatment and/or study treatment interruptions or modifications will be made by the Investigator, in consultation with the Sponsor, based on the clinical evaluation of the patient. Overdose, per se, will not be reported as an AE/SAE although any resulting symptoms will be reported.

There is currently no known specific rescue medication for MP0317. If indicated, patients should be treated to control symptoms (e.g. hypertension, cardiac symptoms or dysfunction) with appropriate symptomatic measures (Sections 6.1 and 12.6). All medication used to control these symptoms should be recorded in the eCRF.

8.5 Dose Escalation Review Committee and Stopping Rules

A DERC, composed of all Principal Investigators, the Sponsor's medical and PK expert(s) and Drug Safety representative, an independent clinical expert and clinical research organization (CRO) representative(s), will meet to make recommendations based upon their review of patient tolerability and safety data (including DLTs, AEs, laboratory data and the BLRM summaries of DLT risk, if applicable) along with PK, pharmacodynamic and preliminary antitumor activity information available at the time of the decision. The ultimate decision remains with the Sponsor.

Administration at the next dose level cannot proceed until the Investigator receives written confirmation from the Sponsor indicating that the results of the previous dose level were evaluated and that it is safe to proceed to a higher dose level. If the MTD is not reached, the selected dose is that resulting in the predicted best therapeutic window for study treatment.

The composition, functioning (meeting frequency, data received etc.) and responsibilities of the DERC will be detailed in the DERC Charter (provided separately). All recommendations by the DERC, their rationale and decisions will be recorded in meeting minutes.

Unacceptable toxicities that may result in holding or closing enrollment will be considered when ≥2 patients in a dose escalation and/or safety expansion cohort, exhibit toxicities consistent with the definition of the same DLT per Section 5.2.5.

During the conduct of the study, any death that is considered possibly related to MP0317 will result in a study enrollment pause and, cessation of dosing equal to or higher than that dose level in which the death occurred, to allow for an expedited ad hoc evaluation by the DERC prior to further enrollment in the study.

CONFIDENTIAL
May not be used, divulged, published, or otherwise disclosed without the consent of
Molecular Partners AG, Schlieren, Switzerland

9 STATISTICAL ANALYSES

The detailed methodology for summary and statistical analyses of the data collected in this study will be documented in a Statistical Analysis Plan (SAP). The SAP will provide all data handling rules, including the management of missing values and the handling of data for withdrawn patients. The SAP will also outline protocol deviation criteria. Any deviations from the planned analyses specified or populations defined within the SAP will be justified in writing and presented in the Clinical Study Report (CSR).

The clinical database lock will occur after all study data have been reconciled (i.e. ‘cleaned’) for all patients. The SAP for the study will be finalized and signed before the database lock.

Data will be analyzed and presented by dose level and study part. In all tables, listings and figures, the dose-escalation cohorts will be reported from the lowest to the highest dose and safety expansion data will be reported by indication/treatment arm.

9.1 Sample Size Determination

Up to 6 provisional dose levels are planned for this study with at least 1 to 3 patients per dose level. It is estimated that between 17 and 30 patients will be included for the dose-escalation part of the q3w schedule, and 12 to 18 patients for the dose-escalation part of the q1w schedule. Up to 15 patients treated at the RDE (or MTD) will be included in the safety expansion part of each dosing schedule giving a total of between 32 and 78 patients. The actual number of patients will depend on the number of dose levels/cohorts that are tested, the number of patients considered non-evaluable for the DDS and who are replaced, and the safety profile seen at each dose level.

9.2 Data Handling

9.2.1 Screen Failures

Data will be listed including demography, reason for screen failure, eligibility criteria and any SAEs. These patients will not be part of any summary tables except for summarizing disposition.

9.2.2 Missing Data and Treatment Discontinuation

Due to the dose-escalation design of the first part of the study, no imputation of missing values will be done for any analysis (expect imputation for missing partial dates of AEs and concomitant medications). Reasons for discontinuation from the study treatment and study will be listed and summarized.

Additional patients will be included in the dose-escalation part of the study if fewer than 3 patients per dose level in cohort number 3 and higher are evaluable for DLT assessment. Patients in the safety expansion part will be replaced if they do not have at least one post-screening efficacy assessment and did not discontinue due to clinical PD.

9.3 Analysis Sets

9.3.1 Safety Analysis Set

The safety analysis set (SAS) will consist of all patients who received at least one dose of MP0317 and had at least one post-dose safety assessment (where the statement that a patient had no AEs constitutes a safety assessment). The SAS will be the primary population for all demography, safety,

immunogenicity, efficacy and pharmacodynamic related endpoints, except for determination of the dose-DLT relationship.

9.3.2 Dose-Determining Set

The dose-determining set (DDS) will consist of all patients in the SAS, who meet the dose-limiting toxicity evaluability criteria in Sections 5.2.5 and 5.2.6. Patients who do not meet these minimum treatment and safety evaluation requirements will be regarded as ineligible for inclusion in the DDS and will be replaced if needed until the minimum number of patients required for evaluation is reached. The DDS will be used in the BLRM to estimate the dose-DLT relationship in the dose-escalation part of the study.

9.3.3 Pharmacokinetics Analysis Set

The PK set consists of all patients who received at least one dose of MP0317 and had at least one post-dose PK measurement.

9.4 Primary Analysis

9.4.1 Dose-Escalation

An adaptive BLRM guided by the EWOC principle will be used in the dose-escalation part to determine the RDE (or MTD). The use of Bayesian response adaptive models for phase 1 studies has been advocated by the European Medicines Agency (EMA) Guideline on Clinical Trials in small Populations[44] and by Rogatko[45] and is one of the key elements of the Food and Drug Administration (FDA)'s Critical Path Initiative.

9.4.1.1 Dose-Finding of Single Agent MP0317

A 2-parameter BLRM[41] will be used for dose-escalation. All information currently available about the dose-DLT relationship of MP0317 is summarized in a prior distribution (Table). This prior distribution is then updated after each cohort of patients with all of the DLT data available in the DDS from the current study. Once updated, the distribution summarizes the probability that the true rate of DLT for each dose lies in the following categories:

- [0,16%) underdosing
- [16%,33%) targeted toxicity
- [33%,100%] excessive toxicity

The EWOC principle[41, 42] mandates that any dose of MP0317 that has more than a 25% chance of being in the excessive toxicity category is not considered for the next cohort. After a clinical synthesis of the available toxicity information (including AEs that are not DLTs), PK, pharmacodynamic and efficacy information as well as the recommendations from the Bayesian model, the DERC will determine the dose level for the next cohort at a dose-escalation meeting. In all cases, where there is a change in dose level to be administered to the next enrolled patients, a new model will be defined using a Meta-Analytic-Predictive prior, based on the observed data.

The frequency of DLTs will be tabulated by dose for patients in the dose-escalation part and information about the DLTs will be listed by dose.

CONFIDENTIAL
May not be used, divulged, published, or otherwise disclosed without the consent of
Molecular Partners AG, Schlieren, Switzerland

9.4.1.2 Bayesian Logistic Regression Model for MTD Determination

The objective of the design is to determine the MTD defined as the highest dose with less than 25% risk of the true DLT rate being above 33%. The dose-finding will be guided by a Bayesian 2-parameter logistic regression model with overdose control. These designs have been shown to be superior regarding the precision of MTD determination compared to 3+3 designs and have been particularly endorsed by the FDA.

The model is formulated as follows:

$\text{logit}(p(d)) = \log(\alpha) + \beta^*\log(d/d^*)$,

where $\text{logit}(p) = \log(p/(1-p))$. p(d) represents the probability of having a DLT in the first cycle at dose d, $d^* = 1$ mg/kg is the reference dose, allowing for the interpretation of α as the odds of a DLT at dose d^*, and $\theta = (\log(\alpha), \log(\beta))$ with $\alpha, \beta > 0$ is the parameter vector of the model. Standardized doses will be used such that one of the doses (d^*) equals 1, e.g. doses are rescaled as d/d^*. As a consequence, α is equal to the odds of the probability of toxicity at d^*.

Since a Bayesian approach is applied, a prior distribution $\pi(\theta)$ for the unknown parameter vector θ needs to be specified. This prior distribution will be specified as a normal distribution, i.e.

$\pi(\theta) = MVN(\mu, \Sigma)$

mean vector μ and covariance matrix Σ, with

$$\Sigma_i = \begin{pmatrix} \sigma_{i,11}^2 & \sigma_{i,11}\sigma_{i,22}\rho_i \\ \sigma_{i,11}\sigma_{i,22}\rho_i & \sigma_{i,22}^2 \end{pmatrix}$$

9.4.1.3 Prior Derivation for Q3W

The starting dose of MP0317 will be 0.03 mg/kg, which is presumed to be at the lower end of the pharmacologically active dose range. No critical toxicities were observed in the cynomolgus monkey toxicity studies up to the maximum applied MP0317 dose level of 100 mg/kg. It is therefore highly unlikely that a severe toxicity defined as DLT in this study would occur at this starting dose, leading to the prior assumption that the median DLT rate at 0.03 mg/kg is 1%. On the other hand, assuming dose-proportionality in humans using a cautious approach, the median DLT rate at the highest planned dose = 10 mg/kg was therefore assumed to be 20%.

Table 23. Summary of Prior Distribution

Mean Vector	STD Vector	Correlation
-2.685; -0.594	2; 0.5	0

STD, standard deviation.

A summary of the prior probabilities of DLT at different doses, as well as the corresponding probability of under, targeted and overdosing, are shown in Table 24. Graphically, the prior medians with accompanying 95% credible intervals (CrI) are shown in Figure 3. The uncertainty around the medians is large, showing the low amount of in-human information this prior provides.

Table 24. Prior Probabilities of DLT at Selected Doses

Dose (mg/kg)	Probability of True DLT Rate in					Quantiles		
	[0–0.16)	[0.16–0.33)	[0.33–1]	Mean	STD	2.5%	50%	97.5%
0.01	0.949	0.031	0.02	0.032	0.086	0	0.004	0.286
0.03	0.922	0.046	0.032	0.046	0.105	0	0.008	0.382
0.10	0.874	0.072	0.054	0.070	0.133	0	0.017	0.511
0.30	0.804	0.105	0.091	0.103	0.165	0.001	0.032	0.646
1.00	0.693	0.141	0.166	0.158	0.209	0.002	0.065	0.783
**3.00**	0.563	0.171	0.267	0.230	0.254	0.003	0.121	0.885
**10.00**	0.425	0.174	0.401	0.326	0.299	0.005	0.222	0.956

Doses printed in bold type do not meet the overdose criterion, P(overdose) < 0.25.

DLT, dose-limiting toxicity; P, probability; STD, standard deviation.

Figure 3. Prior Medians and 95% Credible Intervals

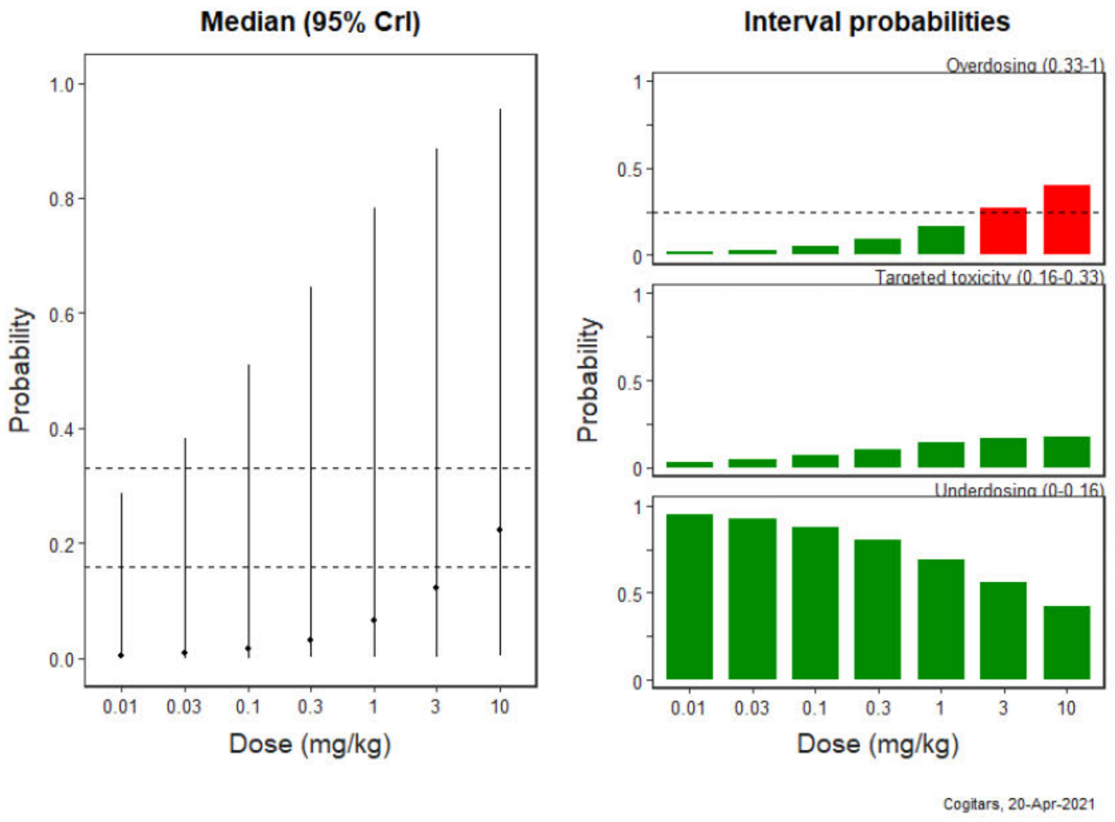

CrI, credible intervals.

The MTD may be considered reached if both of the following criteria are fulfilled:

- The posterior probability of the true DLT rate in the target interval (16%-33%) of the MTD above 50% with less than 25% risk of the true DLT rate being above 33%
- At least 6 patients have been treated at MTD, including the final RDE/MTD cohort

9.4.1.4 Prior Derivation for Q1W

For the potential new dossing schedule (Q1W), the prior will be derived on the accumulated data prior to the dose escalation of the new schedule. The prior derivation, data scenarios and operating characteristics will be documented in the SAP.

9.4.1.5 Statistical Model Assessment

The model was assessed using two different metrics:

- Hypothetical data scenarios: for various potential data constellations as they could occur in the current study, the maximal next doses as allowed by the model and by the 200% escalation limit are investigated. Data scenarios thus provide a way to assess the 'on-study' behavior of the model.
- Simulated operating characteristics: these illustrate for different assumed true dose-toxicity relationships, how often a correct dose would be declared as MTD by the model. They are a way to assess the 'long-run' behavior of the model.

In summary, the model showed very good behavior as assessed by these metrics. More details can be found in Appendix 12.8.

9.5 Patient Demography and Other Screening Characteristics

Demographic information, medical history, prior medication, cancer history, prior anticancer treatments and other screening data will be listed and summarized using descriptive statistics for numerical data and contingency tables for categorical data. Medical history and prior medication will be listed.

The reported medical history terms will be assigned a standardized preferred term using Medical Dictionary for Regulatory Activities (MedDRA) and summarized by MedDRA system organ class (SOC) and preferred term.

Prior medication and prior anticancer treatments will be coded by World Health Organization (WHO) Drug Dictionary to Anatomical Therapeutic Classification (ATC) terms. Prior anticancer treatments will be summarized by cohort and total. Prior medications will be summarized by ATC level and preferred drug name and listed.

Analyses will be performed in the SAS.

9.6 Study Treatment and Concomitant Medication

Exposure to study treatment will be summarized with descriptive statistics for the total number of infusions received. The total duration on study treatment (first to last study drug administration) will also be derived and summarized. The number of infusions with interruptions and the duration of interruptions will be summarized by cohorts and reason for modification.

CONFIDENTIAL
May not be used, divulged, published, or otherwise disclosed without the consent of
Molecular Partners AG, Schlieren, Switzerland

Concomitant medication will be coded by WHO Drug Dictionary to ATC terms. Concomitant medications will be summarized by ATC level and preferred drug name and listed.

9.7 Safety Analyses

9.7.1 Adverse Events

Summary tables and listings will be provided for all reported treatment-emergent AEs (TEAEs), defined as AEs that start during or after the first study drug administration. The reported AE terms will be assigned a standardized preferred term using MedDRA. TEAEs will be summarized based on the number and percentage of patients experiencing the event by MedDRA SOC and preferred term.

The causal relationship between the occurrence of an AE and the study treatment will be judged by the Investigator. In the event that a patient experiences repeat episodes of the same AE, the event with the highest severity grade and strongest causal relationship to study treatment will be used for incidence tabulations.

Tabular summaries will be provided as follows (where severity is based on NCI CTCAE v5.0, and relation to study treatment as described in Section 8.1.1):

- All TEAEs (by SOC and preferred term, severity and relationship to study treatment)
- TEAEs by relationship (related, irrespective of relationship) to study treatment and maximum severity grade
- TEAEs with action on study drug administration such as drug administration delays or infusion interruptions
- TEAEs with action of study treatment discontinued
- Grade ≥ 3 TEAEs
- Serious TEAEs by relationship (related, irrespective of relationship) to study treatment

Listings will be provided for SAEs (including those observed during the screening period), Grade ≥ 3 TEAEs, TEAEs leading to study drug administration delay, infusion interruption, discontinuation and death.

All deaths occurring after the informed consent date and until 4 weeks after the last study drug administration or patient EOS will be reported in a listing, which will include the primary cause of death and the number of days between the date of the last study drug administration and death.

Tolerability will be analyzed in terms of study drug administration delays and infusion interruptions due to AEs. Reasons for delays and interruptions will be listed and summarized.

9.7.2 Laboratory Parameters

Safety laboratory parameters will be graded by NCI CTCAE v5.0. For parameters without a severity grading scale, values will be classified into low/normal/high based on laboratory normal ranges. Each parameter will be presented by descriptive statistics at each visit including change from screening. Shift tables for grades and normal ranges will be presented. All laboratory parameters will be listed. A separate listing for abnormal laboratory results (Grade ≥ 3 and low/high values) will be presented.

Directional shifts in laboratory parameter grades (comparing grade at screening with worst post-screening grade) will be analyzed using standard shift tables, presenting the number and proportion of patients and their maximum grade shift. For parameters without a severity grading scale, the shift

table will present directional shifts from screening to above or below the laboratory standard normal range using the maximum increase and/or decrease observed throughout the course of treatment/observation.

9.7.3 Vital Signs

Vital signs will be listed and summarized by descriptive statistics at each visit including change from screening.

9.7.4 Electrocardiogram

Local-read ECG data will be listed overall and a separate listing for any clinically significant findings will be provided. Change from screening in QT intervals by cohort will be summarized for each visit as well as change from screening in all other ECG parameters. The worst change from screening will be summarized. The frequency and percentage of patients with notable ECG changes and newly occurring qualitative ECG abnormalities will be tabulated by cohort.

9.8 Pharmacokinetic Analyses

Individual and mean serum MP0317 concentration versus time data will be tabulated and plotted by dose level. The PK of MP0317 will be summarized by estimating total AUC, C_{max}, C_{min}, CL, V_{ss} and $t_{1/2}$ (as appropriate for data collected). No formal statistical analysis beyond descriptive statistics is planned. For each PK parameter, individual and mean data and summary statistics (including number of patients, arithmetic mean, geometric mean [for t_{max} and time to last measurable serum concentration [t_{last}] no geometric mean will be calculated]), standard deviation (STD), confidence value (CV), median, minimum and maximum) will be presented. Estimates for these parameters will be tabulated and summarized (mean, standard deviation and coefficient of variation). Inter-patient variability and drug accumulation will be evaluated.

Analyses will be performed in the PK set. The Sponsor may prepare a separate analysis plan and report for the PK analysis. Final PK evaluation will be performed after the end of the study and will be defined in more detail in the SAP.

9.9 Immunogenicity Analyses

Incidence of ADA response and the potential correlation with PK, pharmacodynamic and safety parameters may be assessed. Immunogenicity parameters will be summarized by descriptive statistics and listed.

The Sponsor may prepare a separate analysis plan and report for the immunogenicity analysis. Final immunogenicity evaluation will be performed after the end of the study and will be defined in more detail in the SAP.

9.10 Efficacy Analyses

All efficacy analyses will be done according to investigator-assessed response criteria.

Overall response rate (ORR) will be estimated for each cohort/dose level evaluated.

ORR is defined as the proportion of patients with best overall response of CR or PR as determined by the Investigator using RECIST v1.1 and iRECIST, as appropriate to tumor type. RECIST v1.1

(Appendix 12.3), will be used for the primary response evaluation and iRECIST (Appendix 12.4) will be used to capture the phenomenon of pseudoprogression, which requires PD confirmation.

Waterfall plots will be used to depict graphically efficacy for individual patients with their maximum percentage decrease from screening in the sum of longest diameters of target lesions.

For all response assessments, swimmers' plots will be presented. All response assessments will be listed.

Patients with no post-screening efficacy measurement due to PD, will be assessed as early progressors and will count in the denominator of the ORR.

Similarly, the change in tumor burden over time for individual patients and the occurrence of clinical outcomes of interest (e.g. tumor response, PD, treatment discontinuation, death) will be displayed.

Disease control rate (DCR) is defined as the BOR of CR, PR or SD lasting 4 or more weeks following date of first study drug administration. The point estimates of the DCR and the 2-sided 90% exact binomial confidence interval (CI) will be presented by cohort.

Duration of response (DOR) will be calculated for patients who achieve CR or PR. For such patients, DOR is defined as the time from the start date of CR or PR (whichever response status is observed first) to the first date that either recurrent or PD is objectively documented or death.

Time to progression (TTP) is defined as the time from the date of first study drug administration to documented PD.

Progression-free survival (PFS) is defined as the time from the date of first study drug administration to documented PD or death, whichever occurs first. Patients with no event will be censored at the time of the last available tumor assessment for PFS.

Overall survival (OS) is defined as the time from the date of first study drug administration to the date of death, irrespective of cause, and will be censored at the last date the patient was known to be alive.

DOR, PFS and OS will be summarized descriptively using the Kaplan-Meier method with 95% CIs calculated using Greenwood's formula. Median follow-up for each endpoint will be estimated according to the Kaplan-Meier estimate of potential follow-up[46]. The right-censored criteria will be described in the SAP, and the date of PD or censoring will be determined based on described conventions.

Exploratory analyses of selected antitumor activity endpoints may be performed based on subgroups of patients in the SAS.

9.11 Pharmacodynamic Analyses

Pharmacodynamic analyses will include assessments of biomarkers in blood, tumor and skin tissue. Pre-treatment, on-treatment, and post-treatment levels of biomarkers will be reported using descriptive statistics and presented graphically. Changes in pharmacodynamic and potential predictive biomarkers will be listed by dose, cohort and response status. Detailed analyses will be discussed in the SAP.

9.12 Exploratory Analyses

Additional exploratory PK and biomarker analyses will be conducted as appropriate. Detailed analyses will be discussed in the SAP or the Sponsor may prepare a separate analysis plan and report.

CONFIDENTIAL
May not be used, divulged, published, or otherwise disclosed without the consent of
Molecular Partners AG, Schlieren, Switzerland

9.13 Interim Analyses

Each dose-escalation step is considered to be an interim analysis. The BLRM will be updated with the respective number of patients treated and the number of DLTs observed in the last cohort. The updated model will then give a statistical recommendation for the next escalation step. In addition, a risk-benefit assessment that includes a comprehensive analysis of available safety and clinical information will be done to decide on the next escalation steps.

The SAP will describe the planned interim analyses in greater detail.

CONFIDENTIAL
May not be used, divulged, published, or otherwise disclosed without the consent of
Molecular Partners AG, Schlieren, Switzerland

10 QUALITY

10.1 Data Quality Control and Quality Assurance

All patient data relating to the study will be recorded in eCRF unless defined differently with the site. The Investigator is responsible for verifying that data entries are accurate and correct by physically or electronically signing the eCRF.

The Investigator must maintain accurate documentation (source data) that supports the information entered in the eCRF.

The Investigator must permit study-related monitoring, audits, IRB/IEC review and regulatory agency inspections and provide direct access to source data documents.

The Sponsor or designee is responsible for the data management of this study including quality checking of the data. Study CRAs will perform ongoing source data verification to confirm that data entered into the eCRF by authorized site personnel are accurate, complete and verifiable from source documents, that the safety and rights of patients are being protected and that the study is being conducted in accordance with the currently approved protocol and any other study agreements, International Council for Harmonisation of Technical Requirements for Pharmaceuticals for Human Use (ICH) Good Clinical Practice (GCP) and all applicable regulatory requirements.

10.2 Study Monitoring Requirements

Site visits will be conducted by an authorized Sponsor representative to inspect study data, patient medical records and eCRFs. The Principal Investigator will permit Sponsor CRAs/representatives and collaborators, regulatory agencies, IRBs/IECs and the respective national or local health authorities to inspect facilities and records relevant to this study.

10.3 Case Report Form Completion

An eCRF will be used at the clinical study site to collect study data for enrolled patients. When data are available, authorized clinical study site personnel will carefully and accurately record the data in the eCRF. Sites must ensure that all source documents are maintained according to ICH/GCP guidance and support the data that are entered into the eCRFs.

The eCRF data will be captured in a validated system according to procedures that comply with the appropriate ICH/GCP guidelines. The eCRFs will be reviewed and signed by the Principal Investigator or designee who is clinically qualified and identified on the delegation log as an individual who can sign-off for the eCRFs.

10.4 Source Documents

Source documents provide evidence for the existence of the patient and substantiate the integrity of the data collected. Source documents are filed at the Investigator’s site. A definition of what constitutes source data can be found in the site-specific source data agreement.

Data reported in the eCRF that are transcribed from source documents must be consistent with the source documents or the discrepancies must be explained. The Investigator may need to request previous patient medical records or transfer records, depending on the study. Current patient medical records must be available.

CONFIDENTIAL
May not be used, divulged, published, or otherwise disclosed without the consent of
Molecular Partners AG, Schlieren, Switzerland

When clinical observations are entered directly into an Investigator site's computerized medical record system (i.e. in lieu of original hardcopy records), the electronic record can serve as the source document if the system has been validated in accordance with applicable requirements pertaining to computerized systems used in clinical research. An acceptable computerized data collection system (for clinical research purposes):

- Allows data entry only by authorized individuals
- Prevents the deletion or alteration of previously entered data and provides an audit trail for such data changes (e.g. modification of file)
- Protects the database from tampering and
- Ensures data preservation.

If a site's computerized patient medical record system is not adequately validated for the purposes of clinical research (as opposed to general clinical practice), applicable hardcopy source documents must be maintained to ensure that critical protocol data entered into the eCRFs can be verified.

10.5 Data Protection

The Sponsor will assign patients a unique identifier. Any patient medical records or datasets that are transferred to the Sponsor will contain the identifier only; patient names or any information which would make the patient identifiable will not be transferred. If the patient's name appears on any document (e.g. laboratory report), it must be eliminated on the copy of the document supplied to the Sponsor.

Study data stored on a computer will be kept in accordance with local data protection laws. The patient must be informed that the Sponsor, in accordance with local data protection law, will use their personal study-related data. The level of disclosure must also be explained to the patient.

The patient must be informed that their patient medical records may be examined by clinical quality assurance auditors or other authorized personnel appointed by the Sponsor, by appropriate IRB/IEC members and by inspectors from regulatory authorities.

10.6 Disclosure of Data

Patient medical information obtained by this study is confidential and may be disclosed to third parties only as permitted by the ICF (or separate authorization to use and disclose personal health information) signed by the patient or unless permitted or required by law.

Medical information may be given to a patient's personal physician or other appropriate medical personnel responsible for the patient's welfare for treatment purposes.

Data generated by this study must be available for inspection upon request by representatives of appropriate regulatory agencies, national and local health authorities, Sponsor CRAs/representatives and collaborators and the IRB/IEC for each study site, if appropriate.

10.7 Data Management

Clinical data management will be performed by the Sponsor or designee according to procedures described in a comprehensive data management plan.

CONFIDENTIAL
May not be used, divulged, published, or otherwise disclosed without the consent of
Molecular Partners AG, Schlieren, Switzerland

The data management plan will include procedures for processing the data from this study and will describe the responsibilities of the Sponsor and designee when clinical data management is provided by an external vendor. In particular, the data management plan will include a list of the standard operating procedures that apply to this study.

10.8 Study Documentation, Record Keeping and Retention of Documents

The Investigator must retain records and documents, including signed ICF, pertaining to the conduct of this study. The ICH Guideline for GCP (Section 4.9 of the ICH GCP guideline E6(R2)) requires that records and documents pertaining to the conduct of this study and the distribution of study treatment, including ICFs, laboratory test results and medication inventory records, must be retained by the Principal Investigator

- For 2 years after the last marketing application approval in an ICH region,
- Until at least 2 years have elapsed since formal discontinuation of clinical development of the study treatment or
- For a longer period as required by national or local law

No records may be destroyed without the written approval of the Sponsor. No records may be transferred to another location or party without written notification to the Sponsor.

CONFIDENTIAL
May not be used, divulged, published, or otherwise disclosed without the consent of
Molecular Partners AG, Schlieren, Switzerland

11 ETHICAL CONSIDERATIONS AND ADMINISTRATIVE PROCEDURES

11.1 Regulatory and Ethical Compliance

11.1.1 Protocol and Protocol Amendments

The protocol and informed consent documents must be reviewed and approved by the IRB/IEC. The study will not be initiated at a site until appropriate written IRB/IEC approval of the protocol, ICF and all recruiting materials (if applicable) is obtained by the Investigator. Copies should be reviewed and approved by the Sponsor prior to submission to the IRB/IEC. The Investigator will submit periodic reports on the progress of the study as required by the IRB/IEC, in accordance with applicable governmental regulations, and in agreement with the policy established by the Sponsor. In addition, the Investigator will inform the IRB/IEC of any protocol amendments and administrative changes, and will obtain appropriate written IRB/IEC approval of all protocol amendments.

This study will be conducted in accordance with the ICH E6 Guideline for GCP, the Declaration of Helsinki, the European Union (EU) Clinical Trials Directive 2001/20/EC, the GCP Directive 2005/28/EC, and applicable national and local laws and guidelines.

11.1.2 Informed Consent Procedure

The Investigator or their representative will explain the nature of the study to the patient and answer all questions regarding the study.

Patients must be informed that their participation is voluntary. Patients will be required to sign a statement of informed consent (i.e., the ICF) that meets the requirements of local regulations, ICH guidelines and, where applicable, the IRB/IEC or study site.

The patient medical record must include a statement that written informed consent was obtained before the patient can participate in the study and the date the written consent was obtained. The authorized person obtaining the informed consent must also sign the ICF.

In the event of an amendment to the ICF, patients must be re-consented to the latest version of the ICF(s) during their participation in the study.

A copy of the ICF(s) must be provided to the patient.

11.2 Responsibilities of the Investigator

The Investigator is responsible for:

- Providing written summaries of the study status to the IRB/IEC annually or more frequently in accordance with the requirements, policies and procedures established by the IRB/IEC.
- Notifying the IRB/IEC of any SAEs or other significant safety findings as required by IRB/IEC procedures.
- Keeping a record of all communication with the IEC/IRB and the health authorities.
- Overall conduct of the study at the site and adherence to the requirements of the ICH guidelines and the IRB/IEC.
- Ensuring that all persons assisting with the study are adequately qualified, informed about the protocol, any amendments to the protocol, the study treatment and their study-related duties and functions.

CONFIDENTIAL
May not be used, divulged, published, or otherwise disclosed without the consent of
Molecular Partners AG, Schlieren, Switzerland

- Maintaining a list of Sub-Investigators and other appropriately qualified persons to whom they have delegated significant study-related duties.

11.2.1 Protocol Adherence

This protocol defines the study objectives, the study procedures and the data to be collected about study patients. Additional assessments required to ensure safety of patients should be performed as deemed necessary on a case-by-case basis. Under no circumstances is an Investigator allowed to collect additional data or conduct any additional procedures for any research-related purpose involving any investigational drugs under this protocol.

Investigators agree to be diligent in avoiding protocol deviations. If an Investigator feels a protocol deviation would improve the conduct of the study, this deviation must be considered as a protocol amendment. Unless such an amendment is agreed upon by the Sponsor and approved by the IRB/IEC and health authorities, where required, it cannot be implemented unless necessary to eliminate immediate hazard to the patient.

11.2.2 Protocol Modification and Amendments

No modifications to the protocol should be made without prior approval of the IRB/IEC.

When circumstances require an urgent departure from procedures defined in the protocol, the Investigator will contact the Sponsor to discuss the planned course of action. Any departures from the protocol must be fully documented in the source documentation and in the protocol deviation log.

Any protocol amendments will be prepared by the Sponsor. Protocol amendments will be submitted to the IRB/IEC and to regulatory authorities where required by and in accordance with EU and local regulatory requirements. Where approval by the IRB/IEC and/or regulatory authorities is required, the changes will not be implemented until this approval is received, except where the change is required as an urgent safety measure as defined by ICH guidelines.

11.2.3 Coordinating Investigator

If a Coordinating Investigator is assigned for the clinical study, they will be designated by mutual agreement.

11.3 Study Termination

The Sponsor will terminate this study following completion of the study objectives or earlier if deemed necessary.

The Sponsor reserves the right to terminate the study or dosing schedules thereof at any time. If the Sponsor becomes aware of information on matters concerning the quality, efficacy or safety of the study treatment or any other study treatment information that may affect proper conduct of the clinical study, the Sponsor may terminate the study. The Sponsor will send a written notice of the termination along with the reasons to the competent authorities, the IEC/IRB and the Investigators. If an Investigator or the Investigator’s IEC/IRB intends to terminate participation in the study, the Investigator must immediately inform the Sponsor and provide the reason for it.

11.4 Site Discontinuation

The Sponsor has the right to close a site at any time. Reasons for closing a site may include but are not limited to:

CONFIDENTIAL
May not be used, divulged, published, or otherwise disclosed without the consent of
Molecular Partners AG, Schlieren, Switzerland

- Excessively slow recruitment
- Poor protocol adherence
- Inaccurate or incomplete data recording
- Non-compliance with the ICH guideline for GCP
- No study activity (i.e. all patients have completed and all obligations have been fulfilled)

11.5 Publication of Study Protocol and Results

The results of this study may be published and/or presented at scientific meetings. The Sponsor encourages publication of clinical study data in reputable peer reviewed journals. Authorship will be discussed and agreed in advance in accordance with standard editorial and ethical practice. Prior to publication, the Sponsor will review and comment before its submission to the journal. In cases where the Sponsor considers that the proposed publication contains patentable material or information which should be protected as valuable confidential information, the Sponsor reserves the right to delay submission to the journal until patent applications have been filed and/or require the deletion of the confidential information from the proposed publication.

Authorship will be determined by mutual agreement and in line with International Committee of Medical Journal Editors (ICMJE) authorship guidelines.

11.6 Dissemination of Clinical Study Data

Dissemination of the results of this study will be in accordance with current regulatory guidance.

11.7 Audits and Inspections

The Sponsor may conduct audits of clinical study activities in accordance with internal standard operating procedures to evaluate compliance with the principles of GCP and ICH related guidelines.

A regulatory authority may also wish to conduct an inspection (during the study or after its completion). Should regulatory authorities request an inspection, the Investigator must inform the Sponsor or designee immediately that such request has been made.

11.8 Liability, Insurance and Financial Disclosures

Liability and insurance provisions for this study are provided separately. The Sponsor has taken out an insurance covering their civil responsibility. A copy of the country-specific insurance certificates will be maintained in the Investigator File and Trial Master File. Details of the insurance will be made available to patients in the ICF.

Investigators and Sub-Investigators will provide the Sponsor with sufficient, accurate financial information in accordance with local regulations to allow the Sponsor to submit complete and accurate financial certification or disclosure statements to the appropriate regulatory authorities.

CONFIDENTIAL
May not be used, divulged, published, or otherwise disclosed without the consent of
Molecular Partners AG, Schlieren, Switzerland

12 APPENDICES

12.1 Appendix: ECOG Performance Status

Grade	ECOG Performance Status
0	Fully active, able to carry on all pre-disease performance without restriction
1	Restricted in physically strenuous activity but ambulatory and able to carry out work of a light or sedentary nature, e.g. light house work, office work
2	Ambulatory and capable of all self-care but unable to carry out any work activities. Up and about more than 50% of waking hours.
3	Capable of only limited self-care, confined to bed or chair more than 50% of waking hours
4	Completely disabled. Cannot carry on any self-care. Totally confined to bed or chair.
5	Dead

ECOG, Eastern Cooperative Oncology Group.

Source: Oken[47]

CONFIDENTIAL
May not be used, divulged, published, or otherwise disclosed without the consent of
Molecular Partners AG, Schlieren, Switzerland

12.2 Appendix: Contraceptive Guidance

Females of Childbearing Potential (FCBP)

A female is considered fertile following menarche and until becoming postmenopausal unless permanently sterile. Permanent sterilization methods include hysterectomy, bilateral salpingectomy and bilateral oophorectomy.

Females in the following categories are not considered FCBP:

1. Premenopausal female with one of the following:
    a. Documented hysterectomy
    b. Documented bilateral salpingectomy
    c. Documented bilateral oophorectomy

    Note: Documentation can come from the site personnel by review of patient medical records, physical examination or medical history interview.
2. Postmenopausal female:
    a. A postmenopausal state is defined as no menses for 12 months without an alternative medical cause. A high follicle stimulating hormone (FSH) level in the postmenopausal range may be used to confirm a postmenopausal state in females not using hormonal contraception or hormonal replacement therapy (HRT). However, in the absence of 12 months of amenorrhea, a single FSH measurement is insufficient.
    b. Females on HRT and whose menopausal status is in doubt will be required to use one of the non-hormonal highly effective contraception methods if they wish to continue their HRT during the study. Otherwise, they must discontinue HRT to allow confirmation of postmenopausal status before study enrollment.

Contraception Guidance

Female Patients

Highly effective contraceptive methods that are user dependent *(failure rate of < 1% per year when used consistently and correctly [a])*

- Combined (estrogen and progestin-containing) hormonal contraception associated with inhibition of ovulation [b]
    - Oral
    - Intravaginal
    - Transdermal
- Progestin-only hormonal contraception associated with inhibition of ovulation [b]
    - Oral
    - Injectable

Highly effective methods that are user independent (*failure rate of < 1% per year)*

- Implantable progestin-only hormonal contraception associated with inhibition of ovulation [b]
- Intrauterine device
- Intrauterine hormone-releasing system
- Bilateral tubal occlusion

CONFIDENTIAL
May not be used, divulged, published, or otherwise disclosed without the consent of
Molecular Partners AG, Schlieren, Switzerland

- Vasectomized partner: If the partner is the sole male sexual partner of the FCBP and the absence of sperm has been confirmed. If not, an additional highly effective method of contraception should be used.
- Sexual abstinence: Defined as refraining from heterosexual intercourse during the treatment period and for at least 3 months after last dose of study treatment. The reliability of sexual abstinence needs to be evaluated in relation to the duration of the study and the preferred and usual lifestyle of the patient.

  Periodic abstinence (e.g. calendar, ovulation, symptothermal or postovulation methods) and withdrawal are **not** acceptable methods of contraception

[a] Typical use failure rates may differ from those when used consistently and correctly. Use should be consistent with local regulations regarding the use of contraceptive methods for patients participating in clinical studies.

[b] Hormonal contraception may be susceptible to interaction with certain concomitant medications, which may reduce the efficacy of the contraceptive method. In this case, 2 highly effective methods of contraception may be considered during the treatment period and for at least 3 months after the last study drug administration.

Male Patients

Male patients with female partners of childbearing potential are eligible to participate in the study if the following criteria apply throughout the study and for 3 months after the last study drug administration:

- Are abstinent from penile-vaginal intercourse as their usual and preferred lifestyle (abstinent on a long-term and persistent basis) and agree to remain abstinent for duration of study and for at least 3 months after the last dose of the study treatment.
- Female partner is using a highly effective contraceptive method.
- Agree to use a male condom plus an additional method with a failure rate of < 1% per year as described above when having penile-vaginal intercourse with a FCBP.
- Men with a pregnant or breastfeeding partner must agree to remain abstinent from penile-vaginal intercourse or use a male condom during each episode of penile penetration during the study.
- Refrain from donating sperm for the duration of the study and for at least 3 months after the last dose of the study treatment.

CONFIDENTIAL
May not be used, divulged, published, or otherwise disclosed without the consent of
Molecular Partners AG, Schlieren, Switzerland

12.3 Appendix: Response Evaluation with RECIST v1.1

Table 25. RECIST v1.1 Guidelines for Tumor Response

**Disease Response Criteria for Target and Non-target Lesions**	
**Evaluation of Target Lesions**	
Complete Response (CR)	Disappearance of all target lesions. Pathologic nodes must have a reduction in the short axis to < 10 mm.
Partial Response (PR)	At least a 30% decrease in the sum of the diameters of target lesions, taking as reference the baseline sum diameters.
Stable Disease (SD)	Neither sufficient shrinkage to qualify for PR nor sufficient increase to qualify for PD, taking as reference the smallest sum LD since the treatment started.
Progressive Disease (PD)	At least a 20% increase in the sum of the diameters of target lesions, taking as reference the smallest sum on study recorded since the treatment started or the appearance of one or more new lesions. In addition to 20% increase, the sum must also demonstrate an absolute increase of at least 5 mm.
**Evaluation of Non-target Lesions**	
Complete Response (CR)	Disappearance of all non-target lesions and normalization of tumor marker level.
Incomplete Response/Stable Disease (SD)	Persistence of one or more non-target lesion(s) or/and maintenance of tumor marker level above the normal limits.
Progressive Disease (PD)	Appearance of one or more new lesions and/or unequivocal progression of existing non-target lesions.

CR, complete response; LD, longest diameter; PD, progressive disease; PR, partial response; SD, stable disease.

Table 26. RECIST v1.1 Overall Response Criteria

**Patients with Target and Non-target Lesions**			
**Target Lesions**	**Non-Target Lesions**	**New Lesions**	**Overall Response**
CR	CR	No	CR
CR	Non-CR/Non-PD	No	PR
CR	Not evaluated	No	PR
PR	Non-PD or not all evaluated	No	PR
SD	Non-PD or not all evaluated	No	SD
Not evaluated	Non-PD	No	NE
PD	Any	Yes or no	PD
Any	PD	Yes or no	PD
Any	Any	Yes	PD

**Patients with Non-target Lesions Only**		
**Non-Target Lesions**	**New Lesions**	**Overall Response**
CR	No	CR
Non-CR/Non-PD	No	Non-CR/Non-PD
Not all evaluated	No	NE
Unequivocal PD	Yes or No	PD
Any	Yes	PD

CR, complete response; NE, not evaluable; PD, progressive disease; PR, partial response; SD, stable disease.

Source: Eisenhauer[5]

12.4 Appendix: Evaluation and Guidance Using iRECIST

After RECIST v1.1 defined progressive disease (PD) is identified at any time point, a patient may continue treatment if clinically stable, at the discretion of the Investigator. Obtain repeat imaging 4 to 8 weeks later to confirm PD. If PD is not confirmed, treatment can continue.

If PD is confirmed and the patient is still deriving a clinical benefit, contact the Sponsor to discuss continuing treatment, and if granted, document the Sponsor approval to continue to treat beyond confirmed PD per protocol. At this first PD visit, enter both RECIST v1.1 response and iRECIST response. At all following visits, enter iRECIST response data only.

For visits after the visit when radiographic PD is first seen, complete the eCRF for treatment beyond radiographic progression.

Once immune confirmed PD (iCPD) occurs, treatment should generally be discontinued.

Table 27. iRECIST Response Criteria Definitions

**Response Category**	**Criteria**
Immune Complete Response (iCR)	All lesions resolved
Immune Partial Response (iPR)	At least a 30% decrease in the sum of the diameters of target lesions, taking as reference the baseline sum diameters.
Immune Progressive Disease (iPD)	At least a 20% increase in the sum of the diameters of target lesions, taking as reference the smallest sum on study recorded since the treatment started or the appearance of one or more new lesions. In addition to 20% increase, the sum must also demonstrate an absolute increase of at least 5 mm.
	Wait up to 12 weeks to confirm PD to account for flare
Immune Stable Disease (iSD)	Does not meet other criteria

iCPD, immune confirmed progressive disease; iCR, immune complete response; iPR, immune partial response; iSD, immune progressive disease; PD, progressive disease.

Table 28. iRECIST Guidelines for Progressive Disease Evaluation

**Any factor below on the confirmatory scan (after iUPD) indicates iCPD**	
Target Lesions	SOD increases ≥ 5 mm from iUPD TL<br>SOD does not have to increase 20% from iUPD
Non-Target Lesions	Any further increase in size (qualitative assessment) from an iUPD NTL<br>Does not have to meet 'unequivocal' standard
New Lesions	There are any additional new lesions<br>or<br>Size of previously detected new lesions increases<br>- Target NL: NL iSOD increases ≥ 5 mm<br>- NT NL: Any significant growth
**Any factor below indicates progression (iUPD) after iSD/iPR/iCR**	
Target Lesions	SOD crosses PD threshold (1st time or again)<br>Based on the nadir (i.e. smallest value ever)
Non-Target Lesions	New unequivocal progression<br>or<br>If already showed PD, and did not regress, any growth
New Lesions	New lesions of any size appear for the first time, or additional new lesions appear<br>If new lesions had previously appeared and are still present,<br>Any growth<br>- Target NL: NL iSOD increases ≥ 5 mm<br>- NT NL: Visible growth<br>Note: Track nadir for target NL iSOD and total number of new lesions

TL, target lesion; NTL, non-target lesion; NL, new lesion; NT NL, non-target new lesion; iCPD, immune confirmed progressive disease; iCR, immune complete response; iPR, immune partial response; iSD, immune stable disease; iUPD, immune unconfirmed progressive disease; PD, progressive disease; SOD, sum of diameters for all target lesions; iSOD, immune sum of diameters for new lesion target.

Statistical considerations for defining the date of iRECIST PD:

- iUPD subsequently confirmed: The date used is the first iUPD date.
- iUPD never confirmed:
  - If a subsequent iSD, iPR or iCR is seen, the initial iUPD is ignored
  - Otherwise, iUPD date is used

Source: Seymour[4]

[redacted]

[redacted]

[redacted]

[redacted]	[redacted]	[redacted]	[redacted]
[redacted]			
[redacted]	[redacted]	[redacted]	[redacted]
[redacted]	[redacted]	[redacted]	[redacted]
[redacted]	[redacted]	[redacted]	
[redacted]	[redacted]	[redacted]	
[redacted]			
[redacted]	[redacted]	[redacted]	[redacted]
[redacted]	[redacted]	[redacted]	
[redacted]	[redacted]	[redacted]	[redacted]

[redacted]

12.6 Appendix: Management of Infusion Related Reactions

Patients will be monitored closely throughout the entire MP0317 infusion period and will also be questioned closely about any AEs that occur during the 24 hours after the infusion in order to capture any signs and symptoms of IRRs, their timing and recovery.

IRRs are defined by a group of signs and symptoms indicative of hypersensitivity reactions that occur during the 24 hours after the start of the MP0317 infusion and that the Investigator judges as IRRs (e.g. hypersensitivity, allergic reaction, anaphylactic reaction, nausea, vomiting, abdominal pain, headache, hypotension, pyrexia, tremor etc.).

Patients should be treated symptomatically with paracetamol (acetaminophen), ibuprofen (or another antipyretic drug), diphenhydramine (or another antihistamine drug) and/or cimetidine (or another H2 receptor antagonist) and, if considered necessary, corticosteroids, according to institutional standard practice. Serious IRRs manifested by dyspnea, hypotension, wheezing, bronchospasm, tachycardia, reduced oxygen saturation or respiratory distress should be managed with supportive care as clinically indicated (e.g. supplemental oxygen and E2-adrenergic agonists).

The Investigator will ensure that emergency drugs such as noradrenaline and hydrocortisone are readily available in case of IRRs. An emergency resuscitation trolley will also be readily available in case of anaphylactic shock or major cardiovascular events. Supportive care to manage any IRRs will be according to the site's standard-of-care and until the symptoms are resolved.

Guidance for management of IRRs and study treatment modifications are shown in Figure 4, Table 4 and below.

Prophylactic premedication to prevent IRRs may be administered at the discretion of the Investigator. During the second DERC meeting held on 9th March 2022, based on safety data from the first two q3w dosed cohorts, the DERC members strongly recommended prophylactic premedication of patients with antihistamine and paracetamol. Furthermore, based on safety information emerging during the course of the study, the DERC may consider mandating prophylactic premedication to manage IRRs, e.g., by using the regimen detailed below.

To better understand the pathogenesis of IRR, blood samples may be taken to evaluate levels of cytokines, complement factors, drug-specific IgE and tryptase (Table 7, Table 14 and Section 7.4.12).

Prophylactic Premedication

8 to 16 hours before scheduled MP0317 infusion

- Antihistamine as per SmPC (e.g. loratadine 10 mg, diphenhydramine 50 mg) PO
- Prednisone 1 mg/kg per os (PO)

60 minutes before scheduled MP0317 infusion

- Antihistamine as per SmPC (e.g. loratadine 10 mg, diphenhydramine 50 mg) PO or IV
- Paracetamol 1 g PO or IV

Optional: Prednisone 1 mg/kg PO or methylprednisolone 0.8 mg/kg IV

CONFIDENTIAL
May not be used, divulged, published, or otherwise disclosed without the consent of
Molecular Partners AG, Schlieren, Switzerland

Guidance on Re-start of Interrupted Infusion

1) Re-start at 50% of initial infusion rate for 30 minutes; continue with 2) if tolerated
2) Increase to 75% of initial infusion rate for 30 minutes; continue with 3) if tolerated or 1) if not tolerated
3) Increase to 100% of infusion rate; continue with 2) if not tolerated

Figure 4. Management of Infusion Related Reactions

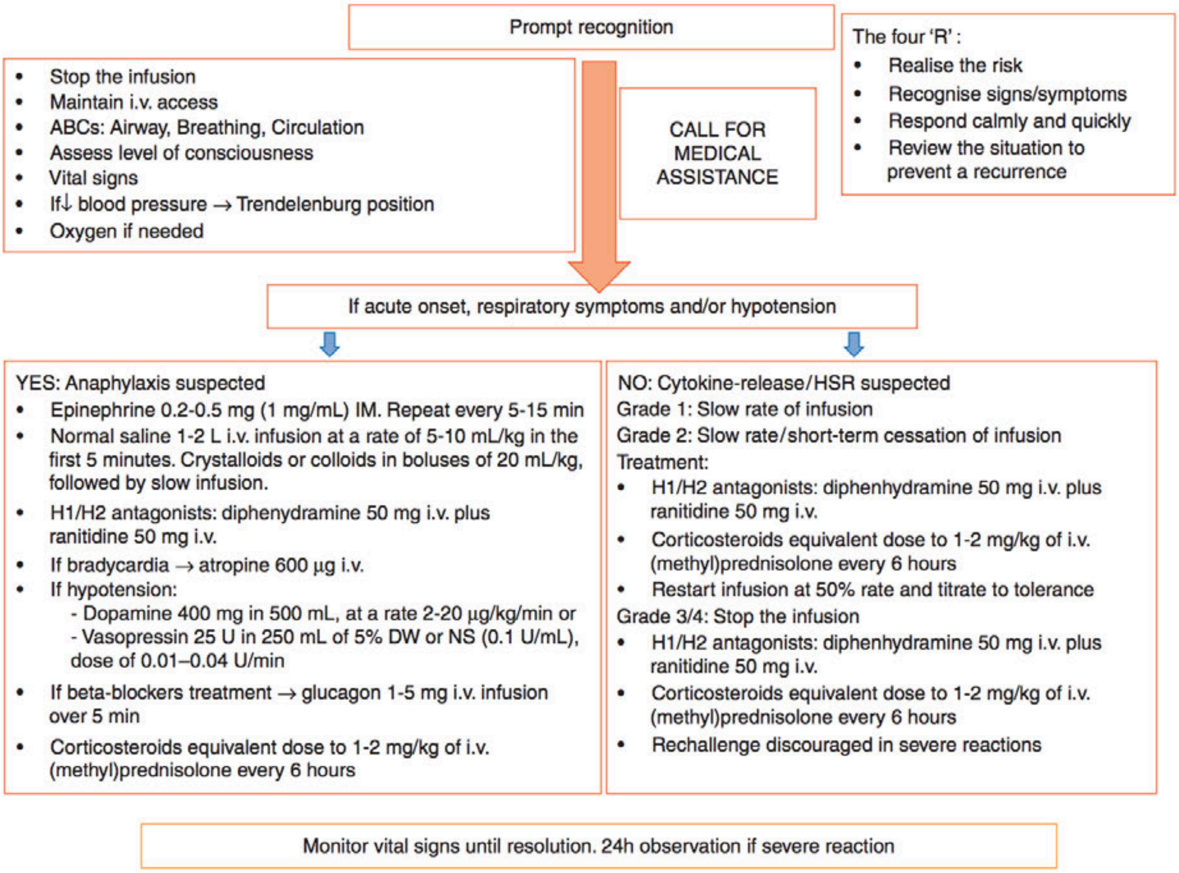

DW, dextrose water; HSR, hypersensitivity reaction; IM, intramuscular; i.v., intravenous; NS, normal saline; U, units.

Source: Rosello[48]

12.7 Appendix: Management of Cytokine Release Syndrome

Figure 5. Management of Cytokine Release Syndrome

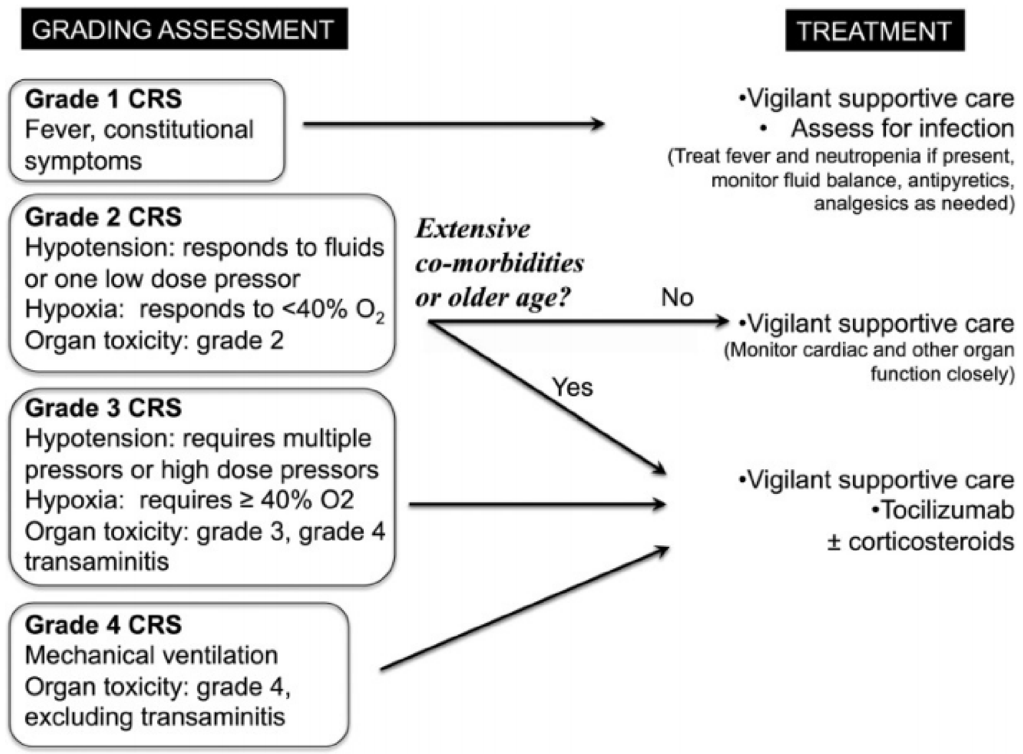

High-dose vasopressors (all doses are required for ≥3 hours)

Pressor	Dose
Norepinephrine monotherapy	≥20 µg/min
Dopamine monotherapy	≥10 µg/kg/min
Phenylephrine monotherapy	≥200 µg/min
Epinephrine monotherapy	≥10 µg/min
If on vasopressin	Vasopressin + norepinephrine equivalent of ≥10 µg/min*
If on combination vasopressors (not vasopressin)	Norepinephrine equivalent of ≥20 µg/min*

*VASST Trial vasopressor equivalent equation: norepinephrine equivalent dose = [norepinephrine (µg/min)] + [dopamine (µg/kg/min) ÷ 2] + [epinephrine (µg/min)] + [phenylephrine (µg/min) ÷ 10].

CRS, cytokine release syndrome; VASST, Vasopressin and Septic Shock Trial.

Source: Lee[49]

To better identify the pathogenesis of CRS, blood samples will be taken to evaluate levels of cytokines, complement factors, drug-specific IgE and tryptase (Table 7, Table 14 and Section 7.4.12).

12.8 Appendix: Statistical Model Performance and Data Scenarios

The model was assessed by two different metrics: Hypothetical on-study data scenarios and long-run operating characteristics.

Hypothetical Data Scenarios

Hypothetical data scenarios are shown in Table 30. These scenarios reflect potential on-study data constellations and related escalation as allowed by the model and the 233% escalation limit (i.e. next higher dose is not higher than 3.33-fold of the highest dose already tested). For each scenario, the probability of overdose for the current dose, as well as the next potential dose and related probabilities of underdosing, target dose and overdosing are shown.

Table 30. Hypothetical Data Scenarios

Setting 1: Describes the behavior if none in 3 patients experiences a DLT at any dose level. This shows that the BLRM then always recommends escalating.

Scenario	Dose mg/kg	Data - #DLT	Data - #Patient	CD - P(OD)	Next Higher Dose - P(OD)	Next Dose mg/kg	ND - P(UD)	ND - P(TD)	ND - P(OD)
1	0.03	0	3	0.005	0.014	0.10	0.934	0.0524	0.014
2	0.03	0	3						
	0.10	0	3	0.003	0.016	0.30	0.918	0.066	0.016
3	0.03	0	3						
	0.10	0	3						
	0.30	0	3	0.005	0.063	1.00	0.874	0.095	0.032
4	0.03	0	3						
	0.10	0	3						
	0.30	0	3						
	1.00	0	3	0.007	0.055	3.00	0.822	0.123	0.055
5	0.03	0	3						
	0.10	0	3						
	0.30	0	3						
	1.00	0	3						
	3.00	0	3	0.013	0.084	10.00	0.754	0.162	0.084
6	0.03	0	3						
	0.10	0	3						
	0.30	0	3						
	1.00	0	3						
	3.00	0	3						
	10.00	0	3	0.018		10.00	0.877	0.105	0.018

CONFIDENTIAL
May not be used, divulged, published, or otherwise disclosed without the consent of
Molecular Partners AG, Schlieren, Switzerland

Setting 2: Describes the behavior if 1 in 3 patients experiences a DLT at the current dose and 0 in 3 patients on lower dose levels. This shows that the BLRM recommends to expand the current dose cohort except for scenario 2 and 3 which would allow further escalation if no DLT in the lower dose cohort has been observed.

Scenario	Dose mg/kg	Data - #DLT	Data - #Patient	CD - P(OD)	Next Higher Dose - P(OD)	Next Dose mg/kg	ND - P(UD)	ND - P(TD)	ND - P(OD)
1	0.03	1	3	0.135	0.261	0.03	0.628	0.237	0.135
2	0.03	0	3						
	0.10	1	3	0.079	0.193	0.30	0.510	0.297	0.193
3	0.03	0	3						
	0.10	0	3						
	0.30	1	3	0.068	0.241	1.00	0.450	0.310	0.241
4	0.03	0	3						
	0.10	0	3						
	0.30	0	3						
	1.00	1	3	0.089	0.306	1.00	0.636	0.274	0.089
5	0.03	0	3						
	0.10	0	3						
	0.30	0	3						
	1.00	0	3						
	3.00	1	3	0.118	0.384	3.00	0.578	0.303	0.118
6	0.03	0	3						
	0.10	0	3						
	0.30	0	3						
	1.00	0	3						
	3.00	0	3						
	10.00	1	3	0.155		10.00	0.515	0.330	0.155

CONFIDENTIAL
May not be used, divulged, published, or otherwise disclosed without the consent of
Molecular Partners AG, Schlieren, Switzerland

Setting 3: Describes the behavior if 2 in 3 patients experience a DLT at the current dose and 0 in 3 patients on lower dose levels. In all scenarios the model recommends to de-escalate.

Scenario	Dose mg/kg	Data - #DLT	Data - #Patient	CD - P(OD)	Next Higher Dose - P(OD)	Next Dose mg/kg	ND - P(UD)	ND - P(TD)	ND - P(OD)
1	0.03	2	3	0.279	0.498	0.01	0.534	0.337	0.128
2	0.03	0	3						
	0.10	2	3	0.327	0.564	0.03	0.531	0.34	0.130
3	0.03	0	3						
	0.10	0	3						
	0.30	2	3	0.269	0.592	0.10	0.614	0.315	0.071
4	0.03	0	3						
	0.10	0	3						
	0.30	0	3						
	1.00	2	3	0.339	0.661	0.30	0.643	0.300	0.057
5	0.03	0	3						
	0.10	0	3						
	0.30	0	3						
	1.00	0	3						
	3.00	2	3	0.411	0.743	1.00	0.579	0.35 0	0.007
6	0.03	0	3						
	0.10	0	3						
	0.30	0	3						
	1.00	0	3						
	3.00	0	3						
	10	2	3	0.482		3.00	0.536	0.378	0.086

CONFIDENTIAL
May not be used, divulged, published, or otherwise disclosed without the consent of
Molecular Partners AG, Schlieren, Switzerland

Setting 4a: Describes the behavior if after extension of the last dose level according to Setting 2, 0 in 3 patients experience a DLT on the extended dose level. This shows that the BLRM recommend further escalation for all the scenarios. In scenarios 2 and 3, where escalation had been recommended, the BLRM recommends to further escalate.

Scenario	Dose mg/kg	Data - #DLT	Data - #Patient	CD - P(OD)	Next Higher Dose - P(OD)	Next Dose mg/kg	ND - P(UD)	ND - P(TD)	ND - P(OD)
1	0.03	1	3						
	0.03	0	3	0.036	0.116	0.10	0.619	0.265	0.116
2	0.03	0	3						
	0.10	1	3						
	0.30	0	3	0.059	0.145	1.00	0.480	0.312	0.208
3	0.03	0	3						
	0.10	0	3						
	0.30	1	3						
	1.00	0	3	0.074	0.174	3.00	0.44	0.318	0.242
4	0.03	0	3						
	0.10	0	3						
	0.30	0	3						
	1.00	1	3						
	1.00	0	3	0.024	0.18	3.00	0.512	0.308	0.180
5	0.03	0	3						
	0.10	0	3						
	0.30	0	3						
	1.00	0	3						
	3.00	1	3						
	3.00	0	3	0.037	0.244	10.00	0.444	0.312	0.244
6	0.03	0	3						
	0.10	0	3						
	0.30	0	3						
	1.00	0	3						
	3.00	0	3						
	10.00	1	3						
	10.00	0	3	0.051		10.00	0.695	0.255	0.051

CONFIDENTIAL
May not be used, divulged, published, or otherwise disclosed without the consent of
Molecular Partners AG, Schlieren, Switzerland

<u>Setting 4b:</u> Describes the behavior if after de-escalation according to Setting 3, 0 in 3 patients experiences a DLT on the de-escalated dose level. This shows that the BLRM recommend to escalate to an intermediate dose for the scenarios 1, 5 and 6 and recommends to re-escalate for the other scenarios.

Scenario	Dose mg/kg	Data - #DLT	Data - #Patient	CD - P(OD)	Next Higher Dose - P(OD)	Next Dose mg/kg	ND - P(UD)	ND - P(TD)	ND - P(OD)
1	0.03	2	3						
	0.01	0	3	0.279	0.421	0.02	0.416	0.37	0.214
2	0.03	0	3						
	0.10	2	3						
	0.03	0	3	0.184	0.422	0.10	0.417	0.399	0.184
3	0.03	0	3						
	0.10	0	3						
	0.30	2	3						
	0.10	0	3	0.168	0.492	0.30	0.424	0.408	0.168
4	0.03	0	3						
	0.10	0	3						
	0.30	0	3						
	1.00	2	3						
	0.30	0	3	0.241	0.592	1.00	0.334	0.425	0.241
5	0.03	0	3						
	0.10	0	3						
	0.30	0	3						
	1.00	0	3						
	3.00	2	3						
	1.00	0	3	0.308	0.573	2.00	0.410	0.427	0.163
6	0.03	0	3						
	0.10	0	3						
	0.30	0	3						
	1.00	0	3						
	3.00	0	3						
	10	2	3						
	3.00	0	3	0.376		6.50	0.352	0.433	0.215

BLRM, Bayesian Logistic Regression Model; CD, current dose; DLT, dose-limiting toxicity; ND, next dose; OD, overdose; TD, target dose; UD, underdose.

Operating Characteristics

Operating characteristics are a way to assess the long-run behavior of a model. Under an assumed true dose-toxicity curve, metrics such as the probability of recommending a dose with true DLT rate in the target interval can be approximated via simulation. Table 31 describes 3 assumed true dose-toxicity scenarios which were used to assess the operating characteristics of the model. These scenarios reflect a wide range of possible cases as follows:

- Scenario 1 (P): Aligned with prior means
- Scenario 2 (H): High-toxicity scenario
- Scenario 3 (LH): Low-toxicity followed by high-toxicity

Table 31. Assumed True Dose-Toxicity Scenarios

Scenario								
	Dose (mg/kg)	0.01	0.03	0.10	0.30	1.00	3.00	10
1(P)	P(DLT)	0.03	0.05	0.07	0.1	**0.16**	**0.23**	0.33
2(H)		0.1	**0.2**	**0.3**	0.4	0.5	0.6	0.7
3(LH)		0.05	0.1	0.15	**0.2**	**0.25**	0.4	0.45

Bold numbers indicate true DLT rates in the target interval [0.16-0.33).
DLT, dose-limiting toxicity; H, high-toxicity; LH, low-high-toxicity; P, probability.

For each of these scenarios, 500 studies were simulated. Thereby, in addition to the MTD definition in Section 5.2.7.2, the MTD is set to the RDE of 10 mg/kg (the highest considered dose) if 6 patients have been treated at 10mg/kg and the probability of overdosing is below 25% at this dose. On the other hand, if after at least 45 patients no MTD was found, the trial was stopped without declaring an MTD. It was then assessed how often a dose was declared as MTD with true DLT rate in the under-, targeted or over-dose range. Furthermore, the average, minimum and maximum number of patients per study and the average number of DLTs per study are reported. Results are shown in Table 32.

Table 32. Simulated Operating Characteristics

Scenario	% of Trials Declaring an MTD With True DLT Rate in			Max #Patient Reached Before MTD	Stopped	#Patients	#DLT
	Underdose	Target Dose	Overdose	% of Trials	% of Trials	Mean (min - max)	Mean (min - max)
1(P)	9.4	68.4	16.4	4.4	1.4	27.6 (3 - 45)	4.3 (2 - 10)
2(H)	2.4	61.0	20. 8	0	15.8	15.9 (3 - 33)	4.4 (2 - 9)
3(LH)	19.4	63.6	12	1.4	3.6	22.1 (3 - 45)	4.4 (2 - 10)

DLT, dose-limiting toxicity; H, high-toxicity; LH, low-high-toxicity; MTD, maximum tolerated dose; P, probability.

In Scenario 1, which reflects the case that the true dose-toxicity is aligned with prior means, 68.4% of the simulated trials declared a dose as MTD with true DLT rate in the targeted dose range.

CONFIDENTIAL
May not be used, divulged, published, or otherwise disclosed without the consent of
Molecular Partners AG, Schlieren, Switzerland

In Scenario 2 (high-toxicity scenario), the starting dose has already > 10% probability of observing at least 1 DLTs in the first cohort if 3 patients are enrolled. This contributes to the increased percentage of 15.8% of all simulated trials for which the trial is stopped since none of the doses are considered tolerable anymore. This is an expected situation for a high-toxicity scenario. Nonetheless, 61.0% of the simulated trials declared a dose as MTD with true DLT rate in the targeted dose range.

In Scenario 3, 19.4% of the simulated trials declared a dose as MTD with true DLT rate in the underdose dose range and in 1.4% of the trials, the MTD has not been found after 45 patients.

The proportion of trials where an overdose was declared as MTD was highest in scenario 2 with 20.8%. The mean patient numbers range from 15.9 patients (high scenario) to 27.6 patients (prior means scenario) and the maximum number of patients was 45, which is the maximum number of subject explored after which the simulation trials are stopped without declaring an MTD.

In summary, the considered data scenarios show a reasonable behavior of the model and the operating characteristics demonstrate a good precision of MTD determination.

CONFIDENTIAL
May not be used, divulged, published, or otherwise disclosed without the consent of
Molecular Partners AG, Schlieren, Switzerland

13 REFERENCES

[1] Dolznig, H. Characterization of cancer stroma markers: In silico analysis of an mRNA expression database for fibroblast activation protein and endosialin. *Cancer Immun* **5**, 10 (2005).

[2] Kratochwil, C.*, et al.* (68)Ga-FAPI PET/CT: Tracer Uptake in 28 Different Kinds of Cancer. *J Nucl Med* **60**, 801-805 (2019).

[3] Lo, A.*, et al.* Fibroblast activation protein augments progression and metastasis of pancreatic ductal adenocarcinoma. *JCI Insight* **2** (2017).

[4] Seymour, L.*, et al.* iRECIST: guidelines for response criteria for use in trials testing immunotherapeutics. *The Lancet Oncology* **18**, e143-e152 (2017).

[5] Eisenhauer, E.A.*, et al.* New response evaluation criteria in solid tumours: revised RECIST guideline (version 1.1). *Eur J Cancer* **45**, 228-247 (2009).

[6] Dadgostar, H., *et al.* Cooperation of multiple signaling pathways in CD40-regulated gene expression in B lymphocytes. *Proc Natl Acad Sci USA* **99**, 1497-1502 (2002).

[7] Gallagher, E., *et al.* Kinase MEKK1 is required for CD40-dependent activation of the kinases Jnk and p38, germinal center formation, B cell proliferation and antibody production. *Nat Immunol* **8**, 57-63 (2007).

[8] van Kooten, C., *et al*. CD40-CD40 ligand. *J Leukoc Biol* **67**, 2-17 (2000).

[9] Quezada, S.A., *et al*. CD40/CD154 interactions at the interface of tolerance and immunity. *Annu Rev Immunol* **22**, 307-328 (2004).

[10] Ferris, S.T., *et al*. cDC1 prime and are licensed by CD4(+) T cells to induce anti-tumour immunity. *Nature* **584**, 624-629 (2020).

[11] French, R.R., *et al*. CD40 antibody evokes a cytotoxic T-cell response that eradicates lymphoma and bypasses T-cell help. *Nat Med* **5**, 548-553 (1999).

[12] Sotomayor, E.M., *et al.* Conversion of tumor-specific CD4+ T-cell tolerance to T-cell priming through in vivo ligation of CD40. *Nat Med* **5**, 780-787 (1999).

[13] Vonderheide, R.H. CD40 agonist antibodies in cancer immunotherapy. *Annu Rev Med* **71**, 47-58 (2020).

[14] Vonderheide, R.H., *et al*. The immune revolution: a case for priming, not checkpoint. *Cancer Cell* **33**, 563-569 (2018).

[15] Morrison, A.H., *et al*. Sufficiency of CD40 activation and immune checkpoint blockade for T cell priming and tumor immunity. *Proc Natl Acad Sci USA* **117**, 8022-8031 (2020).

[16] Grilley-Olson, J.E., *et al.* SEA-CD40, a non fucosylated CD40 agonist: Interim results from a phase 1 study in advanced solid tumors. *Journal of Clinical Oncology* **36**, (15_suppl) 3093 (2018).

[17] Bendell, J.C.*, et al.* Phase 1 study to evaluate the safety and tolerability of the CD40 agonistic monoclonal antibody APX005M in subjects with solid tumors. *Journal of Clinical Oncology* **34**, (15 suppl) TPS3110 (2016).

[18] Vonderheide, R.H., *et al.* Clinical activity and immune modulation in cancer patients treated with CP-870,893, a novel CD40 agonist monoclonal antibody. *J Clin Oncol* **25**, 876-883 (2007).

CONFIDENTIAL
May not be used, divulged, published, or otherwise disclosed without the consent of
Molecular Partners AG, Schlieren, Switzerland

[19] Advani, R., *et al.* SGN-40 (AntihuCD40 mAb) monotherapy induces durable objective responses in patients with relapsed aggressive non-Hodgkin's lymphoma: evidence of antitumor activity from a Phase I Study. *Blood* **108**, 695 (2006).

[20] Ruter, J., *et al.* Immune modulation with weekly dosing of an agonist CD40 antibody in a phase I study of patients with advanced solid tumors. *Cancer Biol Ther* **10**, 983-993 (2010).

[21] Vonderheide, R.H., *et al.* Phase I study of the CD40 agonist antibody CP-870,893 combined with carboplatin and paclitaxel in patients with advanced solid tumors. *Oncoimmunology* **2**, e23033 (2013).

[22] Fransen, M.F., *et al*, Local activation of CD8 T cells and systemic tumor eradication without toxicity via slow release and local delivery of agonistic CD40 antibody. *Clin Cancer Res* **17**, 2270-2280 (2011).

[23] Knorr, D.A., *et al.* Toxicity of an Fc-engineered anti-CD40 antibody is abrogated by intratumoral injection and results in durable antitumor immunity. *Proc Natl Acad Sci USA* **115**, 11048-11053 (2018).

[24] Garin-Chesa, P., *et al.* Cell surface glycoprotein of reactive stromal fibroblasts as a potential antibody target in human epithelial cancers. *Proc Natl Acad Sci USA* **87**, 7235-7239 (1990).

[25] Busek, P., *et al.* Targeting fibroblast activation protein in cancer - Prospects and caveats. *Front Biosci (Landmark Ed)* **23**, 1933-1968 (2018).

[26] Rettig, W.J., *et al.* Cell-surface glycoproteins of human sarcomas: differential expression in normal and malignant tissues and cultured cells. *Proc Natl Acad Sci USA* **85**, 3110-3114 (1988).

[27] Denton, A.E., *et al.* Fibroblastic reticular cells of the lymph node are required for retention of resting but not activated CD8+ T cells. *Proc Natl Acad Sci USA* **111**, 12139-12144 (2014).

[28] Jansen, C.S., *et al.* An intra-tumoral niche maintains and differentiates stem-like CD8 T cells. *Nature* **576**, 465-470 (2019).

[29] Broomfield, S., *et al.* Partial, but not complete, tumor-debulking surgery promotes protective antitumor memory when combined with chemotherapy and adjuvant immunotherapy. *Cancer Res* **65**, 7580-7584 (2005).

[30] Kashyap, A.S., *et al.* Optimized antiangiogenic reprogramming of the tumor microenvironment potentiates CD40 immunotherapy. *Proc Natl Acad Sci USA* **117**, 541-551 (2020).

[31] Sum, E., *et al.* Fibroblast activation protein α-targeted CD40 agonism abrogates systemic toxicity and enables administration of high doses to induce effective antitumor immunity. *Clin Cancer Res*, Published Online First on March 26, 2021; DOI: 10.1158/1078-0432.CCR-20-4001.

[32] Labiano, S., *et al.* CD40 Agonist Targeted to Fibroblast Activation Protein α Synergizes with Radiotherapy in Murine HPV-Positive Head and Neck Tumors. *Clin Cancer Res*, April 26, 2021; DOI: 10.1158/1078-0432.CCR-20-4717.

[33] LYO-X-2021-01-0001 report. Pharmacokinetics and pharmacodynamics of MP0317 in cynomolgus monkey and syngeneic MC38 FAP-transduced mouse model of colon adenocarcinoma; prediction of exposure and effect in human (LyoX, Switzerland, 2021).

[34] Ristov, J*., et al.* Characterization of the in vitro and in vivo properties of CFZ533, a blocking and non-depleting anti-CD40 monoclonal antibody. *Am J Transplant* **18**, 2895-2904 (2018).

CONFIDENTIAL
May not be used, divulged, published, or otherwise disclosed without the consent of
Molecular Partners AG, Schlieren, Switzerland

[35] Espie, P., *et al.* First-in-human clinical trial to assess pharmacokinetics, pharmacodynamics, safety, and tolerability of iscalimab, an anti-CD40 monoclonal antibody. *Am J Transplant* **20**, 463-473 (2020).

[36] Vonderheide, R.H., et al. Clinical activity and immune modulation in cancer patients treated with CP-870,893, a novel CD40 agonist monoclonal antibody. J Clin Oncol 25, 876-883 (2007).

[37] Calvo, E., et al. A phase I study to assess safety, pharmacokinetics (PK), and pharmacodynamics (PD) of JNJ-64457107, a CD40 agonistic monoclonal antibody, in patients (pts) with advanced solid tumors. Journal of Clinical Oncology 37, 2527-2527 (2019).

[38] REP-0393-01: Distribution of FAP in various human tumors: Selection of indications for MP0317 treatment (MPAG, 2021).

[39] Guidance for Industry Drug-Induced Liver Injury: Premarketing Clinical Evaluation. *US Food and Drug Administration* (2009).

[40] Church, R.J., *et al.* In silico modeling to optimize interpretation of liver safety biomarkers in clinical trials. *Exp Biol Med (Maywood)* **243**, 300-307 (2018).

[41] Neuenschwander, S., *et al.* quantiNemo: an individual-based program to simulate quantitative traits with explicit genetic architecture in a dynamic metapopulation. *Bioinformatics* **24**, 1552-1553 (2008).

[42] Babb, J., *et al.* Cancer phase I clinical trials: efficient dose escalation with overdose control. *Stat Med* **17**, 1103-1120 (1998).

[43] Ponikowski, P., *et al.* 2016 ESC Guidelines for the diagnosis and treatment of acute and chronic heart failure: The Task Force for the diagnosis and treatment of acute and chronic heart failure of the European Society of Cardiology (ESC)Developed with the special contribution of the Heart Failure Association (HFA) of the ESC. *Eur Heart J* **37**, 2129-2200 (2016).

[44] Guideline on Clinical Trials in Small Populations. *European Medicines Agency* (2006).

[45] Rogatko, A., *et al.* Translation of innovative designs into phase I trials. *J Clin Oncol* **25**, 4982-4986 (2007).

[46] Schemper, M., *et al.* A note on quantifying follow-up in studies of failure time. *Control Clin Trials* **17**, 343-346 (1996).

[47] Oken, M.M., *et al.* Toxicity and response criteria of the Eastern Cooperative Oncology Group. *Am J Clin Oncol* **5**, 649-656 (1982).

[48] Rosello, S., *et al.* Management of infusion reactions to systemic anticancer therapy: ESMO Clinical Practice Guidelines. *Ann Oncol* **28**, iv100-iv118 (2017).

[49] Lee, D.W., *et al.* Current concepts in the diagnosis and management of cytokine release syndrome. *Blood* **124**, 188-195 (2014).

CONFIDENTIAL
May not be used, divulged, published, or otherwise disclosed without the consent of
Molecular Partners AG, Schlieren, Switzerland
